# mTORC2/Rac1 Pathway Predisposes Cancer Aggressiveness in *IDH1*-Mutated Glioma

**DOI:** 10.3390/cancers12040787

**Published:** 2020-03-26

**Authors:** Yang Liu, Yanxin Lu, Aiguo Li, Orieta Celiku, Sue Han, Mingyu Qian, Chunzhang Yang

**Affiliations:** Neuro-Oncology Branch, Center for Cancer Research, National Cancer Institute, Bethesda, MD 20892, USA; yang.liu5@nih.gov (Y.L.); yanxinlu@zmu.edu.cn (Y.L.); liai@mail.nih.gov (A.L.); orieta.celiku@nih.gov (O.C.); sue.han@nih.gov (S.H.); 201620434@mail.sdu.edu.cn (M.Q.)

**Keywords:** *IDH1* mutation, glioma, rac1, mTOR, Rictor, lamellipodia

## Abstract

Isocitrate dehydrogenase (*IDH*) mutations are common genetic abnormalities in lower grade gliomas. The neomorphic enzyme activity of IDH mutants leads to tumor formation through epigenetic alteration, dysfunction of dioxygenases, and metabolic reprogramming. However, it remains elusive as to how IDH mutants regulate the pathways associated with oncogenic transformation and aggressiveness. In the present study, by using unbiased transcriptomic profiling, we showed that *IDH1* mutations result in substantial changes in the gene sets that govern cellular motility, chemotaxis, and invasion. Mechanistically, rapamycin-insensitive companion of mammalian target of rapamycin (Rictor)/Ras-related C3 botulinum toxin substrate 1 (Rac1) signaling plays an essential role in the motility and proliferation of *IDH1*-mutated cells by prompting cytoskeleton reorganization, lamellipodia formation, and enhanced endocytosis. Targeting the Rictor/Rac1 pathway suppresses *IDH1*-mutated cells by limiting endocytosis and cell proliferation. Overall, our findings indicate a novel metabolic reprogramming mechanism of *IDH1*-mutated cells by exploiting metabolites from the extracellular milieu. Targeting the Rictor/Rac1 pathway could be an alternative therapeutic strategy for *IDH1*-mutated malignancies.

## 1. Introduction

Mutations in isocitrate dehydrogenase1/2 (*IDH1/2*) are common genetic defects in grade II/III diffusive astrocytoma and oligodendroglioma [1,2,3]. Cancer-associated *IDH* mutations commonly locate in an arginine residue at the center of the catalytic domain (IDH1 R132, IDH2 R140, and R172). Pathogenic IDH mutations catalyze α-ketoglutarate (α-KG) into D-2-hydroxyglutarate (D-2-HG), an oncometabolite closely related to the deactivation of α-KG-dependent dioxygenases [4,5]. For example, D-2-HG affects the catalytic function of DNA/histone demethylases, leading to the genome-wide hypermethylation and cancer stemness [6]. Moreover, *IDH* mutants disrupt the homeostasis of the Krebs cycle by depleting carbon-based metabolites, resulting in metabolic reprogramming and Warburg-like metabolism [7,8,9]. Despite the increasing awareness of the molecular mechanisms of IDH mutations on cancer metabolism and transformation, it remains elusive as to how metabolic deficient cancers, such as *IDH1*-mutated glioma, acquire an aggressive phenotype during oncogenic transformation.

Small guanosine triphosphatases (GTPases) are a family of hydrolases that consume GTP, which is essential for cell proliferation, differentiation, microtubule organization, migration, and vesicle transport. In glioma, small GTPases prompt oncogenic process, cancer cell survival, and invasion [10,11,12]. Recent findings also reveal the potential role of GTPase Rac1 in the maintenance of stemness and malignancies in glioma stem-like cells [13]. Activation of small GTPases assists tumor metabolism by regulating endothelial integrity and forming tumor blood vessels [14]. Targeting small GTPases, such as Rac1, showed a promising effect in suppressing glioblastoma xenografts [15]. However, the function of the small GTPase family in lower-grade gliomas, especially *IDH1*-mutated gliomas, is mostly unknown.

In the present study, we investigated the transcriptomic changes that resulted from the *IDH1* mutation. We identified that the acquisition of *IDH1* mutation leads to substantial enhancement in gene signatures associated with cellular motility, invasion, and chemotaxis. Rapamycin-insensitive companion of mammalian target of rapamycin (Rictor) as well as its concomitant downstream small GTPase, Rac1, play an essential role in promoting cytoskeleton reorganization, cellular motility, and endocytosis. Importantly, the prompted cell migration and endocytosis reprogrammed the food-seeking pattern in *IDH1*-mutated cancer cells, which, in return, supported tumor cell progression. Overall, our findings provide conceptual advances in understanding the role of small GTPases in *IDH1*-mutated glioma formation. Our study also indicates a novel therapeutic strategy for *IDH1*-mutated gliomas by targeting the small GTPase, Rac1.

## 2. Results

### 2.1. IDH1 Mutations Result in Robust Induction of Cellular Motility

**Figure 1:**
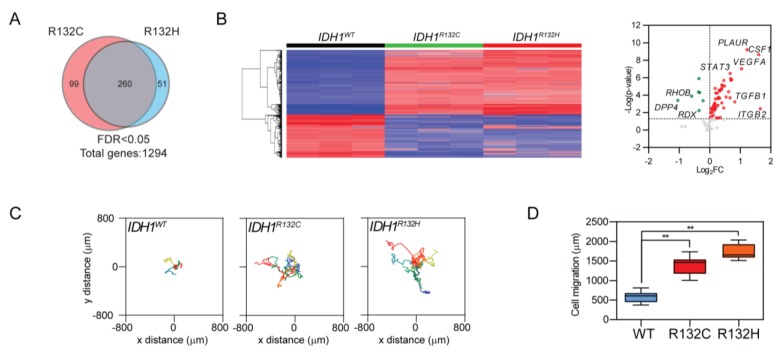
Enhanced cellular motility in *IDH1*-mutated cells. (**A**) Venn diagram showing the consistent transcriptomic change of genes that govern cellular motility, migration, and invasion when acquiring *IDH1* pathogenic mutations (R132C or R132H). A total of 260 genes were altered in both R132C and R132H cells (*n* = 3 for each genotype). (**B**) Hierarchical clustering analysis showed unique gene expression signature of cell movement acquired by *IDH1*-mutated cells (left panel). Targeted gene expression profiling showed up-regulated (red) or down-regulated (green) genes, shown as *IDH1^R132H^* vs. *IDH1^WT^* (right panel). (**C**) Live cell imaging showed enhanced cellular motility in *IDH1*-mutated U251 cells (*IDH1^WT^*, *n* = 8; *IDH1^R132C^*, *n* = 7; *IDH1^R132H^*, *n* = 8). (**D**) Quantification of cell migratory distance showed enhanced cellular motility in *IDH1*-mutated U251 cells (*IDH1^WT^*, 590.7 ± 44.89 μm, *n* = 8; *IDH1^R132C^*, 1390 ± 84.36 μm, *n* = 7; *IDH1^R132H^*, 1720 ± 73.17 μm, *n* = 8). *t*-test, ** *p* < 0.01.

To investigate the impact of *IDH1* mutation on cancer biology, we established *IDH1* mutation (*IDH1^R132C^* and *IDH1^R132H^*)-transduced U251 cell lines. The expression of the mutant enzyme was validated by immunoblotting and D-2-HG assay. *IDH1* mutation was confirmed by immunoblotting, Sanger sequencing, and D-2-HG assay. (Figure S1A–C). We analyzed the transcriptomic profiles of *IDH1*-mutated cells and identified 1294 differentially expressed genes (DEG) (Tables S1 and S3). Through Ingenuity pathway analysis, we recorded robust activation in gene sets that govern cellular motility, migration, and invasion (Figure 1A and Figure S1D, Tables S2 and S4). This trend was consistently recorded in both pathogenic *IDH1* mutants R132C and R132H. Hierarchical clustering analysis further indicated that the acquisition of *IDH1* mutation leads to substantial changes in the expression profile of cellular motility-related genes (Figure 1B, left panel). Targeted gene expression analysis showed that distinctive mRNA expression patterns for motility-related genes in *IDH1*-mutated cells exhibited up-regulation of colony stimulating factor 1 (*CSF1*), vascular endothelial growth factor A (*VEGFA*), Transforming growth factor beta 1 (*TGFB1*), and Signal transducer and activator of transcription 3 (*STAT3*), as well as down-regulation of Dipeptidyl peptidase-4 (*DPP4*)*,* Ras homolog gene family, member B (*RHOB*)*,* and Radixin (*RDX*, Figure 1B, right panel).

The distinctive mRNA expression pattern suggests altered cellular motility in cancer cells with *IDH1* mutation. We confirmed this hypothesis by tracking single-cell movement in a 12 h time-course imaging. As compared with *IDH1* wild type counterparts, *IDH1*-mutated cells exhibited remarkably different movement patterns, such as Levy walk-like movement, extended moving distance (Figure 1C,D). This phenomenon was also confirmed in U251 cells with doxycycline-inducible IDH1 R132H expression (Figure S2A). For further validation, we tested cellular migration in patient-derived brain tumor-initiating cell lines (BTIC) GSC923 (*IDH1^WT^*) and TS603 (*IDH1^R132H^*). Consistently, cellular migration and invasion was significantly enhanced in the *IDH1*-mutated cell line (Figure S2B–D).

### 2.2. Enhanced Cellular Motility Reprograms the Food-Seeking Pattern of IDH1-Mutated Cancer Cells

**Figure 2:**
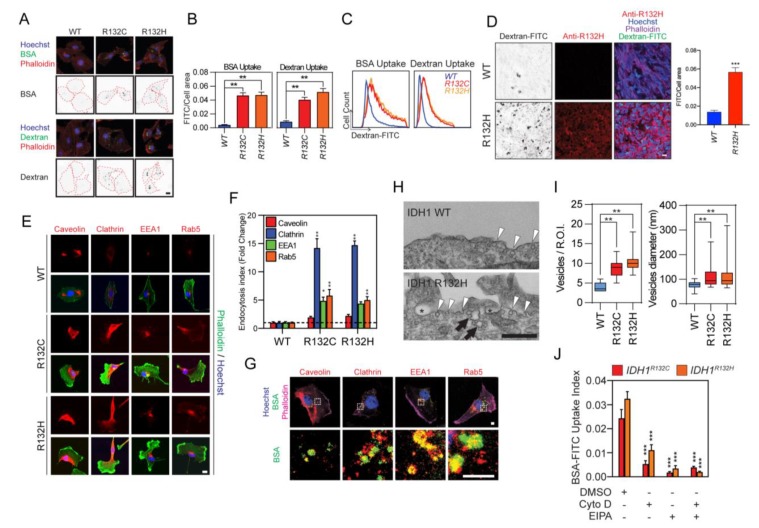
*IDH1*-mutated cells showed potentiated endocytosis. (**A**) Enhanced endocytosis in *IDH1*-mutated cells. Endocytosis was measured by high-molecular-mass fluorescein bovine serum albumin (BSA-FITC; green, upper panel) or high-molecular-mass fluorescein-dextran (dextran-FITC; green, lower panel) uptake assay. U251 *IDH1^R132C/H^* cells were probed with Hoechst 33,342 (blue) and Alexa Fluor 647-labeled phalloidin (red). Endocytosis vesicles were highlighted with a binary image. The cell boundary was labeled with a red dash line. Bar = 10 μm. Data quantification in Figure S3A. (**B**) Quantitative of BSA-FITC (*IDH1^WT^*, 0.004 ± 0.0004, *n* = 50; *IDH1^R132C^*, 0.047 ± 0.004, *n* = 39; *IDH1^R132H^*, 0.047 ± 0.004, *n* = 40) or dextran-FITC (*IDH1^WT^*, 0.009 ± 0.001, *n* = 57; *IDH1^R132C^*, 0.041 ± 0.004, *n* = 52; *IDH1^R132H^*, 0.052 ± 0.005, *n* = 48) uptake by quantitative image analysis for Figure 2A. *t*-test, ** *p* < 0.01. (**C**) Flow cytometry analysis of BSA-FITC or dextran-FITC uptake in U251 cells. (**D**) Dextran-FITC (green) uptake was measured in U251 *IDH1^WT^* or *IDH1^R132H^* xenografts in nude mice (*IDH1^WT^*, *n* = 2; *IDH1^R132H^*, *n* = 6); representative images are shown. IDH1 mutant was detected using an antibody targeting IDH1 R132H variant (red). The cell boundary was highlighted with phalloidin (purple). Dextran uptake was highlighted with a binary image. Cell nuclei were labeled with Hoechst 33,342 (blue). U251 *IDH1^WT^*: 0.014 ± 0.0017, *n* = 6; *IDH1^R132H^*: 0.057 ± 0.0048, *n* = 6. Bar = 20 μm. (**E**) Confocal microscopy showed endosomal markers caveolin, clathrin, Early Endosome Antigen 1 (EEA1), or Ras-related protein Rab-5 (Rab5, left panel). Quantification of endocytic vesicles in *IDH1* transduced cells. Endocytic vesicles were labeled with primary antibodies against caveolin, clathrin, EEA1, or rab5. Bar = 10 μm. (**F**) The formation of a vesicle was calculated by averaging the area of the vesicle to the total area of the cell. *IDH1^WT^*: caveolin, 1.00 ± 0.16; clathrin, 1.00 ± 0.23; EEA1, 1.00 ± 0.19; Rab5, 1.00 ± 0.12. *IDH1^R132C^*: caveolin, 1.94 ± 0.21; clathrin, 14.2 ± 1.63; EEA1, 4.87 ± 0.66; Rab5, 5.80 ± 1.06. *IDH1^R132H^*: caveolin, 2.23 ± 0.25; clathrin, 14.7 ± 0.73; EEA1, 4.38 ± 0.32; Rab5, 5.01 ± 0.61. (*n* = 5). *t*-test, * *p* < 0.05, ** *p* < 0.01. (**G**) Confocal microscopy showed colocalization of internalized BSA-FITC (green) with endosomal markers caveolin, clathrin, EEA1, or Rab5 (red) in U251 *IDH1^R132H^* cell. Cell boundary was highlighted with phalloidin (purple); cell nuclei were labeled with Hoechst 33,342 (blue). Bar = 1 μm. (**H**) Representative TEM imaging showing cell surface of *IDH1^WT^* (upper panel) and *IDH1^R132H^* (lower panel) U251 cell. Endocytosis (arrowheads), fused endocytic vesicles (arrows), and macropinocytosis (star) was highlighted in the image. Bar = 500 nm. (**I**) Quantitative analysis of endocytosis vesicles (*IDH1^WT^*, 4.00 ± 0.33, *n* = 12; *IDH1^R132C^*, 9.09 ± 0.73, *n* = 11; *IDH1^R132H^*, 10.5 ± 0.90, *n* = 11; ROI: region of interest) and the diameter of vesicles (*IDH1^WT^*, 76.2 ± 2.65, *n* = 31; *IDH1^R132C^*, 109 ± 5.53, *n* = 54; *IDH1^R132H^*, 105 ± 5.84, *n* = 49) in *IDH*1 mutant U251 cells based on TEM images. *t*-test, ** *p* < 0.01. (**J**) Immunofluorescence staining showed BSA-FITC uptake in *IDH1^R132C/H^* cells. Cells were pretreated with 5 μM cytochalasin D or 20 μM Ethylisopropyl amiloride (EIPA) for 2 h. Cells were incubated with BSA-FITC at a final concentration of 1 mg/mL for 60 min. IDH1^R132C^: Dimethyl sulfoxide (DMSO), 0.024 ± 0.0036, *n* = 35; Cytochalasin D (CytoD), 0.0053 ± 0.001, *n* = 51; EIPA, 0.0017 ± 0.0003, *n* = 52; CytoD + EIPA, 0.0039 ± 0.0005, *n* = 54. IDH1^R132H^: DMSO, 0.033 ± 0.0029, *n* = 42; CytoD, 0.011 ± 0.0022, *n* = 41; EIPA, 0.0034 ± 0.0012, *n* = 50; CytoD + EIPA, 0.0020 ± 0.0003, *n* = 51. *t*-test, *** *p* < 0.001.

Nutrient internalization plays critical roles in determining the cellular metabolic pattern, which confers the proliferation and survival advantages of tumor cells. It has been suggested that IDH mutant reprograms metabolic pathways by producing D-2-HG [16,17,18]. Enhanced motility and food-seeking pattern imply a novel metabolite acquisition pathway that is unique in *IDH*-mutated cells. To test this hypothesis, we monitored the endocytosis efficiency in *IDH1*-mutated U251 cells by feeding cells with fluorescence-labeled dextran or albumin. Confocal microscopy and flow cytometry analyses revealed that dextran or albumin uptake was increased by approximately 10-fold in *IDH1*-mutated cells (Figure 2A–C). Similarly, *IDH1^R132H^* U251 xenografts showed more dextran uptake as compared with *IDH1* wild type counterparts in nude mice (Figure 2D). As further validation, we identified the increased formation of caveolin^+^/clathrin^+^ endocytic vesicles and EEA1^+^/Rab5^+^ endosomes in *IDH1*-mutated cells (Figure 2E,F). Internalized bovine serum albumin (BSA) was found colocalized with subcellular components, such as caveolin^+^/clathrin^+^ endocytic vesicles and EEA1^+^/Rab5^+^ endosomes, indicating that extracellular substances are likely utilized in *IDH1*-mutated cells through the lysosomal pathway (Figure 2G). Moreover, electron microscopy showed the enhancement of endocytosis in *IDH1*-mutated cells, evidenced by an increase in quantity (2.27-fold for *IDH1^R132C^*; 2.61-fold for *IDH1^R132H^*) and diameter (76.2 nm for *IDH1^WT^;* 109.2 nm for *IDH1^R132C^*; 105.4 nm for *IDH1^R132H^*) of the endocytic vesicles in U251 cells with *IDH1* mutation (Figure 2H,I). The prompted endocytosis is likely associated with cytoskeleton mobilization and macropinocytosis, as actin inhibitor cytochalasin D or macropinocytosis inhibitor EIPA potently reduced BSA uptake in *IDH1*-mutated cells (Figure 2J). To further validate our findings, we also measured and quantified endocytosis in BTICs. Our results showed that TS603 cells with *IDH1^R132H^* mutant exhibited stronger expression of endosomal markers, as well as dextran uptake compared with *IDH1^WT^* GSC923 cells (Figure S3A,B).

### 2.3. Rac1-Mediated Cytoskeleton Reorganization is Essential for Reprogrammed Food-Seeking Pattern

**Figure 3:**
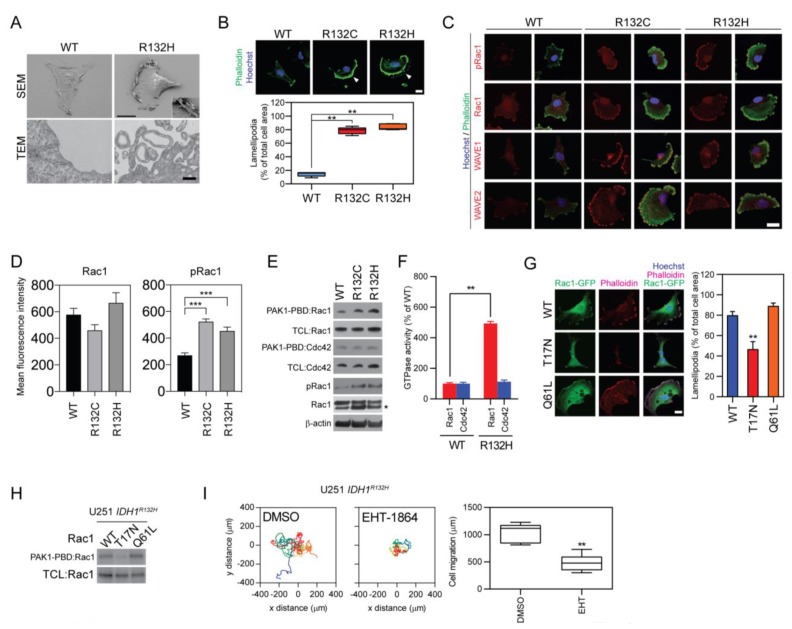
Rac1 activation support lamellipodia formation in *IDH1*-mutated cells. (**A**) SEM (upper panel, bar = 10 μm) and TEM (lower panel, bar = 1 μm) showing morphologic changes of *IDH1*-mutated cells. The inward folding plasma membrane is highlighted for *IDH1^R132H^* cells. (**B**) Co-staining of Hoechst 33,342 (blue) and phalloidin-AF488 (green) showed lamellipodia in *IDH1*-mutated cells (upper panel). Lamellipodia was highlighted with arrowheads. This experiment was repeated three times. Quantitative analysis showed significant increased lamellipodia formation (lower panel). *IDH1^WT^*, 9.42 ± 1.40, *n* = 12; *IDH1^R132C^*, 83.1 ± 1.60, *n* = 12; *IDH1^R132H^*, 87.2 ± 1.05, *n* = 12. Bar = 10 μm. *t*-test, ** *p* < 0.01. (**C**) Confocal microscopy showed colocalization of Rac1, GTP-bound Rac1 (Rac1-GTP, pRac1), Wiskott–Aldrich syndrome protein family member 1/2 (WAVE1/2, red) with F-actin-based lamellipodia (green). The cell nucleus was labeled by Hoechst 33342 (blue). Bar = 10 μm. This experiment was repeated three times. (**D**) Quantification of mean fluorescence intensity was performed for Rac1 and pRac1 in U251 *IDH1^WT^*, *IDH1^R132C^,* and *IDH1^R132H^*. *IDH1^WT^* cells were used as control. Rac1: *IDH1^WT^*, 579 ± 45.9; *IDH1^R132C^*, 460 ± 42.2; *IDH1^R132H^*, 667 ± 76.2.; pRac1: *IDH1^WT^*, 271 ± 17.8; *IDH1^R132C^*, 525 ± 19.2; *IDH1^R132H^*, 455 ± 27.9. *t*-test, *** *p* < 0.001. The mean fluorescence intensity was measured in five ROI for each group. (**E**) Rac/Cdc42 (p21) binding domain (PAK-PBD) pull-down assay and Western blot analysis showed increased active form Rac1 in *IDH1* mutated cells. * = nonspecific band. This experiment was repeated three times. (**F**) Quantification of the active form of Rac1 and Cell division control protein 42 homolog (Cdc42) in *IDH1*-mutated cell. Rac1 or Cdc42 was enriched by Glutathione S-transferase-bounded PAK-PDB protein. The quantity of the active form of GTPases was determined by Western blot and densitometry analysis. GTPase activity is shown as percentage changes of Rac1-GTP/Rac1 or Cdc42-GTP/Cdc42, respectively. Rac1: *IDH1^WT^*, 100 ± 7.09, *n* = 3; *IDH1^R132H^*, 494 ± 13.1, *n* = 3. Cdc42: *IDH1^WT^*, 100 ± 8.74, *n* = 3; *IDH1^R132H^*, 112 ± 11.6, *n* = 3. *t*-test, ** *p* < 0.01. (**G**) Confocal microscopy showed lamellipodia formation in the presence of Rac1 mutants (left panel) and quantification of cells with lamellipodia (right panel). Cells were labeled with phalloidin (purple), Enhanced green fluorescent protein bounded Rac1 (Rac1-EGFP, green), and Hoechst 33342 (blue). Bar = 10 μm. T17N, inactive mutation Rac1; Q61L, active mutation Rac1. WT: 79.6 ± 4.08, *n* = 11; T17N: 46.4 ± 7.86, *n* = 10; Q61L: 88.6 ± 3.34, *n* = 12. *t*-test, ** *p* < 0.01. (**H**) PAK-PDB pull-down assay showed reduced activated form of Rac1 when carrying the T17N variant. TCL, total cell lysate. (**I**) Live cell imaging showed decreased cellular migration in *IDH1^R132H^* U251 cells with 5 μM 5-(5-(7-(Trifluoromethyl)quinolin-4-ylthio)pentyloxy)-2-(morpholinomethyl)-4H-pyran-4-one dihydrochloride (EHT-1864) for 24 h (left panel). Cell migration measurement showed reduced cellular motility under EHT-1864 treatment (right panel, DMSO: 1038 ± 56.58, *n* = 9; EHT-1864: 480.3 ± 53.11, *n* = 8).

To investigate the molecular mechanism of enhanced endocytosis in *IDH1*-mutated cells, the cellular morphology of *IDH1*-mutated glioma U251 cells was first examined by scanning and transmission electron microscopies. Acquisition of *IDH1* mutation led to changes in cellular morphology, such as a fan-shaped cell body, inward-folding plasma membrane, and the establishment of lamellipodia (Figure 3A). The changes in cellular morphology are associated with the reorganization of the cytoskeleton. Phase-contrast microscopy and phalloidin labeling showed the condensed F-actin framework in the cutting-edge of the lamellipodia region, indicating a reorganization of the cytoskeleton in the *IDH1*-mutated cells, which might be mediated by small GTPase activation (Figure 3B). Accordingly, immunofluorescence staining showed that Rac1-GTP (pRac1), the GTP-bound active form of small GTPase Rac1, was highly expressed in *IDH1*-mutated cells and accumulated in the F-actin-enriched lamellipodia region. Moreover, WAVE1/2, the downstream effectors for Rac1, also accumulated in the same region of the cells (Figure 3C,D). The activation of Rac1 was further validated by a GST pull-down assay. The activated form of small GTPases was enriched by the GST-conjugated PBD domain from PAK1 and was labeled through immunoblotting. Results showed that the active form of Rac1, but not Cdc42, was selectively accumulated in *IDH1*-mutated cells (Figure 3E,F).

Further, we showed that the Rac1 activity is essential for the changes in cellular morphology and motility through a loss-of-function assay. The formation of F-actin-enriched lamellipodia reduced by 41.8% in *IDH1^R132H^* cells with the expression of a dominant-negative recombinant of Rac1 (T17N), but not the active mutation of Rac1 (Q61L, Figure 3G,H). Moreover, cellular motility was found to be compromised by Rac1 inhibitor EHT-1864, suggesting that the changes in the motility of *IDH1*-mutated cells require activation of Rac1 (Figure 3I).

### 2.4. Rictor/Rac1 Pathway Governs the Endocytosis Pathway in IDH1-Mutated Cells

**Figure 4:**
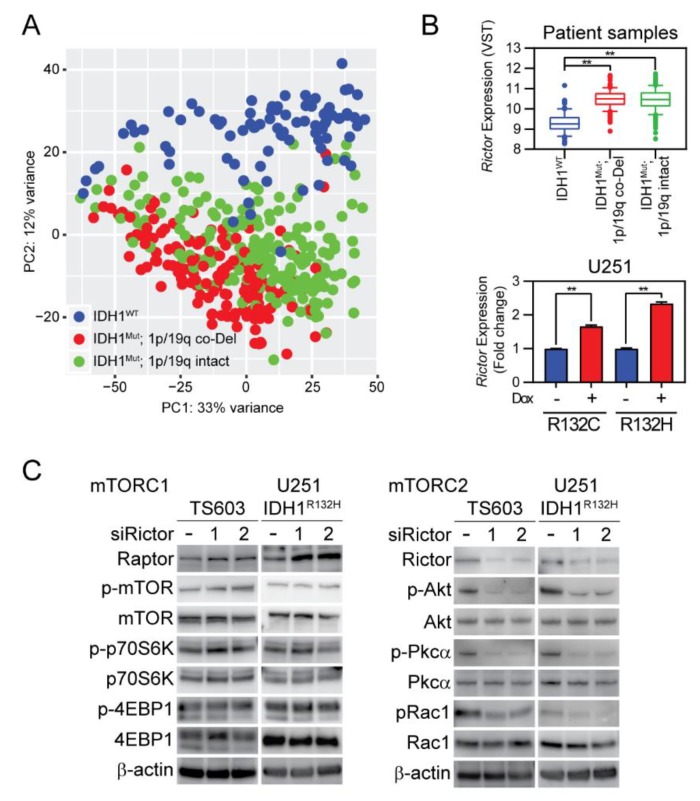
Rapamycin-insensitive companion of mammalian target of rapamycin (Rictor)/Rac1 enhanced mTORC2 pathway in IDH1-mutated cells. (**A**) The principal component analysis showed a distinct transcriptomic pattern of expression in IDH-mutant gliomas. Whole transcriptomic profiling data from 367 *IDH*-mutant and 85 *IDH*-WT patient samples from The Cancer Genome Atlas (TCGA) were used in the analysis. Diffusive astrocytoma (1p/19q intact) and oligodendroglioma (1p/19q co-deleted) were specified in green and red, respectively. (**B**) Enhanced Rictor expression in *IDH1*-mutated tumors and cells. Rictor expression from TCGA data (upper panel), *IDH1^WT^*: 9.30 ± 0.06, *n* = 85; *IDH1^mut^* 1p/19q intact: 10.5 ± 0.04, *n* = 215; *IDH1^mut^* 1p/19q Co-deletion (CoDel): 10.5 ± 0.04, *n* = 151. VST: variance-stabilizing transformation; Rictor expression as measured by quantitative PCR analysis in U251 cells (lower panel). R132C: without doxycycline (-DOX), 1.00 ± 0.002, *n* = 3; +DOX, 1.66 ± 0.03, *n* = 3. R132H: -DOX, 1.00 ± 0.02; +DOX, 2.34 ± 0.03, *n* = 3. *t*-test, ** *p* < 0.01. Diffusive astrocytoma (1p/19q intact) and oligodendroglioma (1p/19q co-deleted) were specified in green and red, respectively. (**C**) Western blot analysis showed the effect of RNA interference targeting Rictor. Suppressing Rictor expression minimally influenced mTORC1 downstream p70S6K or 4EBP1, whereas mTORC2 downstream Akt, Pkcα, and Rac1 phosphorylation were reduced. This experiment was repeated three times.

Small GTPases are mainly regulated by their upstream factors, such as guanine nucleotide exchange factors (GEFs), GTPase-activating proteins (GAPs), or guanine nucleotide dissociation inhibitors (GDIs). Additionally, small GTPases could be regulated by kinases such as mammalian target of rapamycin complex 2 (mTORC2), which facilitates Rac1 activation by suppressing Rho GDP-dissociation inhibitor 2 (RhoGDI2) [19,20,21]. Although we recorded Rac1-guided cellular motility in *IDH1*-mutated cells, a key question remains elusive with regards to how Rac1 is regulated in this type of cancer. To better understand the upstream signaling pathways of Rac1, we compared the transcriptomic profile of glioma specimens from the lower grade glioma dataset from the Cancer Genome Atlas Program (TCGA). We stratified these tumor specimens by their *IDH1* mutation status and focused on the DEGs. Principal component analysis (PCA) suggested that *IDH1*-mutated tumors exhibit an altered transcriptome signature from their wild type counterparts (Figure 4A). Importantly, we identified that *IDH1* mutation status correlates with remarkably higher expression of Rictor in both patient samples and transduced U251 cells (Figure 4B). Additionally, the upregulation of Rictor in *IDH1*-mutated U251 cells and BTICs correlated with enhanced mTORC2 downstream, such as phosphorylation of Ak strain transforming (Akt) and Protein kinase C alpha (PKCα), whereas the mTORC1 downstream, such as ribosomal protein S6 kinase beta-1 (p70S6K) and eukaryotic translation initiation factor 4E-binding protein 1 (4EBP1) phosphorylation, were not altered with *IDH1* mutation (Figure S4A,B). Further, we performed a loss-of-function study for Rictor using small interference RNA in both BTIC cells and transduced U251 cells. mTORC1 downstream, such as p70S6K and 4EBP1, was minimally affected by genetic silencing of Rictor, whereas mTORC2 downstream signaling, such as Akt, Pkcα, and Rac1, was found to be suppressed in *IDH1*-mutated cells (Figure 4C, Figure S3E, and Figure S4C).

**Figure 5:**
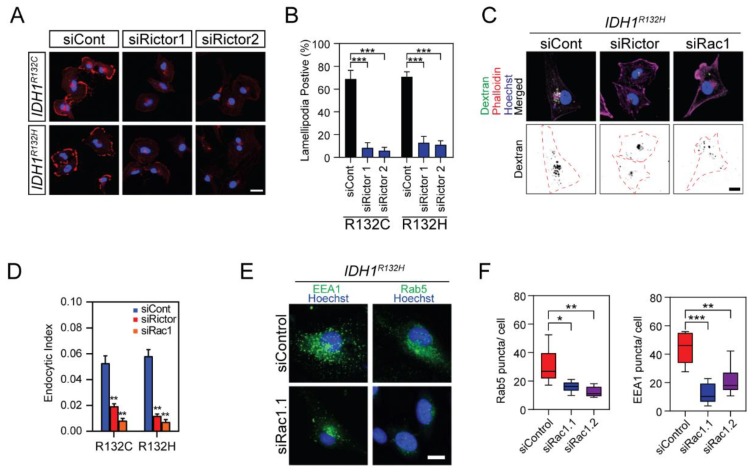
Knockdown of Rictor suppressed lamellipodia formation and endocytosis of IDH1-mutated cells. (**A**) Confocal microscopy showed lamellipodia formation in *IDH1*-mutated cells with Rictor inhibition. Cells were labeled with Hoechst 33,342 (blue) and Alexa Fluor 647-labeled phalloidin (red). Bar = 10 μm. (**B**) Quantitative analysis showed decreased lamellipodia presenting cells with Rictor inhibition. R132C: control RNA (siCont), 68.8 ± 7.75; siRNA targeting Rictor (siRictor1), 8.45 ± 4.52; siRictor2, 5.95 ± 2.92. R132H: siCont, 70.7 ± 4.43; siRictor1, 13.0 ± 5.42; siRictor2, 11.1 ± 3.50. *t*-test, *** *p* < 0.001. The lamellipodia-positive cells were measured in eight ROI for each group. (**C**) Confocal microscopy showed dextran endocytosis after inhibition of Rac1 or Rictor in U251 *IDH1^R132H^* cells. Cells were labeled with Hoechst 33,342 and Alexa Fluor 647-labeled phalloidin. Bar = 10 μm. (**D**) Quantification of dextran endocytosis was calculated by averaging the area of the vesicle to the total area of the cell in U251 *IDH1*-mutated cells. R132C: siCont, 0.053 ± 0.006, *n* = 28; siRictor, 0.019 ± 0.002, *n* = 25; siRac1, 0.008 ± 0.002, *n* = 21. R132H: siCont, 0.058 ± 0.005, *n* = 16; siRictor, 0.012 ± 0.001, *n* = 21; siRac1, 0.007 ± 0.002, *n* = 20. *t*-test, ** *p* < 0.01. (**E**) Confocal microscopy of EEA1 +/Rab5a+ endosome (green) in U251 *IDH1^R132H^* cells after inhibition of Rac1. (**F**) Quantification of Rab5 and EEA1 puncta per cell. Rab5: siCont, 30.3 ± 5.05, *n* = 6; siRac1.1, 16 ± 1.56, *n* = 6; siRac1.2, 12.3 ± 1.51, *n* = 6. *t* test, * *p* < 0.05, ** *p* < 0.01, *** *p* < 0.001.

By mediating Rac1 phosphorylation, Rictor is key to the cytoskeleton reorganization and food-seeking pattern in *IDH1*-mutated cells. The formation of F-actin-enriched lamellipodia was reduced by approximately 75% in *IDH1*-mutated cells when treated with small interference RNA targeting Rictor (Figure 5A,B). Moreover, the invasive capability of *IDH1*-mutated BTIC cells was significantly impaired by Rac1 or Rictor knockdown (Figure S2E). These results suggested that the Rictor/Rac1 pathway is vital for the enhanced cellular motility pattern associated with *IDH1* mutation. Moreover, the uptake of dextran was found to be limited when Rictor or Rac1 was suppressed, indicating that the Rictor/Rac1 pathway assists endocytosis in *IDH1*-mutated cells (Figure 5C,D and Figure S3C). Accordingly, Rac1 small interference RNA reduced the formation of the EEA1/Rab5 endosome in *IDH1*-mutated cells (Figure 5E,F). Moreover, Rac1 and Rictor suppression led to cell viability decrease in *IDH1*-mutated U251 cells and BTIC TS603, but not in *IDH1* wild type U251 cells and BTIC GSC923 (Figure S3D), which indicates that targeting of the mTORC2/Rac1 pathway might be a potential therapeutic approach for cancers with *IDH1* mutations.

### 2.5. Targeting mTORC2/Rac1 Pathway Selectively Suppresses IDH1-Mutated Glioma by Limiting Endocytosis

**Figure 6:**
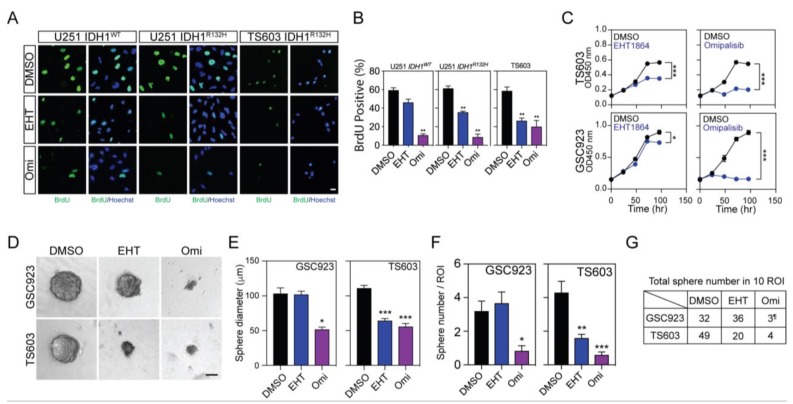
IDH1-mutated cancer cells exhibited selective vulnerability to Rac1 inhibition. (**A**) BrdU (green) incorporation assay showed cellular proliferation with 5 μM EHT-1864 or 1 μM omipalisib treatment for 72 h. Nuclei were labeled with Hoechst (blue). Bar = 10 μm. (**B**) Quantification of BrdU cell proliferation assay in Figure 6A. U251 *IDH1^WT^*: DMSO, 59.4 ± 2.61, *n* = 7; EHT-1864 (EHT), 46.1 ± 3.60, *n* = 5; omipalisib (Omi), 10.8 ± 1.48, *n* = 6. U251 *IDH1^R132H^*: DMSO, 61.3 ± 2.54, *n* = 6; EHT, 35.5 ± 1.29, *n* = 6; Omi, 8.77 ± 3.29, *n* = 6. TS603: DMSO, 58.6 ± 4.26, *n* = 6; EHT, 26.3 ± 2.97, *n* = 6; Omi, 20.1 ± 6.65, *n* = 6. *t*-test, ** *p* < 0.01. (**C**) Cell proliferation test for GSC923 and TS603 cells under 5 μM EHT-1864 or 1 μM omipalisib treatment. This experiment was repeated three times. TS603: DMSO, 0.566 ± 0.009, *n* = 3; EHT-1864, 0.351 ± 0.011, *n* = 3; DMSO, 0.547 ± 0.010, *n* = 3; Omi, 0.203 ± 0.023, *n* = 3. GSC923: DMSO, 0.880 ± 0.039, *n* = 3; EHT-1864, 0.732 ± 0.009, *n* = 3; DMSO, 0.896 ± 0.034, *n* = 3; Omi, 0.164 ± 0.009, *n* = 3. *t*-test, * *p* < 0.05, *** *p* < 0.001. (**D**) Sphere formation assay using brain tumor-initiating cell lines (BTIC) TS603 and GSC923 with 5 μM EHT-1864 or 1 μM omipalisib treatment for 2 weeks. Bar = 50 μm. (**E**) Quantification of sphere size by diameter of cell spheres in Figure 6D. GSC923: DMSO, 103 ± 8.15, *n* = 32; EHT, 102 ± 4.27, *n* = 33; Omi, 52.1 ± 3.10, *n* = 5. TS603: DMSO, 111 ± 3.91, *n* = 43; EHT, 64.4 ± 3.02, *n* = 16; Omi, 56.0 ± 4.28, *n* = 5. *t*-test, * *p* < 0.05, ** *p* < 0.01, *** *p* < 0.001. (**F**) Quantification of sphere number per ROI from sphere formation assay in Figure 6D. GSC923: DMSO, 3.20 ± 0.593, *n* = 10; EHT, 4.00 ± 0.645, *n* = 9; Omi, 0.50 ± 0.224, *n* = 6. TS603: DMSO, 4.80 ± 0.998, *n* = 10; EHT, 1.90 ± 0.314, *n* = 10; Omi, 0.40 ± 0.163, *n* = 10. *t*-test, * *p* < 0.05, ** *p* < 0.01, *** *p* < 0.001. (**G**) The total number of spheres that were measured in 10 ROI (right panel). ¶, the spheres of GSC923 omipalisib group was measured in six ROI.

This finding of mTOR2/Rac1 activation suggests a distinct means of nutrient acquisition in *IDH1*-mutated cells. It also implies a potential therapeutic strategy for *IDH1*-mutated glioma by suppressing Rictor/Rac1-governed endocytosis. To test this hypothesis, we evaluated cell proliferation and viability in the presence of chemical inhibitors to Rac1 or mTOR. Bromodeoxyuridine (BrdU) incorporation assay showed that the Rac1 inhibitor EHT-1864 selectively suppressed the cellular proliferation of *IDH1*-mutated U251 and TS603 cells. mTOR inhibitor omipalisib, which potently suppresses both mTORC1 and two activities, showed suppression of cell proliferation in both *IDH1* wild type and mutated cells (Figure 6A,B), which indicated that mTOR signaling plays essential roles in both glioma molecular subtypes. We further validated this finding through Cell Counting Kit-8 (CCK8) cell viability assay and sphere formation assay using patient-derived BTICs. Consistently, results showed that Rac1 inhibition by EHT-1864 led to suppression in cells with *IDH1* mutations, whereas total mTOR inhibition by omipalisib showed potent suppression in both *IDH1* wild type and mutant cells (Figure 6C–G). 

## 3. Materials and Methods

### 3.1. DNA Cloning and Mutagenesis

Coding sequences of human *IDH1* or *IDH2* were inserted into a pLenti-C-myc-DDK-IRES-Puro vector (Origene, Rockville, MD, USA) and pLVX-TetONE-Puro vector (Clontech, Mountain View, CA, USA). Pathogenic *IDH1* variants (R132C, R132H) were generated by using the Quikchange lightning mutagenesis kit (Agilent, Santa Clara, CA, USA). The coding sequences of *IDH1* or *IDH2* were verified by sequencing the entire region of coding sequences of each vector.

### 3.2. Lentivirus Packaging and Infection

Lentivirus particles were packaged using the Lenti-X 293T cell line (Clontech, Mountain View, CA, USA). IDH1/2 coding transfer plasmids were co-transfected with pMD2.G (Addgene 12259, Watertown, MA, USA) and psPAX2 (Addgene 12260, Watertown, MA, USA) using lipofectamine 3000 according to the manufacturer’s protocol (Invitrogen, Carlsbad, CA, USA). The supernatant was collected 24 and 48 h after transfection. Virus particles were enriched using a Lenti-X concentrator (Clontech, Mountain View, CA, USA) and stored at −80 °C.

### 3.3. Cell Culture

Human glioma cell line U251 MG was purchased from Sigma in 2015. Cells are maintained in Minimum Essential Medium alpha (MEMα) medium supplemented with 10% fetal bovine serum (Thermo Fisher, Waltham, MA, USA) and 1% antibiotics (100 U/mL penicillin and 10 μg/mL streptomycin) at 37 °C in humidified air with 5% CO_2_. *IDH1* mutation transduced U251 cells, and doxycycline-induced *IDH1* mutation U251 cells were generated by lentivirus infection. Cells were selected with puromycin, and the expression of IDH1 was characterized by both Western blot and Sanger sequencing for the entire coding region of *IDH1* genes. Brain tumor-initiating cell (BTIC) TS603 was a kind gift from Dr. Timothy Chan from the Memorial Sloan Kettering Cancer Center [6,22]. BTIC line GSC923 was derived from a patient sample following the approval of the National Cancer Institute Institutional Review Board [23]. All BTIC lines were cultured in Neurobasal-derived (NBE) media, as previously described [24]. In some experiments, BTIC cell lines were attached to a Geltrex-coated culture flask for microscopy assays. All cell lines passed the mycoplasma contamination test.

### 3.4. D-2-HG Assay

The cellular levels of D-2-HG was determined by assay kit (BioVision, Milpitas, CA, USA). Cells were homogenized in ice-cold D-2-HG assay buffer and incubated with subtrate mix and assay enzyme. The reaction was incubated at 37 °C for 1 h. The quantity of D-2-HG was quantified on a multi-well spectrophotometer. 

### 3.5. RNA Preparation and Sequencing

Total RNA was purified using an RNeasy mini kit (QIAGEN, Hilden, Germany) according to the manufacturer’s instructions, and the integrity of the RNA was assessed using an RNA 6000 pico kit on an Agilent 2100 Bioanalyzer. The RNA samples with RNA integrity numbers (RINs) greater than 9.0 were submitted to ACGT, Inc (http://www.acgtinc.com/) for sequencing, and paired-end sequencing was performed with 125 bp read length. 

### 3.6. RNASeq Data Analysis

The RNA sequencing (RNASeq) data were processed using a next-generation sequencing (NGS) data analysis pipeline, CCBR Pipeliner, developed by National Cancer Institute (NCI) Collaborative Bioinformatics Resource (CCBR) (https://github.com/CCBR/Pipeliner) and deployed on the National Institutes of Health (NIH) Biowulf server (https://helix.nih.gov). In brief, CCBR Pipeliner trims the sequencing adaptors using Trimmomatic and aligns RNASeq reads to the human genome to build human genome version 19 (hg19) using Spliced Transcripts Alignment to a Reference (STAR) algorithms. The quantification of RNA expression was performed using the Subread algorithm [25]. Before deriving the differentially-expressed genes (DEG), genes were filtered out if they did not have more than five counts per million (CPM) in at least two samples. Three comparative analysis algorithms (EdgeR, DEseq2, and voom/Limma) are used in the CCBR Pipeliner to derive fold change of expression and false discovery rate (FDR)-adjusted *p*-values. Genes with FDR less than or equal to 0.05 and an absolute fold change greater than 2 in all three methods were considered DEGs. The supervised hierarchical clustering was carried out using Partek Genomics Suite version 6.6 (www.partek.com). The expression values for supervised hierarchical clustering (HC) were normalized by the EdgeR algorithm via CCBR Pipeliner. The complete linkage method and Euclidean distance were used for the hierarchical clustering analysis. The Venn diagram was generated using the R package venndiagram [26].

### 3.7. Functional Analyses

Commonly-identified DEGs from all three methods for all the contrasts were uploaded into QIAGEN’s Ingenuity Pathway Analysis (IPA, QIAGEN Redwood City, CA, USA, www.qiagen.com/ingenuity). IPA core analysis was performed to assess the affected pathways and functions by IDH mutations. Curated pathways and functions from the IPA Knowledge Base were considered significantly enriched on the basis of a *p*-value less than 0.05 or z-score higher than 1.5, as defined in IPA. 

### 3.8. Real-time PCR

Total RNA was reversely transcribed into complementary DNA (cDNA) library using SuperScirpt IV VILO Master Mix (Thermo Fisher, Waltham, MA, USA). Gene expression was analyzed by RT^2^ Profiler PCR Array (PAHS-128Z, QIAGEN, Redwood City, CA, USA). Data were acquired using a QuantStudio 7 Flex Real-Time PCR System (Thermo Fisher, Waltham, MA, USA) and plotted using GraphPad Prism software (ver. 7.01, La Jolla, CA, USA).

### 3.9. Live Cell Imaging

Live cell imaging was performed using a Nikon BioStation IM-Q. Cells were seeded on poly-D-lysine coated 35 mm glass-bottom culture dish. The dish was placed in the culture chamber with 37 °C and humidified air with 5% CO_2_ supply. The phase-contrast image was taken every 15 min for 24 hr using a 10x objective lens. Data were analyzed by the manual tracking feature in ImageJ software (https://imagej.nih.gov/ij/, National Institutes of Health, Bethesda, MD, USA). In some assays, EHT-1864 (5 μM, Cayman Chemical, Ann Arbor, MI, USA) was added into the tissue culture media.

### 3.10. Boyden Chamber Assay

Transwell inserts were pre-coated with laminin (10 μg/mL) overnight at 4 °C. For migration assay, 50,000 cells were resuspended in 300 μL serum-free media and seeded on the insert (diameter 6.5 mm, pore size 8 μm; Corning, Corning, NY, USA). Five hundred microliters of growth media containing 10% fetal bovine serum was added to the lower chamber. The transwell was incubated for 8–12 h. After incubation, growth media was aspirated, and cells from the upper chamber were removed by a cotton swab. The transwell membrane was then stained in 1% crystal violet solution (Sigma, St. Louis, MO, USA) for 20 min at room temperature. The membrane was then washed in Phosphate-buffered saline (PBS) and dried for imaging. Six random images were taken by an inverted microscope, and migrated cells were marked out using the “Point selection” tool, and the number of cells was measured by the “Analyze→Measure” tool in ImageJ (National Institutes of Health, Bethesda, MD, USA). For invasion assay, the upper chamber was pre-coated with Matrigel (Corning, Corning, NY, USA) according to the manufacturer’s protocol, and incubation time may have been prolonged to 16–20 h.

### 3.11. 3D Invasion Assay

A total of 4000–5000 cells were seeded in an 96-well ultra-low attachment round bottom plate (Corning, Corning, NY, USA) with 100 μL culture media. The plate was then centrifuged for 10 min at 300× *g* to pellet the cells at the center of the well bottoms. The cells were incubated at 5% CO_2_ and 37 °C for 3 days to form a single sphere. Once the cells formed spheres, 100 μL of Matrigel matrix (Corning, Corning, NY, USA) was added to each well. Cells were incubated for up to 7 days. Images were taken at multiple time points. The invasive capability of cells was quantified by measuring the area of the spheres using ImageJ (National Institutes of Health, Bethesda, USA).

### 3.12. Quantification of Endocytosis

Endocytic particles were marked using high-molecular-mass fluorescein-dextran (dextran-FITC) or bovine serum albumin (BSA-FITC) by uptake assay as previously described [27]. In brief, FITC-dextran or FITC-BSA were added to serum-starved cells at a final concentration of 1 mg/mL for 60 min. For confocal microscopy, cells were rinsed five times with cold PBS and fixed in 4% paraformaldehyde. Cells were then stained with Hoechst 33,342 and Alexa Fluor 647-conjugated phalloidin (Invitrogen, Carlsbad, CA, USA) for 30 min. Images were captured using a Zeiss laser-scanning microscope (LSM) LSM880 or LSM 710 confocal microscope and analyzed using ImageJ (National Institutes of Health, Bethesda, MD, USA), as previously described [27]. Briefly, the endocytic particle area was determined using the “Image→Adjust→Threshold” tool, followed by the “Analyze→Analyze Particles” tool. The total cell area was selected using the “Polygon selections” tool and followed by the “Analyze→Measure” tool. The endocytic index was computed by dividing the area of endocytosis puncta by the total cell area. At least three fields were randomly selected from 5 to 10 different regions across each sample. Moreover, the vesicles were also labeled with primary antibodies against caveolin, clathrin, EEA1, or Rab5 (Cell Signaling Technology, Danvers, MA, USA) and quantified through a similar pipeline. For quantification of internalized BSA-FITC, cells were lysed with radioimmunoprecipitation assay buffer (RIPA) buffer and analyzed by a BMG PolarStar plate reader (BMG Labtech, Ortenberg, Germany). The same lysate was used for protein quantification through Bio-Rad protein assay. For flow cytometry analysis, cells were harvested and FITC fluorescence was analyzed by a BD FACS Canto II cell analyzer (BD, Franklin Lakes, NJ, USA). In some assays, cells were pretreated with cytochalasin D (5 μM) or EIPA (20 μM) for 1 h before BSA/dextran treatments.

### 3.13. Immunofluorescence

Immunofluorescence assay was performed as previously described [28]. In brief, 5000 cells were harvested and seeded on μ-Slide eight well (Ibidi, Martinsried Planegg, Germany). After treatments, cells were washed three times in PBS and fixed in 4% paraformaldehyde. Cells were then blocked with superblock PBS supplemented with 0.3% Triton X-100, and then labeled with primary antibodies (1/200) at 4 °C overnight. Samples were further labeled with fluorescent-conjugated secondary antibodies (Thermo Fisher Scientific, Waltham, MA, USA) and visualized by Zeiss LSM 880 confocal microscope.

### 3.14. Subcutaneous Xenograft

The subcutaneous xenografts were established as previously described [29]. Five million cells were subcutaneous injected into 4–6-week-old athymic nude mice; the mice with no xenograft generation were excluded from further analysis. Xenografts (*IDH1^WT^*, *n* = 2; *IDH1^R132H^*, *n* = 6) were allowed to attain a volume of 300 mm^3^. When the xenografts approached 300 mm^3^, endocytic vesicles were labeled with 20 mg/mL dextran-FITC by intra-tumoral injection at 60 min before the mice were euthanized. Tumors were then harvested for histology analysis [27]. All animal studies were conducted following the principles and procedures outlined in the NIH Guide for the Care and Use of Animals and approved by the Animal Care and Use Committee of the National Institute of Health ethic code (NOB-001).

### 3.15. Electron Microscopy

Cultured cells were examined by scanning and transmission electron microscopes (SEM and TEM). Both samples were fixed in situ by 0.1 M sodium cacodylate buffer containing 4% formaldehyde and 2% glutaraldehyde (v/v, pH 7.4) for 2 h at room temperature. The fixative was replaced with cacodylate buffer and post-fixed in 1% (v/v) osmium tetroxide in the same buffer for 1 h. The cells were then dehydrated in a series of ethanol (35%, 50%, 75%, 95%, and 100%, v/v). For SEM, the cells were immersed in TMS (tetramethylsilane) and allowed to air dry. Subsequently, the samples were sputter-coated with a thin layer of iridium and imaged utilizing a Hitachi S-4500 field emission scanning electron microscope. For TEM, the cells were embedded in an epoxy resin after 100% ethanol dehydration and cured at 55 °C oven for 48 h. Seventy to eighty nanometer-thin sections were made from the cured epoxy resin in a parallel direction to the cell media and mounted on 150 meshed copper grids. The thin sections were stained with 0.5% uranyl acetate (*w*/*v* aqueous solution) and Reynold’s lead citrate. The thin sections were stabilized by carbon coating in a vacuum evaporator, and then they were observed and imaged in the TEM.

### 3.16. Western Blot

Cells were homogenized in RIPA lysis buffer and resolved on NuPAGE Bis-Tris gels. The protein was transferred to PVDF membrane, probed with primary antibody, and visualized through chemiluminescence assay (Thermo Fisher Scientific, Waltham, MA, USA). The primary antibodies used in this study include EEA1 (CST 3288, 1:2,000), Rab5 (CST 3547, 1:2,000), caveolin-1 (CST, Danvers, MA, USA, 3267, 1:2,000), clathrin (CST 4796, 1:2,000), Rab7 (CST 9367, 1:2,000), Rab11 (CST 5589, 1:2,000), Rac1 (CST 2465, 1:2,000), pRac1 (CST 2461, 1:1,000), Cdc42 (CST 2466, 1:2,000), Lysosomal-associated membrane protein 1 (lamp1, Abcam ab25630, 1:2,000; Cambridge, UK), and β-actin (Sigma A5441, 1:2,000; St. Louis, MO, USA). Uncut blotting images are enclosed in Supplementary Figures S5 and S6.

### 3.17. Patient Data Analysis

Lower grade glioma data generated by the TCGA Research Network http://cancergenome.nih.gov/ were downloaded from Firehose (https://gdac.broadinstitute.org/) (using R’s RTCGAToolBox package and build of 08/21/2015. Molecular annotation data were downloaded from cBioPortal (http://www.cbioportal.org/) using R’s cgdsr package. RNASeq counts from 367 IDH-mutant gliomas and 85 IDH-WT gliomas were normalized using variance stabilized transformation, as implemented by R’s DESeq2 package. The principal component analysis was performed on the basis of the expression of the 500 most varied genes between the IDH-mutant and wild type cohorts. 

### 3.18. RNA Interference (RNAi)

Small interfering RNA (siRNA) targeting Rictor and Rac1 were purchased from Integrated DNA Technologies. A silencer-negative control RNA (Thermo Fisher Scientific, Waltham, MA, USA) was used for non-targeted control. The siRNA was transfected with Lipofectamine RNAiMAX (Thermo Fisher Scientific, Waltham, MA, USA) according to the manufacturer’s instructions. The expression of Rac1 was measured by Western blot and real-time PCR. Cells were used for consequent analysis 48 h after transfection. The siRNA oligos used in the present study include: siRac1.1F: 5′-GGU AUU AUC AGG AAA UGU UUU CUT A-3′; siRac1.1R: 5′-UAA GAA AAC AUU UCC UGA UAA UAC CAA-3′; siRac1.2F: 5′-GGU AAA ACU UGC CUA CUG AUC AGT T-3′; siRac1.2R: 5′-AAC UGA UCA GUA GGC AAG UUU UAC CUA-3′; siRictor.1F: 5′-CAA GAU CAC UUG CUA AAA CUU ACT G-3′; siRictor.1R: 5′-CAG UAA GUU UUA GCA AGU GAU CUU GGU-3′; siRictor.2F: 5′-CAU UUU UCC UUG AUA UCA AUG AAG A-3′; siRictor.2R: 5′-UCU UCA UUG AUA UCA AGG AAA AAU GUA-3′.

### 3.19. Seahorse Assay

Twenty thousand cells were seeded on a Seahorse XF cell culture microplate. The energy metabolic phenotype was determined through the Seahorse assay (Agilent, Santa Clara, CA, USA) according to the manufacturer’s protocol. The energy phenotype was expressed by plotting oxygen consumption rate (OCR) and extracellular acidification rate (ECAR). 

### 3.20. BrdU Incorporation Assay

BrdU incorporation assay was performed as previously described [30]. BrdU (Sigma, St. Louis, MO, USA) was added to the media at a concentration of 10 μM. Cells were incubated with BrdU for 3 h in growth condition. Cells were fixed with 4% paraformaldehyde (PFA) for 15 min and permeabilized with 0.3% Triton X-100 in PBS. Cells were treated with 2 *n* HCl for 10 min and neutralized with 0.1 M sodium borate, pH 8.5, for 30 min. Cells were stained with mouse anti-BrdU antibody, 20 μL per sample (BD Biosciences, San Diego, CA, USA), overnight at 4 °C. Cells were labeled with donkey anti-mouse Alexa Fluor 488 immunoglobulin G (IgG, 1:400) and Hoechst 33,342 (1:5000) for 30 min at room temperature and visualized by Zeiss LSM 780 confocal microscope. BrdU-positive rate was calculated and quantified from six random images.

### 3.21. Sphere Formation Assay

BTICs GSC923 and TS603 were harvested and digested into a single-cell suspension. Ten thousand BTICs were seeded into a 6-well plate. Treatment was directly added into the tissue culture media in the plate. Cells were incubated in growth conditions for 2 weeks to allow the formation of stem cell spheres. By the end of the assay, 10 phase-contrast images were randomly taken for each well. The numbers and sizes (diameters) of the BTIC spheres were quantified using ImageJ software.

### 3.22. GST-Pull Down Assay

GST-pull down assay was performed using Active Rac1 Detection Kit (CST, Danvers, MA, USA) to determine the GTP-bounded rac1. In brief, 10 million cells were harvested and lysed on ice. Five hundred total protein was incubated with 20 μg GST-PAK1-PBD and glutathione resin at 4 °C for 1 h. After washing the resin with lysis buffer, the bound protein was eluted by 2x SDS sample buffer supplemented with 200 mM dithiothreitol. The quantity of GTP-bound rac1 was determined by immunoblotting.

### 3.23. Statistical Analysis

Quantitative experiments were performed in triplicate. No randomization or blinding was used in the study. Statistical analyses were performed using GraphPad Prism software (ver. 7.01, GraphPad Software, Inc. La Jolla, CA, USA). All statistical tests were two-sided; the results are presented as mean ± standard error of the mean (SEM). Statistical comparisons were conducted using independent Student’s *t*-test—* *p*-value < 0.05, ** *p*-value < 0.01, *** *p*-value < 0.001. *p*-value < 0.05 was considered as statistically significant. 

## 4. Discussion

**Figure 7:**
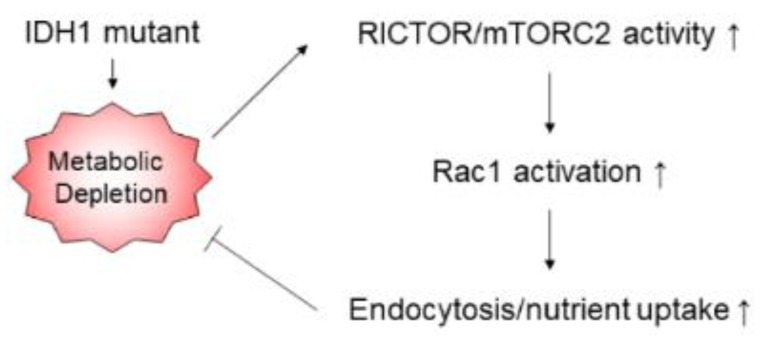
Correlation of IDH1 mutation and cancer aggressiveness. Pathogenic IDH1 mutation depletes cellular metabolism through D-2-hydroxyglutarate (D-2-HG) production, which contributes to an aggressive phenotype in cancer cells. The mTORC2/Rac1 pathway and concomitant cytoskeleton mobilization promote cellular food-seeking, motility, chemotaxis, and endocytosis.

The present study revealed that pathogenic *IDH1* mutation contributes to cancer aggressiveness via prompting cellular food-seeking, motility, chemotaxis, and endocytosis, which resulted from the augmented mTORC2/Rac1 pathway and concomitant cytoskeleton mobilization. Enhanced endocytosis assists in the proliferation of *IDH1*-mutated cells. Targeting Rictor, as well as its downstream molecule Rac1, suppresses *IDH1*-mutated cancer progression with reduced cell motility and endocytosis (Figure 7).

Mutations in *IDH1/2* have been identified in multiple types of human malignancies, such as lower grade glioma, secondary glioblastoma, acute myeloid leukemia, chondrosarcoma, and cholangiocarcinoma [31]. In lower grade glioma, *IDH*-mutated glioma could be subcategorized into astrocytoma (Tumor protein p53 and X-linked helicase II-mutated, TP53/ATRX-mutated, telomerase reverse transcriptase promoter intact, hTERT promoter intact, 1p/19q intact) and oligodendroglioma (TP53/ATRX intact, hTERT promoter-mutated, 1p/19q co-deleted) [1]. Although several recent investigations have revealed the therapeutic vulnerabilities in *IDH*-mutated glioma, they are considered malignant diseases because they are rarely cured and frequently transformed into secondary glioma [18,24,32,33]. Specific and potent therapeutics have long been urged for this type of malignancy.

Our study provides conceptual advances to understand metabolic reprogramming in *IDH1*-mutated cells. Pathogenic mutations in *IDH1* lead to neomorphic changes in the enzyme activity, which allow the enzyme to utilize α-KG, leading to D-2-HG production [5,8,9]. The variant of R132C and R132H are frequently identified in *IDH*-mutated malignancies, and R132C variant catalyzes D-2-HG production more efficiently [34,35]. Several pioneering findings suggested that the changes in *IDH1* catalytic function result in substantial changes in cellular metabolisms, such as the depletion of carbon hydrate metabolites from the Krebs cycle, Warburg-like metabolism, and utilization of alternative metabolites [36]. The concept of metabolic depletion has been counterintuitive for oncogenesis, which demands an adequate metabolite for increased cell proliferation. In the present study, we demonstrated a possible metabolic compensation mechanism for *IDH1*-mutated glioma by elevating mTORC2 and small GTPase activity, which reorganized cytoskeleton and plasma membrane, and facilitated substance uptake via enhanced endocytosis. Transcriptomic analysis showed that several cell movement-related genes were significantly up-regulated in IDH1-mutated glioma cells. For example, *BMP4,* which encodes bone morphogenetic protein 4, was upregulated by 6.47-fold in *IDH1*-mutated cells. Overexpression of BMP4 is related to the invasion and migration of melanoma cells and ovarian cancer cells [32,37,38]. As another example, *CHI3L1*, which encodes chitinase 3-like 1, was up-regulated by 38.20-fold in cells harboring *IDH1* mutants. The expression of *CHI3L1* is related to cellular adhesion, migration, and invasion [39,40]. On the other hand, a sub-population of movement-related genes were down-regulated in *IDH1*-mutated cells. For example, *POSTN*, which encodes periostin, a secreted protein that interferes with cellular migration and epithelial-to-mesenchymal transition, was reduced by -34.33-fold in *IDH1*-mutated cells [41]. As another example, *DPP4*, which encodes dipeptidyl peptidase IV, was reduced by -1.04-fold in *IDH1*-mutated cells. The DPP4-seprase complex facilitates the degradation of the extracellular matrix and cellular invasion into collagenous matrices [42]. Overall, we discovered that the acquisition of cancer-associated *IDH1* mutant is associated with a distinctive transcriptional pattern, which may contribute to the unique food-seeking pattern. 

RNA sequencing data identified that Rictor is selectively up-regulated in *IDH1*-mutated lower grade gliomas. Consistently, we showed up-regulation of Rictor in IDH1 the mutant-transduced model and patient-derived cells, which potentiates the activity of mTORC2. These findings suggested a significant correlation between *IDH1* mutant enzyme and Rictor/mTORC2 mobilization. However, the molecular mechanism of how the *IDH1* mutant enzyme activated mTORC2 activity remains elusive. One possible explanation is that the *IDH1*-distinctive hypermethylation phenotype may enable some of the key regulatory elements in the mTORC2 complex. The accumulation of 2-HG may also play a role in inhibiting α-KG-dependent dioxygenases and enhancing mTORC2 activity through an unknown mechanism [43]. Moreover, the *IDH1*-mutant depletes metabolites from the TCA cycle, which may also activate endocytosis pathways such as mTORC2. More effort is necessary to reveal the link between *IDH1* mutation and the mTOR pathway.

The unexpected finding of the Rictor/Rac1 pathway in the *IDH1*-mutated cell suggested a novel druggable target in this type of malignancy. Our study highlights a possible therapeutic strategy for *IDH1*-mutated cancers by targeting the endocytosis pathway, such as the mTORC2/Rictor/Rac1 axis. It is likely that in the context of the IDH mutant, the Krebs cycle metabolites are depleted in the form of D-2-hydroxyglutarate. The endocytosis pathway is enabled in order to compensate for the loss of metabolites. As a result, rac1, the primary mediator for endocytosis, becomes essential for cancer cell metabolism and could be a valuable drug target. Recent advances in glioma research indicate that *IDH1*-mutated cancer exhibits a vulnerability pattern different from that of their wild type counterparts [18,24,44,45,46]. Through unbiased gene expression analysis, we identified the distinctive activation of molecular pathways that govern cellular motility and shape changes. The alteration in endocytic pathways may facilitate cancer cells to acquire metabolites from the extracellular milieu. Rictor/Rac1, as well as their concomitant downstream effector WAVE1/2, were found to be activated in *IDH1*-mutated cells. Targeting the Rictor/Rac1 pathway could be considered as a plausible therapeutic approach.

Our study provided proof-of-concept evidence that targeting the mTORC2/Rictor/Rac1 pathway could be useful to treat *IDH1*-mutated cancers. However, there are a few limitations to the present study. For example, the chemical structure of the present small molecular inhibitors does not allow effective brain tumor delivery. Additionally, the required dosage of the Rac1 inhibitor is too high for human therapeutic use to avoid potential side effects. Therefore, more efforts may be required to design more effective small molecular inhibitors for better targeting of the Rictor/Rac1 pathway. 

## 5. Conclusions

In summary, this study demonstrated that *IDH1*-mutated cancer cells exhibit increased endocytosis through activation of the mTORC2/Rictor/Rac1 axis. Enhanced endocytosis assists the proliferation of *IDH1*-mutated cells, which indicates that targeting the mTORC2/Rictor/Rac1 pathway could be a new approach to treat this type of malignancy.

## Supplementary Materials

The following are available online at https://www.mdpi.com/2072-6694/12/4/787/s1: Figure S1: The expression of mutant IDH1 in U251 was validated by immunoblotting. Figure S2: Quantification of D-2-HG in U251 cells with doxycycline-induced IDH1-R132H expression. Figure S3: Confocal microscopy showed endosomal markers caveolin, clathrin, EEA1, or Rab5 in BTIC. Figure S4: Western blot analysis showed enhanced mTORC2 downstream pAkt, pPKCα in IDH1-mutated U251 cells as well as BTICs. Figure S5: Western blot images for Figure 3E. Lane 1: U251 IDH1^WT^. Lane 2: U251 IDH1^R132C^. Figure S6: Western blot images for Figure S1A. Lane 1: U251, no transfection (NT) Table S1: List of differentially expressed genes in U251 IDH1^R132H^ compared with U251 IDH1^WT^. Table S2: List of differentially expressed cell movement related genes in U251 IDH1^R132H^ compared with U251 IDH1^WT^. Table S3: List of differentially expressed genes in U251 IDH1^R132C^ compared with U251 IDH1^WT^. Table S4: List of differentially expressed cell movement related genes in U251 IDH1^R132C^ compared with U251 IDH1^WT^.
