# Supplementary material for: mTORC2/Rac1 Pathway Predisposes Cancer Aggressiveness in IDH1-Mutated Glioma
Source: Cancers (Basel). 2020 Mar 26;12(4):787. doi: 10.3390/cancers12040787 (PMC7226122; doi:10.3390/cancers12040787)
Supplement: Supplementary file 1 [file cancers-12-00787-s001.zip › cancers-733430-revised-suppl-3.18/cancers-733430-suppl-revised-3.18.docx]

**Supplementary data information**

Supplementary data include 6 supplementary figures and 4 supplementary tables.

*
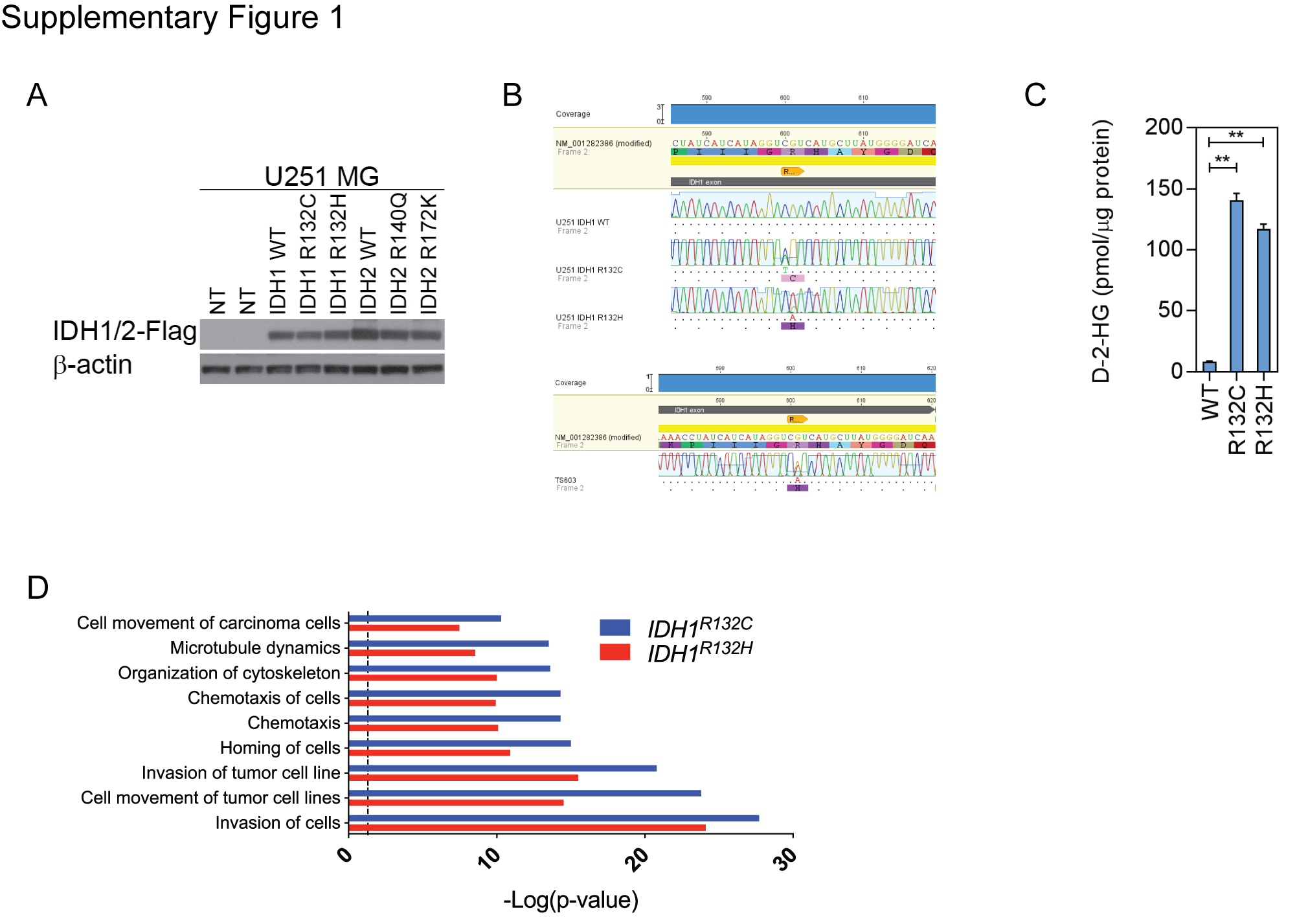
*

***Supplementary Figure 1***

1. The expression of mutant IDH1 in U251 was validated by immunoblotting. IDH1/2 protein with FLAG tag was detected using anti-DDK antibody. β-actin was used as loading control. NT, non-transfected.
2. IDH1 mutation was confirmed by Sanger sequencing in U251 *IDH1^R132C^*, *IDH1^R132H^* and TS603 (*IDH1^R132H^*) BTIC.
3. Cellular D-2-HG level was measured by colorimetric D-2-HG assay kit in U251 IDH1 mutated cells. WT: 8.23 ± 0.72, n=3; R132C: 140 ± 5.59, n=3; R132H, 117 ± 3.94, n=3. *t* test, **, p<0.01. D-2-HG, D-2-Hydroxyglutarate.
4. Pathway enrichment in *IDH1*-mutated cells based on RNA sequencing data. Signaling pathways related to cell motility and invasion are significantly enhanced.


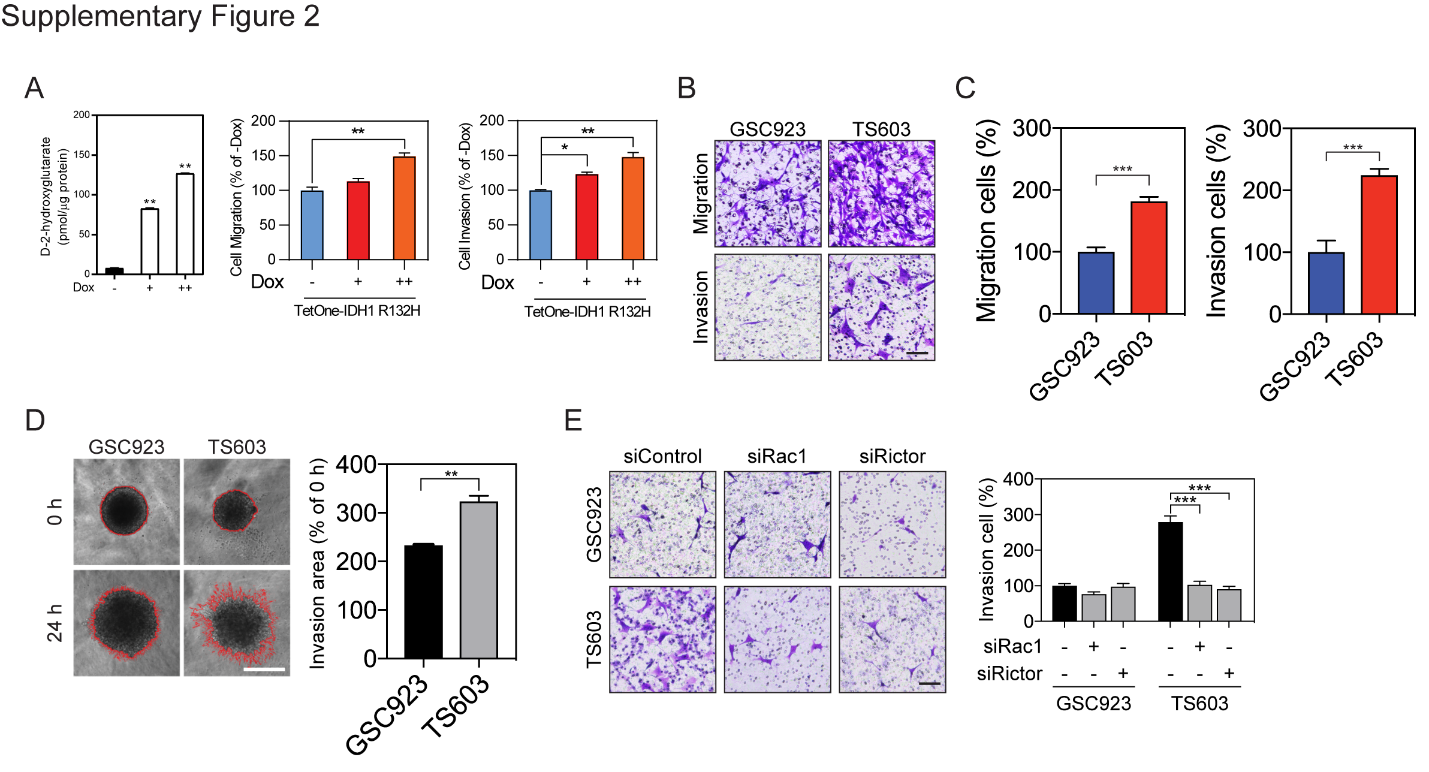


***Supplementary Figure 2***

1. Quantification of D-2-HG in U251 cells with doxycycline-induced IDH1-R132H expression (left panel). Boyden chamber migration and invasion assay showed phenotypic changes in U251 cells with doxycycline-induced IDH1-R132H expression (right panel). This experiment was repeated three times. D-2-HG: -DOX, 7.89 ± 0.21, n=3; +DOX, 82.80 ± 0.58, n=3; ++DOX, 126.8 ± 0.45, n=3. Cell Migration: -DOX, 100 ± 5.00, n=3; +DOX, 113.4 ± 3.75, n=3; ++DOX, 149.5 ± 4.40, n=3. Cell Invasion: -DOX, 100 ± 1.26, n=3; +DOX, 123.4 ± 2.56, n=3; ++DOX, 148.1 ± 6.35, n=3. *t* test, *p<0.05, **p<0.01. (-, no Doxycycline; + 10ng/mL Doxycycline; ++ 100ng/mL Doxycycline)
2. Boyden chamber assay showed cellular migration and invasion in BTICs. TS603 (*IDH1^R132H^*) exhibited stronger cell motility and invasion. This experiment was repeated three times. Bar = 50 μm.
3. Quantification of Boyden chamber assay shown in Supplementary Figure 1B. GSC923 was used as 100%. Migration: GSC923, 100 ± 7.22, n=6; TS603, 181 ± 7.58, n=6. Invasion: GSC923, 100 ± 18.7, n=6; TS603, 225 ± 10.2, n=6. *t* test, ***p<0.001.
4. Cell invasion was measured by 3D invasion assay in BTICs. Bar = 100 μm (left panel). Quantification of invasion area, the area of the sphere in 0 hr was used as 100% (right panel). This experiment was repeated three times. GSC923: 233 ± 1.53, n=3; TS603: 324 ± 11.9, n=3. *t* test, **, p<0.01.
5. Cell invasion was measured using Boyden chamber assay in BTIC after Rac1 and Rictor knockdown, siRac1-1 and siRictor-1 were used. Bar = 50 μm (left panel). Quantification of invasion cell was shown (right panel), GSC923 siControl group was used as 100%. This experiment was repeated three times. GSC923: siCont, 100 ± 5.78, n=6; siRac1, 76.8 ± 5.88, n=6; siRictor, 97.3 ± 9.36, n=6. TS603: siCont, 280 ± 16.6, n=6; siRac1, 103 ± 10.1, n=6; siRictor, 90.5 ± 8.22, n=6. *t* test, ***, p<0.001.


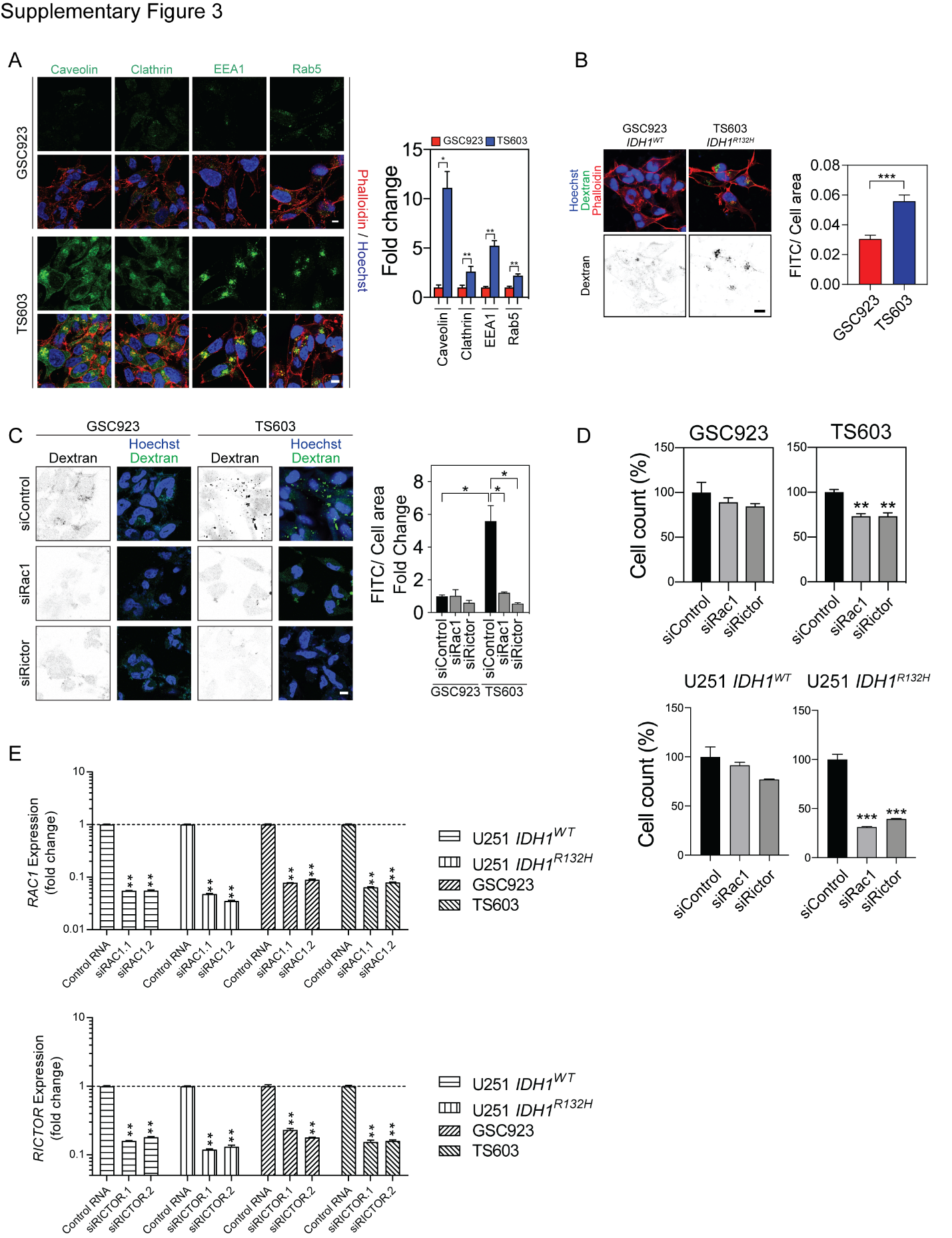


***Supplementary Figure 3***

1. Confocal microscopy showed endosomal markers Caveolin, Clathrin, EEA1 or Rab5 in BTIC. Endosomal markers were labeled in green. Cell boundary was highlighted with phalloidin (red), Cell nuclei were labeled with Hoechst 33342 (blue). Bar = 10 µm. Quantification of endosomal markers by averaging the area of the vesicle to the total area of the cells (right panel). GSC923 was used as control. Caveolin: GSC923, 1.00 ± 0.265, n=3; TS603, 11.1 ± 1.65, n=3. Clathrin: GSC923, 1.00 ± 0.243, n=3; TS603, 2.63 ± 0.530, n=3. EEA1: GSC923, 1.00 ± 0.119, n=3; TS603, 5.24 ± 0.500, n=3. Rab5: GSC923, 1.00 ± 0.142, n=3; TS603, 2.21 ± 0.175, n=3. *t* test, *, p<0.05, **, p<0.01.
2. Dextran uptake was measured in BTIC. Dextran was labeled by FITC (green), cell boundary was highlighted with phalloidin (red), cell nucleus was labeled with Hoechst 33342 (blue). The dextran signal was highlighted with binary images. Bar = 10 μm. (left panel). Quantification of dextran uptake by averaging the area of dextran signal to the total cell area (right panel). GSC923: 0.031 ± 0.002, n=42; TS603: 0.056 ± 0.004, n=42. *t* test, ***, P<0.001.
3. Dextran uptake was measured in BTIC after Rac1 and Rictor knockdown. Dextran was labeled by FITC (green), cell nucleus was labeled with Hoechst 33342 (blue). The dextran signal was highlighted with binary images, siRac1-1 and siRictor-1 were used. Bar = 10 μm. (left panel). Quantification of dextran uptake by averaging the area of dextran signal to total cell area (right panel). GSC923: siCont, 1.00 ± 0.073, n=3; siRac1, 1.04 ± 0.373, n=3; siRictor, 0.62 ± 0.128, n=3. TS603: siCont, 5.60 ± 0.939, n=3; siRac1, 1.23 ± 0.035, n=3; siRictor, 0.562 ± 0.038, n=3. *t* test, *, p<0.05.
4. Cell viability was measured by cell counting in BTIC after Rac1 and Rictor knockdown, siRac1-1 and siRictor-1 were used. The siControl group was used as 100%. This experiment was repeated three times. GSC923: siControl, 100 ± 11.3, n=3; siRac1, 89.1 ± 5.01, n=3; siRictor, 84.6 ± 3.10, n=3. TS603: siControl, 100 ± 2.99, n=3; siRac1, 73.1 ± 2.99, n=3; siRictor, 73.1 ± 3.95, n=3. U251 *IDH1^WT^*, siControl, 100 ± 10.1, n=3; siRac1, 91.2 ± 3.06, n=3; siRictor, 76.9 ± 0.549, n=3. U251 *IDH1^R132H^*, siControl, 100 ± 5.28, n=3; siRac1, 31.0 ± 0.633, n=3; siRictor, 39.4 ± 0.645, n=3. *t* test, **, p<0.01. ***, p<0.001.
5. The gene expressions of *RAC1* and *RICTOR* were quantified through real-time PCR. Data was normalized to Control RNA in transfected cells. This experiment was repeated three times. For *RAC1*, U251 IDH1 WT: Control RNA, 1.000 ± 0 .014, n=3; siRAC1.1, 0.055 ± 0.001, n=3; siRAC1.2, 0.055 ± 0.001, n=3. U251 IDH1 R132H: Control RNA, 1.000 ± 0.013, n=3; siRAC1.1 0.048 ± 0.001 n=3; siRAC1.2 0.035 ± 0.001, n=3. GSC923: Control RNA, 1.001 ± 0.022, n=3; siRAC1.1 0.078 ± 0.001 n=3; siRAC1.2 0.089 ± 0.003, n=3. TS603: Control RNA, 1.001 ± 0.026, n=3; siRAC1.1 0.064 ± 0.002 n=3; siRAC1.2 0.079 ± 0.001, n=3. For RICTOR, U251 IDH1 WT: Control RNA, 1.000 ± 0.018, n=3; siRICTOR.1.1, 0.160 ± 0.002, n=3; siRICTOR.2, 0.180 ± 0.005, n=3. U251 IDH1 R132H: Control RNA, 1.000 ± 0.013, n=3; siRICTOR.1.1, 0.119 ± 0.003, n=3; siRICTOR.2, 0.131 ± 0.007, n=3. GSC923: Control RNA, 1.002 ± 0.050, n=3; siRICTOR.1.1, 0.230 ± 0.011, n=3; siRICTOR.2, 0.180 ± 0.002, n=3. TS603: Control RNA, 1.001 ± 0.031, n=3; siRICTOR.1.1, 0.154 ± 0.009, n=3; siRICTOR.2, 0.160 ± 0.005, n=3. t-test, **p<0.01.





***Supplementary Figure 4***

1. Western blot analysis showed enhanced mTORC2 downstream pAkt, pPKCα in *IDH1*-mutated U251 cells as well as BTICs. Rac1 phosphorylation was found enhanced in *IDH1*-mutated cells. The mTORC1 downstream p-p70S6K or p4EBP1 were not altered by *IDH1* mutation.
2. mTORC2 downstream pAkt and pPKCa was measured by Western blot in U251 *IDH1^WT^* and *IDH1^R132H^*, as well as BTIC. This experiment was repeated three times. U251 *IDH1^WT^* and GSC923 were used as control. U251 pAkt/Akt: *IDH1^WT^*, 1.00 ± 0.06, n=3; *IDH1^R132H^*, 2.43 ± 0.28, n=3. U251 pPKCα/PKCα: *IDH1^WT^*, 1.00 ± 0.105, n=3; *IDH1^R132H^*, 1.59 ± 0.184, n=3. BTIC pAkt/Akt: GSC923, 1.00 ± 0.145, n=3; TS603, 2.40 ± 0.294, n=3. BTIC pPKCα/PKCα: GSC923, 1.00 ± 0.202, n=3; TS603, 1.94 ± 0.067, n=3. *t* test, *, p<0.05, **, p<0.01.
3. mTORC2 downstream pAkt and pPKCα was measured by Western blot in U251 *IDH1^R132H^* and TS603 BTIC after Rictor knockdown. This experiment was repeated three times. siControl group for each cell line was used as control. TS603 pAkt/Akt: siCont, 1.00 ± 0, n=3; siRictor.1, 0.30 ± 0.049, n=3; siRictor.2, 0.26 ± 0.020, n=3. TS603 pPKCα/PKCα: siCont, 1.00 ± 0, n=3; siRictor.1, 0.28 ± 0.088, n=3; siRictor.2, 0.24 ± 0.097, n=3. U251 *IDH1^R132H^* pAkt/Akt: siCont, 1.00 ± 0, n=3; siRictor.1, 0.30 ± 0.004, n=3; siRictor.2, 0.30 ± 0.060, n=3. U251 *IDH1^R132H^* pPKCα/PKCα: siCont, 1.00 ± 0, n=3; siRictor.1, 0.25 ± 0.035, n=3; siRictor.2, 0.47 ± 0.140, n=3. *t* test, *, p<0.05, **, p<0.01, ***, p<0.001.





***Supplementary Figure 5***

1. Western blot images for Figure 3E. Lane 1: U251 *IDH1^WT^*. Lane 2: U251 *IDH1^R132C^*. Lane 3: U251 *IDH1^R132H^*.
2. Western blot images for Figure 3H. Lane 1: U251 *IDH1^R132H^* with wild-type Rac1 expression. Lane 2: U251 *IDH1^R132H^* with dominant-negative recombinant of Rac1 (T17N) expression. Lane 3: U251 *IDH1^R132H^* with dominant-active recombinant of Rac1 (Q61L) expression.
3. Western blot images for Figure 4C, mTORC1 signaling pathway. Lane 1: TS603 with negative control siRNA. Lane 2: TS603 with siRictor-1. Lane 3: TS603 with siRictor-2.
4. Western Blot images for Figure 4C, mTORC1 signaling pathway. Lane 1: U251 *IDH1^R132H^* with negative control siRNA. Lane 2: U251 *IDH1^R132H^* with siRictor-1. Lane 3: U251 *IDH1^R132H^* with siRictor-2.
5. Western blot images for Figure 4C, mTORC2 signaling pathway. Lane 1: TS603 with negative control siRNA. Lane 2: TS603 with siRictor-1. Lane 3: TS603 with siRictor-2.
6. Western Blot images for Figure 4C, mTORC2 signaling pathway. Lane 1: U251 *IDH1^R132H^* with negative control siRNA. Lane 2: U251 *IDH1^R132H^* with siRictor-1. Lane 3: U251 *IDH1^R132H^* with siRictor-2.


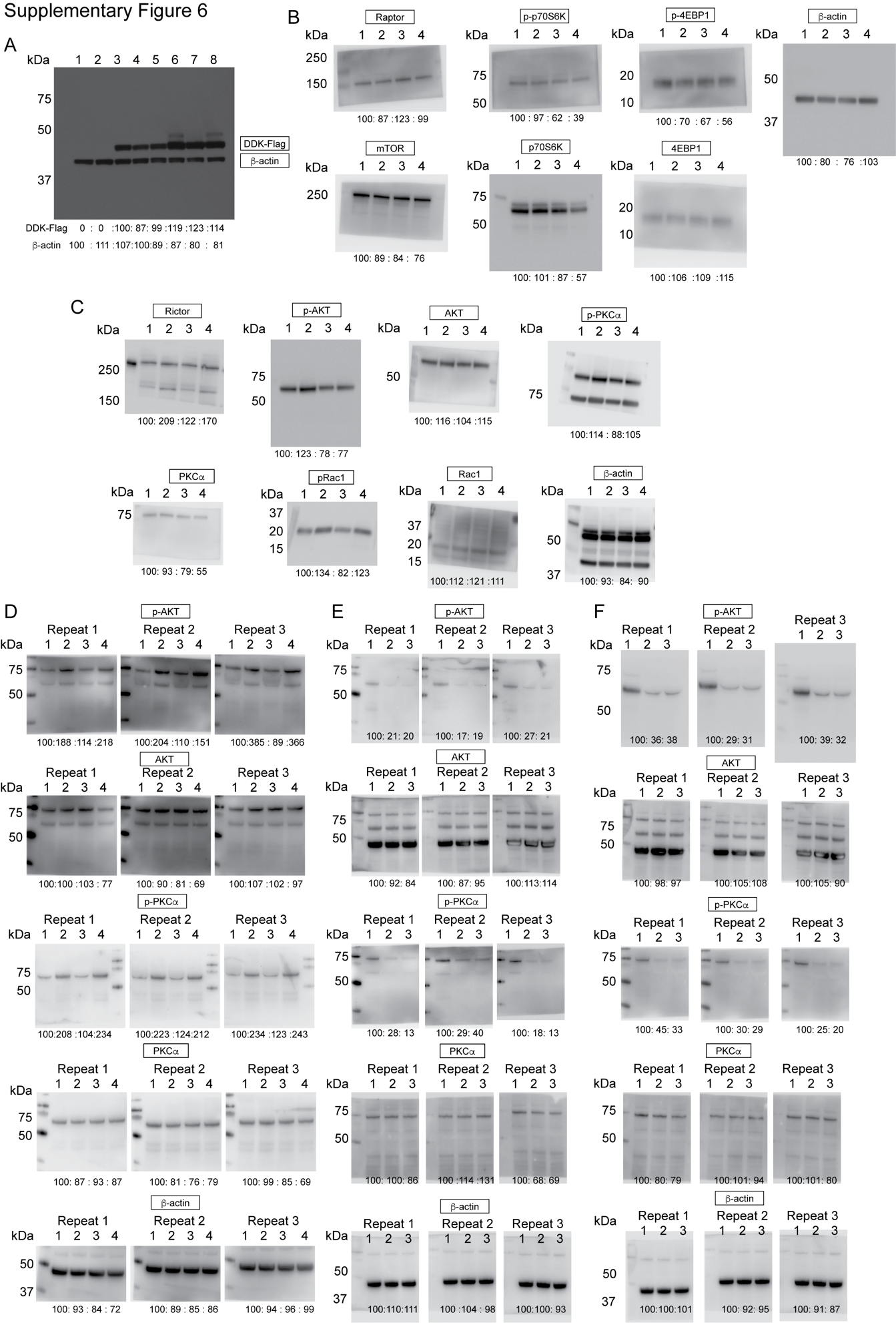


***Supplementary Figure 6***

1. Western blot images for Supplementary Figure 1A. Lane 1: U251, no transfection (NT). Lane 2: NT. Lane 3: U251 *IDH1^WT^*. Lane 4: U251 *IDH1^R132C^*. Lane 5: U251 *IDH1^R132H^*. Lane 6: U251 *IDH2^WT^*. Lane 7: U251 *IDH2^R140Q^*. Lane 8: U251 *IDH2^R172K^*.
2. Western blot images for Supplementary Figure 4A, mTORC1 signaling pathway. Lane 1: U251 *IDH1^WT^*. Lane 2: U251 *IDH1^R132H^*. Lane 3: GSC923. Lane 4: TS603.
3. Western blot images for Supplementary Figure 4A, mTORC2 signaling pathway. Lane 1: U251 *IDH1^WT^*. Lane 2: U251 *IDH1^R132H^*. Lane 3: GSC923. Lane 4: TS603.
4. Western blot images for Supplementary Figure 4B. Lane 1: U251 *IDH1^WT^*. Lane 2: U251 *IDH1^R132H^*. Lane 3: GSC923. Lane 4: TS603.
5. Western blot images for Supplementary Figure 4C, left panel. Lane 1: TS603 with negative control siRNA. Lane 2: TS603 with siRictor1. Lane 3: TS603 with siRictor2.
6. Western blot for Supplementary Figure 4C, right panel. Lane 1: U251 *IDH1^R132H^* with negative control siRNA. Lane 2: U251 *IDH1^R132H^* with siRictor1. Lane 3: U251 *IDH1^R132H^* with siRictor2.

***Supplementary Table 1***

List of differentially expressed genes in U251 *IDH1^R132H^* compared with U251 *IDH1^WT^*

***Supplementary Table 2***

List of differentially expressed cell movement related genes in U251 *IDH1^R132H^* compared with U251 *IDH1^WT^*

***Supplementary Table 3***

List of differentially expressed genes in U251 *IDH1^R132C^* compared with U251 *IDH1^WT^*

***Supplementary Table 4***

List of differentially expressed cell movement related genes in U251 *IDH1^R132C^* compared with U251 *IDH1^WT^*
