# Supplementary material for: mTORC2/Rac1 Pathway Predisposes Cancer Aggressiveness in IDH1-Mutated Glioma
Source: Cancers (Basel). 2020 Mar 26;12(4):787. doi: 10.3390/cancers12040787 (PMC7226122; doi:10.3390/cancers12040787)
Supplement: Supplementary file 1 [file cancers-12-00787-s001.zip › cancers-733430-revised-suppl-3.18/Supplementary Table 1.pdf]

Supplementary Table 1. List of differentially expressed genes in U251 IDH1 R132C compared with U251 IDH1 WT

Gene Symbol	Entrez Gene Name	Fold Change	FDR
A2M	alpha-2-macroglobulin	1.621	3.04E-19
ABCA1	ATP binding cassette subfamily A member 1	1.726	3.22E-27
ABCA4	ATP binding cassette subfamily A member 4	3.462	0.000074
ABCB11	ATP binding cassette subfamily B member 11	-4.134	0.000181
ABHD8	abhydrolase domain containing 8	-1.612	0.0000783
ABI3BP	ABI family member 3 binding protein	-2.057	1.87E-43
AC0030921		20.264	1.2E-17
AC0051501		-37.495	1.53E-64
AC0091206		3.231	0.0000404
AC092675.1		2.126	0.00122
AC0937871		-2.012	0.000000462
ACKR3	atypical chemokine receptor 3	6.536	5.38E-09
ACP6	acid phosphatase 6, lysophosphatidic	-1.788	1.27E-15
ACSL5	acyl-CoA synthetase long chain family member 5	4.655	1.02E-17
ACSS3	acyl-CoA synthetase short chain family member 3	-1.608	0.0000237
ACTA2	actin alpha 2, smooth muscle	1.674	7.62E-10
ACTG1	actin gamma 1	-1.573	1.34E-10
ACTG2	actin gamma 2, smooth muscle	-7.099	5.8E-12
ADAMTS12	ADAM metallopeptidase with thrombospondin type 1 motif 12	2.154	2.45E-21
ADAMTS14	ADAM metallopeptidase with thrombospondin type 1 motif 14	4.551	0.000000828
ADAMTS2	ADAM metallopeptidase with thrombospondin type 1 motif 2	21.161	1.09E-18
ADGRL2	adhesion G protein-coupled receptor L2	3.343	0.000104
ADORA2B	adenosine A2b receptor	1.569	4.84E-19
ADRA1B	adrenoceptor alpha 1B	-1.635	4.3E-09
ADRA1D	adrenoceptor alpha 1D	-3.074	6.95E-27
ADRB2	adrenoceptor beta 2	2.406	0.0000882
AFAP1L2	actin filament associated protein 1 like 2	3.997	2.52E-23
AFF2	AF4/FMR2 family member 2	-2.446	0.000444
AGMAT	agmatinase	-11.047	0.0000023
AGT	angiotensinogen	1.879	0.00902
AGTRAP	angiotensin II receptor associated protein	2.086	7.32E-20
AHNAK	AHNAK nucleoprotein	1.722	6.65E-19
AHNAK2	AHNAK nucleoprotein 2	1.919	3.61E-26
AJAP1	adherens junctions associated protein 1	-3.059	3.31E-09
AJUBA	ajuba LIM protein	-1.552	1.95E-12

AK4	adenylate kinase 4	-1.777	6.69E-16
AK5	adenylate kinase 5	-3.429	3.38E-44
AKAP6	A-kinase anchoring protein 6	-2.435	1.63E-39
AKR1C1/AKR1C2	aldo-keto reductase family 1 member C2	2.292	2.82E-14
AKR1C3	aldo-keto reductase family 1 member C3	1.804	6.08E-10
ALDH1A3	aldehyde dehydrogenase 1 family member A3	-1.817	1.01E-41
ALK	ALK receptor tyrosine kinase	-3.854	2.99E-16
ALKBH7	alkB homolog 7	-1.792	0.0000143
ALX4	ALX homeobox 4	7.554	1.11E-19
AMH	anti-Mullerian hormone	-2.013	0.000222
AMOT	angiomotin	-1.603	1.86E-15
AMOTL2	angiomotin like 2	-2.205	5.37E-42
ANGPTL4	angiopoietin like 4	7.751	2.5E-24
ANK1	ankyrin 1	-4.559	1.11E-39
ANKRD1	ankyrin repeat domain 1	-2.487	1.34E-30
ANKS1B	ankyrin repeat and sterile alpha motif domain containing 1B	-4.897	2.4E-23
ANO1	anoctamin 1	-2.805	0.000262
ANTXR2	ANTXR cell adhesion molecule 2	1.541	3.04E-14
ANXA6	annexin A6	-1.626	4.79E-12
AOX1	aldehyde oxidase 1	2.013	3.8E-13
AP3B2	adaptor related protein complex 3 subunit beta 2	2.06	0.0000113
APCDD1	APC down-regulated 1	-1.647	0.00000618
APCDD1L	APC down-regulated 1 like	2.379	3.65E-54
APLN	apelin	-1.833	0.00000355
APOL1	apolipoprotein L1	2.137	3.67E-23
APOL6	apolipoprotein L6	1.974	9.21E-27
ARG2	arginase 2	2.331	7.75E-10
ARHGAP24	Rho GTPase activating protein 24	13.636	1.6E-19
ARHGAP25	Rho GTPase activating protein 25	2.342	0.000000891
ARHGAP33	Rho GTPase activating protein 33	-1.702	2.58E-12
ARHGAP36	Rho GTPase activating protein 36	-5.307	3.92E-09
ARHGAP42	Rho GTPase activating protein 42	1.623	0.000000462
ARHGAP45	Rho GTPase activating protein 45	-5.517	1.58E-13
ARHGDIA	Rho GDP dissociation inhibitor alpha	-1.633	0.000000307
ARMH4	armadillo like helical domain containing 4	1.808	0.000000212
ARNT2	aryl hydrocarbon receptor nuclear translocator 2	-1.976	5.65E-34
ARRB1	arrestin beta 1	1.832	0.0000138

ARRDC4	arrestin domain containing 4	-1.531	8.8E-15
ARSL	arylsulfatase L	-1.741	0.00149
ASIC1	acid sensing ion channel subunit 1	1.862	3.01E-09
ASIC3	acid sensing ion channel subunit 3	2.078	0.000000181
ATAD3A	ATPase family AAA domain containing 3A	-1.758	0.0000012
ATOH8	atonal bHLH transcription factor 8	-2.78	0.000000518
ATP10A	ATPase phospholipid transporting 10A (putative)	2.415	3.23E-11
ATP11A	ATPase phospholipid transporting 11A	2.009	1.41E-30
ATP2A1-AS1	ATP2A1 antisense RNA 1	-1.889	0.00135
ATP2B1	ATPase plasma membrane Ca2+ transporting 1	1.709	1.65E-22
ATP6AP1L	ATPase H+ transporting accessory protein 1 like	1.775	0.000496
ATP6V1C2	ATPase H+ transporting V1 subunit C2	-2.044	0.00000826
AZGP1	alpha-2-glycoprotein 1, zinc-binding	-6.456	9.34E-114
B3GALT5	beta-1,3-galactosyltransferase 5	3.82	7.14E-12
B4GALNT3	beta-1,4-N-acetyl-galactosaminyltransferase 3	-2.084	2.19E-25
BACE2	beta-secretase 2	1.634	1.31E-24
BASP1	brain abundant membrane attached signal protein 1	16.922	1.01E-84
BBOX1-AS1	BBOX1 antisense RNA 1	3.404	0.000141
BCAR3	BCAR3 adaptor protein, NSP family member	1.588	1.94E-18
BCHE	butyrylcholinesterase	2.107	0.00165
BCL2L11	BCL2 like 11	2.028	1.8E-24
BEND4	BEN domain containing 4	2.963	0.0000687
BEX1	brain expressed X-linked 1	-3.179	0.0021
BHLHE40	basic helix-loop-helix family member e40	1.776	1.65E-29
BHLHE41	basic helix-loop-helix family member e41	2.669	1.57E-51
BIRC3	baculoviral IAP repeat containing 3	1.93	7.93E-23
BMF	Bcl2 modifying factor	2.176	0.00111
BMP4	bone morphogenetic protein 4	6.467	0.00000848
BOC	BOC cell adhesion associated, oncogene regulated	1.806	4.05E-19
BRINP1	BMP/retinoic acid inducible neural specific 1	50.914	1.43E-54
BTBD11	BTB domain containing 11	9.274	3.47E-17
BTBD19	BTB domain containing 19	1.765	1.52E-09
BTF3L4P4	basic transcription factor 3 like 4 pseudogene 4	156.937	2.81E-11
BTN3A1	butyrophilin subfamily 3 member A1	1.917	1.49E-14
BTN3A3	butyrophilin subfamily 3 member A3	1.88	5.72E-10
BTNL8	butyrophilin like 8	3.931	0.000149
C10orf90	chromosome 10 open reading frame 90	2.224	2.74E-10

C11orf45	chromosome 11 open reading frame 45	1.78	0.0000054
C11orf74	chromosome 11 open reading frame 74	1.698	0.000000019
C11orf87	chromosome 11 open reading frame 87	-14.963	2.97E-08
C16orf74	chromosome 16 open reading frame 74	1.771	0.00000196
C1R	complement C1r	2.525	2.29E-20
C1RL	complement C1r subcomponent like	2.17	1.01E-11
C1S	complement C1s	4.611	2.35E-43
C2CD2	C2 calcium dependent domain containing 2	1.605	9.15E-16
C3	complement C3	7.404	7.74E-28
C3AR1	complement C3a receptor 1	2.566	0.000764
C3orf70	chromosome 3 open reading frame 70	2.651	0.00123
C4orf19	chromosome 4 open reading frame 19	2.394	1.07E-21
C5orf46	chromosome 5 open reading frame 46	-4.638	9.4E-102
C8orf82	chromosome 8 open reading frame 82	-1.663	0.0000541
CA11	carbonic anhydrase 11	1.903	0.000000957
CA8	carbonic anhydrase 8	4.731	0.0000324
CA9	carbonic anhydrase 9	-2.025	4.18E-24
CACNA2D3	calcium voltage-gated channel auxiliary subunit alpha2delta 3	2.432	0.0000136
CACNG4	calcium voltage-gated channel auxiliary subunit gamma 4	1.879	2.96E-13
CAMK2N1	calcium/calmodulin dependent protein kinase II inhibitor 1	2.109	8.27E-19
CAND2	cullin associated and neddylation dissociated 2 (putative)	-8.848	1.25E-16
CAPS	calcyphosine	-2.156	2.12E-09
CARD10	caspase recruitment domain family member 10	-1.741	9.84E-08
CARD6	caspase recruitment domain family member 6	1.681	3.58E-10
CASP1	caspase 1	1.876	0.00000248
CASP4	caspase 4	1.593	4.23E-15
CAVIN3	caveolae associated protein 3	-12.059	0.00000995
CCDC102B	coiled-coil domain containing 102B	-1.831	0.000154
CCDC106	coiled-coil domain containing 106	-2.204	2.96E-12
CCDC144A	coiled-coil domain containing 144A	-2.307	0.00000217
CCDC148	coiled-coil domain containing 148	-3.249	0.000638
CCDC184	coiled-coil domain containing 184	-2.891	1.24E-12
CCDC189	coiled-coil domain containing 189	-1.625	0.00000126
CCDC61	coiled-coil domain containing 61	-1.588	0.00000185
CCDC80	coiled-coil domain containing 80	2.013	4.83E-50
CCN2	cellular communication network factor 2	-1.86	6.31E-54
CD180	CD180 molecule	4.372	8.6E-76

CD200	CD200 molecule	-2.28	3.28E-11
CD274	CD274 molecule	-1.688	3.29E-17
CD300C	CD300c molecule	30.473	0.000000253
CD33	CD33 molecule	30.807	1.93E-174
CD55	CD55 molecule (Cromer blood group)	3.675	1.84E-13
CD82	CD82 molecule	2.865	2.64E-76
CD93	CD93 molecule	3.717	0.000000976
CDH6	cadherin 6	-5.532	1.28E-81
CDKN1A	cyclin dependent kinase inhibitor 1A	1.706	1.94E-17
CDON	cell adhesion associated, oncogene regulated	1.975	1.99E-38
CDT1	chromatin licensing and DNA replication factor 1	-2.267	3.47E-10
CDYL2	chromodomain Y like 2	3.583	2.68E-23
CECR2	CECR2 histone acetyl-lysine reader	17.05	2.44E-10
CELSR2	cadherin EGF LAG seven-pass G-type receptor 2	-1.586	1.36E-13
CEMIP	cell migration inducing hyaluronidase 1	2.334	1.54E-20
CEP131	centrosomal protein 131	-1.838	0.00000374
CERS4	ceramide synthase 4	-1.63	0.0000506
CES1P1	carboxylesterase 1 pseudogene 1	39.234	8.34E-13
CES1P2	carboxylesterase 1 pseudogene 2	78.379	2.58E-24
CFAP45	cilia and flagella associated protein 45	1.733	0.000537
CFAP69	cilia and flagella associated protein 69	2.118	0.00232
CFI	complement factor I	1.574	9.7E-17
CGA	glycoprotein hormones, alpha polypeptide	-3.603	8.64E-08
CGNL1	cingulin like 1	-1.677	2.31E-26
CHAF1A	chromatin assembly factor 1 subunit A	-1.542	4.83E-16
CHFR	checkpoint with forkhead and ring finger domains	-4.147	4.13E-36
CHI3L1	chitinase 3 like 1	38.204	7.44E-269
CHI3L2	chitinase 3 like 2	5.415	2.48E-35
CHRD	chordin	-2.433	0.00115
CHST11	carbohydrate sulfotransferase 11	2.177	4.32E-34
CHST15	carbohydrate sulfotransferase 15	11.452	2.92E-107
CHSY3	chondroitin sulfate synthase 3	-3.845	8.13E-13
CIART	circadian associated repressor of transcription	2.351	3.75E-17
CIB2	calcium and integrin binding family member 2	-1.641	0.000271
CKB	creatine kinase B	-3.036	3.53E-23
CLCA2	chloride channel accessory 2	3.86	0.0000115
CLCN4	chloride voltage-gated channel 4	-1.667	1.11E-08

CLDN1	claudin 1	2.003	6.6E-11
CLDN12	claudin 12	1.691	1.84E-19
CLU	clusterin	2.442	1.98E-35
CMTM8	CKLF like MARVEL transmembrane domain containing 8	-1.817	2.23E-11
CNIH2	cornichon family AMPA receptor auxiliary protein 2	-4.115	7.32E-08
CNR1	cannabinoid receptor 1	-2.776	3.45E-65
CNTNAP2	contactin associated protein like 2	6.052	3.32E-31
COBL	cordon-bleu WH2 repeat protein	8.708	1.22E-09
COL11A1	collagen type XI alpha 1 chain	-1.807	5.44E-32
COL17A1	collagen type XVII alpha 1 chain	3.841	3.96E-94
COL4A4	collagen type IV alpha 4 chain	4.124	0.000148
COL8A1	collagen type VIII alpha 1 chain	-1.549	8.85E-09
COL9A2	collagen type IX alpha 2 chain	-2.299	1.63E-11
COL9A3	collagen type IX alpha 3 chain	-10.911	1.47E-32
COLEC12	collectin subfamily member 12	4.862	0.00000105
CORO1A	coronin 1A	-1.667	0.000215
CORO1B	coronin 1B	-1.567	0.00000026
CORO2B	coronin 2B	-1.809	5.52E-15
CP	ceruloplasmin	2.971	3.75E-86
CPA6	carboxypeptidase A6	-1.663	2.23E-10
CPEB1	cytoplasmic polyadenylation element binding protein 1	2.063	1.19E-08
CPM	carboxypeptidase M	-8.602	6.04E-10
CPT1C	carnitine palmitoyltransferase 1C	-2.111	1.53E-15
CRACD	capping protein inhibiting regulator of actin dynamics	-3.27	1.67E-24
CRISPLD1	cysteine rich secretory protein LCCL domain containing 1	1.768	2.79E-08
CRLF1	cytokine receptor like factor 1	3.959	6.43E-08
CRYAB	crystallin alpha B	-5.072	3.67E-121
CSAG1	chondrosarcoma associated gene 1	2.614	0.00125
CSF1	colony stimulating factor 1	1.716	1.92E-22
CSF2	colony stimulating factor 2	2.847	0.000000504
CTA_384D836		-2.014	0.000000307
CTBS	chitobiase	1.699	2.27E-12
CTD_2017D111		2.075	4.38E-08
CTD_2286N82		3.611	0.0000443
CTNNA2	catenin alpha 2	-3.667	2.02E-42
CTNND2	catenin delta 2	-1.703	1.61E-10
CTSH	cathepsin H	1.854	3.22E-24

CTSS	cathepsin S	3.604	5.28E-34
CTSV	cathepsin V	1.922	1.15E-11
CTSZ	cathepsin Z	-1.888	1.34E-17
CTU2	cytosolic thiouridylase subunit 2	-1.646	0.0000385
CXADR	CXADR Ig-like cell adhesion molecule	2.294	1.04E-29
CXCL12	C-X-C motif chemokine ligand 12	5.381	1.91E-28
CYB5R2	cytochrome b5 reductase 2	-1.939	1.26E-13
CYBRD1	cytochrome b reductase 1	1.818	1.69E-34
CYP27C1	cytochrome P450 family 27 subfamily C member 1	2.191	5.88E-10
CYP46A1	cytochrome P450 family 46 subfamily A member 1	-2.366	1.16E-09
CYP51A1	cytochrome P450 family 51 subfamily A member 1	-2.098	0.0000614
CYTL1	cytokine like 1	-4.171	2.55E-08
DAB1	DAB adaptor protein 1	-4.372	5.76E-09
DAGLA	diacylglycerol lipase alpha	-1.594	0.0000149
DBP	D-box binding PAR bZIP transcription factor	1.698	0.0000081
DCAF15	DDB1 and CUL4 associated factor 15	-1.544	0.000000207
DCLK1	doublecortin like kinase 1	1.64	4.03E-11
DENND2C	DENN domain containing 2C	1.678	1.55E-08
DENND2D	DENN domain containing 2D	-4.852	0.0000486
DGAT2	diacylglycerol O-acyltransferase 2	1.798	9.1E-10
DGCR5	DiGeorge syndrome critical region gene 5	1.899	0.000824
DGKG	diacylglycerol kinase gamma	1.576	2.96E-12
DGKI	diacylglycerol kinase iota	1.675	5.26E-10
DHRS2	dehydrogenase/reductase 2	2.012	0.0000175
DHRS9	dehydrogenase/reductase 9	-4.714	1.85E-13
DIO2	iodothyronine deiodinase 2	-4.564	6.28E-67
DIPK1A	divergent protein kinase domain 1A	1.659	5.8E-13
DIPK2A	divergent protein kinase domain 2A	1.601	6.6E-09
DIRAS1	DIRAS family GTPase 1	-5.067	2.22E-21
DIRAS3	DIRAS family GTPase 3	-1.552	4.68E-12
DISP1	dispatched RND transporter family member 1	1.752	0.000000564
DLC1	DLC1 Rho GTPase activating protein	2.1	1.49E-21
DLL1	delta like canonical Notch ligand 1	2.146	0.0000406
DMBX1	diencephalon/mesencephalon homeobox 1	3.239	3.88E-25
DMKN	dermokine	6.658	0.000121
DNER	delta/notch like EGF repeat containing	1.667	7.58E-26
DNM1	dynamin 1	1.656	1.05E-20

DNM3	dynamin 3	-1.738	6.95E-12
DOCK3	dedicator of cytokinesis 3	2.056	3.13E-13
DOCK4	dedicator of cytokinesis 4	1.542	4.09E-11
DOK5	docking protein 5	-5.466	4.15E-35
DOT1L	DOT1 like histone lysine methyltransferase	-1.874	0.000000031
DPM3	dolichyl-phosphate mannosyltransferase subunit 3, regulatory	-1.819	0.00000034
DPP7	dipeptidyl peptidase 7	-1.772	0.00000255
DPYSL5	dihydropyrimidinase like 5	6.085	6.83E-100
DRD2	dopamine receptor D2	1.654	1.15E-19
DSE	dermatan sulfate epimerase	2.116	7.93E-23
DSG2	desmoglein 2	1.607	1.13E-21
DSP	desmoplakin	4.847	6.38E-13
DTX3	deltex E3 ubiquitin ligase 3	-1.667	7.11E-10
DUS1L	dihydrouridine synthase 1 like	-1.591	1.06E-09
DUSP10	dual specificity phosphatase 10	1.874	9.27E-18
DUSP5	dual specificity phosphatase 5	1.85	9.58E-18
DUXAP10	double homeobox A pseudogene 10	3.135	0.00000191
DUXAP8	double homeobox A pseudogene 8	5.412	7.23E-09
EDIL3	EGF like repeats and discoidin domains 3	2.756	3.5E-50
EDN1	endothelin 1	-2.74	4.29E-57
EDNRB	endothelin receptor type B	-1.729	1.44E-11
EEFSEC	eukaryotic elongation factor, selenocysteine-tRNA specific	-1.879	4.89E-12
EFEMP2	EGF containing fibulin extracellular matrix protein 2	-3.92	2.82E-25
EFNA1	ephrin A1	2.559	2.33E-10
EHD3	EH domain containing 3	1.798	8.23E-14
ELFN2	extracellular leucine rich repeat and fibronectin type III domain containing 2	1.925	1.03E-20
ELOVL2	ELOVL fatty acid elongase 2	-1.929	0.0000567
EML2	EMAP like 2	-2.007	0.000917
EN1	engrailed homeobox 1	-1.639	0.000000336
ENG	endoglin	-2.715	6.67E-15
ENPP5	ectonucleotide pyrophosphatase/phosphodiesterase family member 5	2.398	0.000000219
EPAS1	endothelial PAS domain protein 1	2.101	1.89E-46
EPB41L4A	erythrocyte membrane protein band 4.1 like 4A	2.224	1E-19
EPB41L4B	erythrocyte membrane protein band 4.1 like 4B	1.948	3.54E-13
EPHA3	EPH receptor A3	-1.676	1.3E-25
EPHA4	EPH receptor A4	-1.628	0.00000532
EPHA5	EPH receptor A5	2.213	3.34E-17

EPHB2	EPH receptor B2	2.581	1.21E-75
EPHX1	epoxide hydrolase 1	1.535	1.33E-12
EPHX4	epoxide hydrolase 4	1.776	0.000000622
EPSTI1	epithelial stromal interaction 1	-1.777	0.00000613
ERAP1	endoplasmic reticulum aminopeptidase 1	1.734	4.9E-26
ERAP2	endoplasmic reticulum aminopeptidase 2	2.334	3.94E-61
ERCC2	ERCC excision repair 2, TFIIH core complex helicase subunit	-1.904	3.56E-26
ERICH3	glutamate rich 3	-3.431	0.000185
ERMP1	endoplasmic reticulum metallopeptidase 1	2.103	3.45E-29
ERO1B	endoplasmic reticulum oxidoreductase 1 beta	1.569	0.00000117
ERP29	endoplasmic reticulum protein 29	1.551	6.57E-18
EVI2A	ecotropic viral integration site 2A	2.237	2.79E-13
EXOSC4	exosome component 4	-1.592	0.0000667
EXTL1	exostosin like glycosyltransferase 1	-44.802	3.23E-21
F11R	F11 receptor	-1.762	2.76E-09
F2RL2	coagulation factor II thrombin receptor like 2	1.766	1.08E-10
F3	coagulation factor III, tissue factor	-2.008	1.17E-33
FABP7	fatty acid binding protein 7	-1.819	6.11E-27
FAM131A	family with sequence similarity 131 member A	-1.839	4.63E-12
FAM131B	family with sequence similarity 131 member B	2.488	2.38E-08
FAM160A1	family with sequence similarity 160 member A1	1.642	0.00000253
FAM171B	family with sequence similarity 171 member B	1.742	2.8E-11
FAM189A1	family with sequence similarity 189 member A1	5.16	4.98E-20
FAM222A	family with sequence similarity 222 member A	-1.738	0.00172
FAM227A	family with sequence similarity 227 member A	3.28	0.000336
FAM229B	family with sequence similarity 229 member B	1.688	0.0000756
FAM43A	family with sequence similarity 43 member A	-1.861	0.00000184
FAM89A	family with sequence similarity 89 member A	2.598	1.92E-11
FAP	fibroblast activation protein alpha	3.686	2.29E-34
FAS	Fas cell surface death receptor	1.641	4.42E-13
FAT2	FAT atypical cadherin 2	42.889	1.61E-40
FAT3	FAT atypical cadherin 3	-1.612	3.57E-12
FBLN1	fibulin 1	1.563	4.08E-11
FBLN5	fibulin 5	6.677	0.0000003
FBXL19-AS1	FBXL19 antisense RNA 1	1.764	0.000328
FBXO4	F-box protein 4	-1.716	6.11E-10
FCGRT	Fc fragment of IgG receptor and transporter	-2.014	1.45E-09

FER	FER tyrosine kinase	-1.626	2.99E-11
FER1L4	fer-1 like family member 4 (pseudogene)	2.908	1.31E-17
FEZF1	FEZ family zinc finger 1	-3.652	0.00000713
FEZF1-AS1	FEZF1 antisense RNA 1	-3.271	4.77E-14
FGD6	FYVE, RhoGEF and PH domain containing 6	2.109	7.13E-11
FIBCD1	fibrinogen C domain containing 1	1.744	0.000357
FILIP1	filamin A interacting protein 1	-1.902	0.00000186
FKBP14	FKBP prolyl isomerase 14	1.559	1.82E-12
FLJ22447	uncharacterized LOC400221	4.067	2.05E-28
FLRT2	fibronectin leucine rich transmembrane protein 2	2.135	2.88E-30
FLRT3	fibronectin leucine rich transmembrane protein 3	-2.322	9.72E-31
FMN2	formin 2	-2.066	2.29E-34
FMNL1	formin like 1	2.213	3.67E-38
FOXA1	forkhead box A1	-1.89	5.44E-12
FOXD3-AS1	FOXD3 antisense RNA 1	-3.718	0.000049
FOXF1	forkhead box F1	5.763	6.41E-16
FOXF2	forkhead box F2	1.672	0.0000345
FOXG1	forkhead box G1	-1.793	1.31E-11
FOXO4	forkhead box O4	1.869	0.0000164
FOXQ1	forkhead box Q1	2.579	2.64E-08
FOXRED2	FAD dependent oxidoreductase domain containing 2	-1.7	2.13E-19
FPR1	formyl peptide receptor 1	7.918	7.42E-39
FRAS1	Fraser extracellular matrix complex subunit 1	2.095	0.000315
FRMD3	FERM domain containing 3	-1.559	1.02E-11
FRZB	frizzled related protein	-2.207	0.00000011
FSIP1	fibrous sheath interacting protein 1	-1.858	0.000107
FST	follistatin	-1.824	1.69E-12
GABBR2	gamma-aminobutyric acid type B receptor subunit 2	-2.384	1.41E-14
GABRE	gamma-aminobutyric acid type A receptor epsilon subunit	3.004	4.84E-19
GACAT2	gastric cancer associated transcript 2	-3.512	0.00000262
GALNT12	polypeptide N-acetylgalactosaminyltransferase 12	-3.32	0.000615
GALNT18	polypeptide N-acetylgalactosaminyltransferase 18	6.061	0.0000114
GALNT6	polypeptide N-acetylgalactosaminyltransferase 6	-2.679	0.00000794
GAP43	growth associated protein 43	-2.231	3.66E-23
GAREM2	GRB2 associated regulator of MAPK1 subtype 2	-3.585	1.12E-14
GAS1	growth arrest specific 1	1.877	0.00000291
GAS6-DT	GAS6 divergent transcript	2.71	3.51E-08

GAS7	growth arrest specific 7	1.721	1.98E-18
GATA2	GATA binding protein 2	-1.654	0.00000449
GATA3	GATA binding protein 3	-2.292	5.88E-29
GBP3	guanylate binding protein 3	1.548	9.34E-12
GBP4	guanylate binding protein 4	4.049	1.55E-22
GCNT1	glucosaminyl (N-acetyl) transferase 1	1.831	2.5E-28
GDPD1	glycerophosphodiester phosphodiesterase domain containing 1	2.157	0.0000472
GFOD1	glucose-fructose oxidoreductase domain containing 1	1.573	0.000000108
GFPT2	glutamine-fructose-6-phosphate transaminase 2	1.962	2.7E-46
GIMAP2	GTPase, IMAP family member 2	3.525	2.02E-22
GIMAP5	GTPase, IMAP family member 5	28.962	1.63E-08
GIMAP6	GTPase, IMAP family member 6	19.018	4.42E-70
GJA1	gap junction protein alpha 1	1.901	3.22E-48
GJA3	gap junction protein alpha 3	2.521	2.38E-12
GJB2	gap junction protein beta 2	2.304	5.34E-61
GLCE	glucuronic acid epimerase	1.54	3.53E-13
GLDC	glycine decarboxylase	2.169	2.73E-19
GLDN	gliomedin	-2.733	0.00000684
GLIS1	GLIS family zinc finger 1	-2.384	3.58E-08
GNAL	G protein subunit alpha L	4.213	7.05E-16
GNAO1	G protein subunit alpha o1	1.872	4.29E-09
GNG7	G protein subunit gamma 7	-12.412	3.7E-17
GOPC	golgi associated PDZ and coiled-coil motif containing	1.693	2.04E-22
GPC4	glypican 4	-1.59	1.63E-19
GPM6A	glycoprotein M6A	-4.538	6.82E-135
GPR1	G protein-coupled receptor 1	3.988	0.00000343
GPR156	G protein-coupled receptor 156	-1.975	0.000129
GPR160	G protein-coupled receptor 160	1.926	0.00000614
GPR63	G protein-coupled receptor 63	1.879	0.00000134
GPRC5B	G protein-coupled receptor class C group 5 member B	-1.66	1.02E-09
GPX3	glutathione peroxidase 3	-4.044	5.68E-15
GRIN2A	glutamate ionotropic receptor NMDA type subunit 2A	3.452	3.59E-11
GRIP1	glutamate receptor interacting protein 1	1.999	9.04E-11
GRPR	gastrin releasing peptide receptor	-3.276	3.03E-20
GSDMC	gasdermin C	3.291	0.0000701
GSN	gelsolin	-1.772	3.57E-17
GULP1	GULP PTB domain containing engulfment adaptor 1	-2.683	3.02E-42

GXYLT2	glucoside xylosyltransferase 2	1.804	5.69E-14
GYG2	glycogenin 2	-3.142	8.01E-12
GYPC	glycophorin C (Gerbich blood group)	-1.707	0.000189
H2AC11	H2A clustered histone 11	-2.288	0.000468
H2AC6	H2A clustered histone 6	-1.726	7.77E-08
H2AW	H2A.W histone	-2.243	0.00049
H2BC12	H2B clustered histone 12	-1.606	0.000000161
HAS3	hyaluronan synthase 3	2.883	9.38E-62
HBP1	HMG-box transcription factor 1	1.594	2.02E-11
HEPH	hephaestin	2.167	4.27E-24
HERC5	HECT and RLD domain containing E3 ubiquitin protein ligase 5	1.996	3.98E-21
HERC6	HECT and RLD domain containing E3 ubiquitin protein ligase family member 6	1.753	0.000000895
HES4	hes family bHLH transcription factor 4	-2.972	0.00000114
HIVEP2	HIVEP zinc finger 2	1.919	4.83E-27
HLA-DPA1	major histocompatibility complex, class II, DP alpha 1	8	8.34E-13
HMCN1	hemicentin 1	2.205	1.39E-22
HMGN5	high mobility group nucleosome binding domain 5	5.282	1.41E-25
HMOX1	heme oxygenase 1	1.782	4E-27
HNRNPUL2-BSCL2	HNRNPUL2-BSCL2 readthrough (NMD candidate)	-2.87	0.0000957
HOXA6	homeobox A6	-1.896	0.000797
HOXA7	homeobox A7	-4.225	3.84E-08
HOXB5	homeobox B5	-3.58	0.0000473
HOXD13	homeobox D13	-1.888	0.000264
HPCAL1	hippocalcin like 1	-1.599	3.99E-11
HPS1	HPS1 biogenesis of lysosomal organelles complex 3 subunit 1	-1.624	3.27E-09
HR	HR lysine demethylase and nuclear receptor corepressor	-2.221	1.79E-19
HSPA5	heat shock protein family A (Hsp70) member 5	1.589	1.45E-26
ICAM1	intercellular adhesion molecule 1	-2.088	0.000942
ICOSLG/LOC102723996	inducible T cell costimulator ligand	1.809	9.83E-15
IDO1	indoleamine 2,3-dioxygenase 1	6.136	9.45E-19
IFI35	interferon induced protein 35	1.696	0.00000084
IFIT1	interferon induced protein with tetratricopeptide repeats 1	2.071	7.93E-10
IFIT3	interferon induced protein with tetratricopeptide repeats 3	1.668	1.33E-10
IGFBP2	insulin like growth factor binding protein 2	-1.778	2.63E-16
IGFBP3	insulin like growth factor binding protein 3	2.047	1.53E-36
IGFBP4	insulin like growth factor binding protein 4	7.783	8.13E-307
IGFBP5	insulin like growth factor binding protein 5	-4.757	6.68E-183

IGFBP6	insulin like growth factor binding protein 6	1.592	0.00000276
IGSF10	immunoglobulin superfamily member 10	3.117	5.99E-10
IL11	interleukin 11	2.625	5.17E-36
IL13RA2	interleukin 13 receptor subunit alpha 2	2.404	1.93E-46
IL15RA	interleukin 15 receptor subunit alpha	2.315	5.92E-08
IL17RD	interleukin 17 receptor D	1.828	1.05E-12
IL24	interleukin 24	9.158	0.00000516
IL27RA	interleukin 27 receptor subunit alpha	2.194	1.98E-20
IL4R	interleukin 4 receptor	5.011	3.94E-46
IL7	interleukin 7	2.089	0.0000152
INAVA	innate immunity activator	-3.887	0.00000521
INHBA	inhibin subunit beta A	-1.859	1.29E-18
IQGAP2	IQ motif containing GTPase activating protein 2	-3.111	6.62E-11
IRF1	interferon regulatory factor 1	1.931	5.18E-19
IRF2BPL	interferon regulatory factor 2 binding protein like	-1.769	0.0000104
IRX1	iroquois homeobox 1	-1.864	0.00000975
ISLR	immunoglobulin superfamily containing leucine rich repeat	-2.609	0.00015
ISYNA1	inositol-3-phosphate synthase 1	-1.919	0.0000164
ITGA1	integrin subunit alpha 1	2.135	9.01E-21
ITGA11	integrin subunit alpha 11	2.101	8.91E-08
ITGA2	integrin subunit alpha 2	2.682	4.09E-73
ITGB3	integrin subunit beta 3	2.002	1.63E-29
ITGBL1	integrin subunit beta like 1	-1.701	2.03E-10
ITPK1	inositol-tetrakisphosphate 1-kinase	-1.602	1.16E-12
JAG2	jagged canonical Notch ligand 2	3.125	0.000000149
JUP	junction plakoglobin	-2.752	8.76E-12
KALRN	kalirin RhoGEF kinase	2.136	1.71E-20
KCNAB2	potassium voltage-gated channel subfamily A regulatory beta subunit 2	2.344	2.13E-27
KCNB1	potassium voltage-gated channel subfamily B member 1	16.671	6.96E-28
KCND3	potassium voltage-gated channel subfamily D member 3	-6.595	4.74E-19
KCNH1	potassium voltage-gated channel subfamily H member 1	-5.907	0.0000118
KCNIP3	potassium voltage-gated channel interacting protein 3	1.965	3.09E-08
KCNIP4	potassium voltage-gated channel interacting protein 4	-2.827	5.69E-10
KCNK2	potassium two pore domain channel subfamily K member 2	1.803	0.000161
KCNK5	potassium two pore domain channel subfamily K member 5	16.382	0.000000252
KCNMA1	potassium calcium-activated channel subfamily M alpha 1	3.006	4.22E-136
KCNN4	potassium calcium-activated channel subfamily N member 4	1.856	1.21E-22

KCTD15	potassium channel tetramerization domain containing 15	-2.279	8.59E-49
KDELR3	KDEL endoplasmic reticulum protein retention receptor 3	1.62	3.27E-13
KDM5B	lysine demethylase 5B	1.683	2.64E-22
KIAA0319	KIAA0319	2.06	1.41E-09
KIF17	kinesin family member 17	-2.713	0.0000483
KIRREL3	kirre like nephrin family adhesion molecule 3	-1.734	7.06E-10
KITLG	KIT ligand	2.576	1.83E-43
KLF17	Kruppel like factor 17	2.727	0.000237
KLHL24	kelch like family member 24	1.609	0.0000299
KRT13	keratin 13	-2.91	0.00736
KRT18	keratin 18	-1.878	1.82E-13
KRT18P17	keratin 18 pseudogene 17	-6.294	0.0000324
KRT81	keratin 81	-2.693	3.29E-32
KRT87P	keratin 87 pseudogene	-3.856	4.81E-09
KSR1	kinase suppressor of ras 1	1.573	2.08E-13
L1CAM	L1 cell adhesion molecule	2.628	2.77E-35
L3HYPDH	trans-L-3-hydroxyproline dehydratase	-1.593	0.00000316
LAIR1	leukocyte associated immunoglobulin like receptor 1	4.909	0.0000379
LAMC2	laminin subunit gamma 2	2.224	2.15E-10
LCP1	lymphocyte cytosolic protein 1	1.668	6.12E-13
LDHA	lactate dehydrogenase A	1.58	2.08E-10
LGR4	leucine rich repeat containing G protein-coupled receptor 4	3.498	2.53E-55
LGR5	leucine rich repeat containing G protein-coupled receptor 5	-2.778	1.09E-14
LIN7B	lin-7 homolog B, crumbs cell polarity complex component	-1.754	0.000434
LINC00052	long intergenic non-protein coding RNA 52	-2.676	0.000315
LINC00327	long intergenic non-protein coding RNA 327	-2.551	0.00000035
LINC00460	long intergenic non-protein coding RNA 460	-2.929	3.56E-14
LINC00461	long intergenic non-protein coding RNA 461	-1.755	1.46E-22
LINC00632	long intergenic non-protein coding RNA 632	-2.521	0.000000031
LINC00639	long intergenic non-protein coding RNA 639	-2.195	8.01E-12
LINC00665	long intergenic non-protein coding RNA 665	-1.546	4.36E-12
LINC00707	long intergenic non-protein coding RNA 707	-1.728	6.17E-20
LINC00963	long intergenic non-protein coding RNA 963	1.558	3.78E-12
LINC01111	long intergenic non-protein coding RNA 1111	-3.314	2.2E-10
LINC01303	long intergenic non-protein coding RNA 1303	2.823	0.000000974
LINC01503	long intergenic non-protein coding RNA 1503	2.141	0.000028
LINC02593	long intergenic non-protein coding RNA 2593	-1.617	4.74E-08

LINC02732		-2.726	9.8E-13
LINGO2	leucine rich repeat and Ig domain containing 2	-6.8	5.51E-09
LIPE	lipase E, hormone sensitive type	-2.689	1.81E-10
LIPG	lipase G, endothelial type	2.916	0.00000647
LLGL2	LLGL scribble cell polarity complex component 2	-2.977	3.18E-12
LMCD1	LIM and cysteine rich domains 1	-1.97	2.54E-18
LMO3	LIM domain only 3	-2.053	1.72E-24
LNCAROD	lncRNA activating regulator of DKK1	-1.714	5.18E-16
LOC100129534	small nuclear ribonucleoprotein polypeptide N pseudogene	2.464	0.000286
LOC100505501	uncharacterized LOC100505501	1.959	0.000295
LOC100507516	uncharacterized LOC100507516	-3.786	3.89E-33
LOC101928663	uncharacterized LOC101928663	1.897	4.44E-10
LOC101930370	uncharacterized LOC101930370	1.947	0.0000824
LOC105369203		-2.169	2.12E-20
LOC107984948		2.192	0.000000828
LOC339192	uncharacterized LOC339192	2.341	0.000252
LOC643201	centrosomal protein 192kDa pseudogene	2.403	6.2E-19
LOX	lysyl oxidase	3.94	8.54E-53
LOXL1-AS1	LOXL1 antisense RNA 1	3.329	4.89E-18
LOXL4	lysyl oxidase like 4	-2.089	3.82E-24
LPL	lipoprotein lipase	-3.88	6.06E-39
LPXN	leupaxin	2.068	8.75E-37
LRAT	lecithin retinol acyltransferase	-2.558	7.45E-23
LRATD2	LRAT domain containing 2	-2.089	6.57E-34
LRP1	LDL receptor related protein 1	1.68	3.67E-15
LRP1B	LDL receptor related protein 1B	2.063	1.73E-17
LRRC17	leucine rich repeat containing 17	3.777	2.59E-28
LRRC2	leucine rich repeat containing 2	-2.189	3.87E-15
LRRC45	leucine rich repeat containing 45	-2.058	2.72E-08
LRRC4C	leucine rich repeat containing 4C	1.572	0.000000344
LRRC8B	leucine rich repeat containing 8 VRAC subunit B	-2.072	3.51E-26
LRRK1	leucine rich repeat kinase 1	2.038	0.0000375
LTBP1	latent transforming growth factor beta binding protein 1	3.274	5.01E-85
LYPD1	LY6/PLAUR domain containing 1	-1.574	3.95E-25
MAGEH1	MAGE family member H1	5.161	4.08E-37
MAMDC2	MAM domain containing 2	1.936	2.56E-39
MAN1A1	mannosidase alpha class 1A member 1	1.844	3.31E-27

MAN1C1	mannosidase alpha class 1C member 1	-2.165	0.000509
MAOA	monoamine oxidase A	4.633	0.0000115
MAP1A	microtubule associated protein 1A	1.973	2.27E-41
MARCHF1	membrane associated ring-CH-type finger 1	-1.947	7.08E-13
MARCHF10	membrane associated ring-CH-type finger 10	-6.337	1.09E-11
MARCKSL1	MARCKS like 1	-1.529	3.76E-10
MARVELD1	MARVEL domain containing 1	-1.626	4.29E-14
MAST4	microtubule associated serine/threonine kinase family member 4	1.943	8.85E-18
MATN2	matrilin 2	3.829	1.51E-147
MATN3	matrilin 3	-1.936	0.00317
MCM5	minichromosome maintenance complex component 5	-1.878	7.76E-29
MCTP1	multiple C2 and transmembrane domain containing 1	2.579	1.09E-23
MDGA1	MAM domain containing glycosylphosphatidylinositol anchor 1	2.117	5.05E-32
MDK	midkine	-4.893	1.48E-33
MEF2C	myocyte enhancer factor 2C	-1.725	6.84E-10
MEIS2	Meis homeobox 2	-1.652	5.05E-17
MEIS3P1	Meis homeobox 3 pseudogene 1	-3.763	7.79E-17
MEIS3P2	Meis homeobox 3 pseudogene 2	-2.98	0.000351
MELTF	melanotransferrin	-2.317	3.67E-12
MEST	mesoderm specific transcript	2.02	7.04E-42
METTL25	methyltransferase like 25	1.929	0.000338
MFSD3	major facilitator superfamily domain containing 3	-1.829	0.0000256
MGC12916	uncharacterized protein MGC12916	2.483	1.47E-08
MGLL	monoglyceride lipase	1.841	7.94E-35
MGP	matrix Gla protein	2.134	1.13E-36
MGRN1	mahogunin ring finger 1	-1.626	7.7E-09
MGST1	microsomal glutathione S-transferase 1	1.584	6.72E-24
MIR100HG	mir-100-let-7a-2-mir-125b-1 cluster host gene	1.974	4.67E-26
MIRLET7BHG	MIRLET7B host gene	-1.859	8.79E-13
MME	membrane metalloendopeptidase	2.313	6.62E-23
MMP14	matrix metallopeptidase 14	2.709	3.19E-91
MMP17	matrix metallopeptidase 17	-6.829	1.44E-16
MMP3	matrix metallopeptidase 3	5.395	4E-16
MMP7	matrix metallopeptidase 7	5.167	5.19E-106
MMP9	matrix metallopeptidase 9	5.757	0.00000972
MOXD1	monooxygenase DBH like 1	-2.058	5.13E-53
MPP2	membrane palmitoylated protein 2	-1.777	1.72E-10

MRAP2	melanocortin 2 receptor accessory protein 2	-3.592	0.000609
MROCKI	MARCKS cis regulating lncRNA promoter of cytokines and inflammation	-3.356	7.82E-28
MSANTD2	Myb/SANT DNA binding domain containing 2	-1.671	4.58E-14
MT-RNR1	s-rRNA	1.844	9.25E-21
MT-RNR2	l-rRNA	1.894	7.5E-47
MT-TT	tRNA	2.158	0.0000935
MT1E	metallothionein 1E	-2.022	9.21E-16
MTCL1	microtubule crosslinking factor 1	-1.829	9.82E-33
MTFP1	mitochondrial fission process 1	-2.255	0.00654
MTRNR2L1	MT-RNR2 like 1	1.911	0.00000686
MTRNR2L10	MT-RNR2 like 10	2.305	1.87E-08
MTRNR2L12	MT-RNR2 like 12	1.878	1.61E-18
MTRNR2L2	MT-RNR2 like 2	1.763	1.44E-10
MTRNR2L8	MT-RNR2 like 8	1.635	0.00000394
MTSS1	MTSS I-BAR domain containing 1	8.902	4.35E-44
MYBL2	MYB proto-oncogene like 2	-1.766	1.48E-22
MYEOV	myeloma overexpressed	-1.982	4.29E-18
MYH15	myosin heavy chain 15	2.33	4.29E-09
MYH16	myosin heavy chain 16 pseudogene	3.334	0.000487
MYH3	myosin heavy chain 3	2.61	0.000000374
MYO5C	myosin VC	-1.591	0.00000157
MYOM1	myomesin 1	-2.883	0.00068
MYRF	myelin regulatory factor	1.608	3.76E-12
NACAD	NAC alpha domain containing	-1.829	0.000000128
NACC2	NACC family member 2	-1.683	1.69E-12
NAP1L2	nucleosome assembly protein 1 like 2	2.962	4.96E-11
NAPA-AS1	NAPA antisense RNA 1	2.6	0.000106
NCOR2	nuclear receptor corepressor 2	-1.947	0.000000282
NDUFV2	NADH:ubiquinone oxidoreductase core subunit V2	2.295	0.000118
NEAT1	nuclear paraspeckle assembly transcript 1	1.533	1.19E-17
NEDD9	neural precursor cell expressed, developmentally down-regulated 9	-1.666	1.3E-20
NEGR1	neuronal growth regulator 1	-1.592	5.36E-08
NEO1	neogenin 1	2.024	4.28E-29
NES	nestin	-2.734	8.27E-102
NEUROG2	neurogenin 2	-3.742	0.0000456
NFASC	neurofascin	2.278	1.4E-44
NFIA	nuclear factor I A	-2.018	0.000511

NIPAL2	NIPA like domain containing 2	-2.491	0.000000257
NIPSNAP1	nipsnap homolog 1	-1.885	1.78E-15
NISCH	nischarin	-1.654	1.25E-15
NME3	NME/NM23 nucleoside diphosphate kinase 3	-3.916	3.12E-09
NNMT	nicotinamide N-methyltransferase	1.776	6.46E-19
NOL6	nucleolar protein 6	-1.579	4.27E-18
NOTCH1	notch receptor 1	-2.329	7.18E-09
NPAS2	neuronal PAS domain protein 2	2.326	5.52E-28
NPEPL1	aminopeptidase like 1	-1.808	0.0000105
NPTXR	neuronal pentraxin receptor	2.752	0.000000128
NPY1R	neuropeptide Y receptor Y1	-2.315	1.11E-09
NQO2-AS1		4.745	0.000535
NR1D2	nuclear receptor subfamily 1 group D member 2	1.569	9.45E-19
NR1H4	nuclear receptor subfamily 1 group H member 4	-9.412	1.25E-32
NRBP2	nuclear receptor binding protein 2	-1.542	0.000000185
NRG2	neuregulin 2	-1.935	5.9E-15
NRXN3	neurexin 3	-4.214	4.87E-20
NSUN5	NOP2/Sun RNA methyltransferase 5	-1.977	4.46E-11
NT5C	5', 3'-nucleotidase, cytosolic	-1.692	7.49E-08
NT5DC2	5'-nucleotidase domain containing 2	-1.703	2.28E-21
NT5M	5',3'-nucleotidase, mitochondrial	-1.872	0.0000489
NTN1	netrin 1	3.566	3.76E-34
NTN4	netrin 4	2.923	5.76E-59
NTNG1	netrin G1	3.57	5.48E-42
NTRK3	neurotrophic receptor tyrosine kinase 3	-2.437	1.3E-25
NUDT11	nudix hydrolase 11	2.722	0.000000978
NUDT16L1	nudix hydrolase 16 like 1	-1.821	0.000000155
NUDT4	nudix hydrolase 4	-1.547	9.19E-17
NXPH4	neurexophilin 4	1.98	0.000000331
OAS1	2'-5'-oligoadenylate synthetase 1	3.082	0.000462
OCLN	occludin	1.854	0.000103
OLFML2B	olfactomedin like 2B	1.971	3.4E-24
ONECUT1	one cut homeobox 1	-3.15	0.00545
OPLAH	5-oxoprolinase, ATP-hydrolysing	-2.935	8.25E-09
OPN3	opsin 3	-1.589	0.00000204
OPRD1	opioid receptor delta 1	-10.386	0.000000555
OTOGL	otogelin like	-1.812	4.13E-08

OTULINL	OTU deubiquitinase with linear linkage specificity like	1.9	0.00000042
P3H2	prolyl 3-hydroxylase 2	1.834	1.48E-32
P4HA2	prolyl 4-hydroxylase subunit alpha 2	1.604	1.44E-17
P4HTM	prolyl 4-hydroxylase, transmembrane	-1.554	0.000000033
PADI2	peptidyl arginine deiminase 2	-8.004	0.000000636
PALM2AKAP2	PALM2 and AKAP2 fusion	-2.798	0.00459
PAPLN	papilin, proteoglycan like sulfated glycoprotein	10.178	6.17E-09
PAPPA	pappalysin 1	2.136	2.98E-29
PARM1	prostate androgen-regulated mucin-like protein 1	6.336	1.38E-26
PARVB	parvin beta	-2.785	0.000544
PASK	PAS domain containing serine/threonine kinase	-1.527	8.76E-12
PAX8	paired box 8	2.972	3.2E-27
PAX8-AS1	PAX8 antisense RNA 1	5.236	1.02E-17
PBX4	PBX homeobox 4	-3.157	4.52E-11
PBXIP1	PBX homeobox interacting protein 1	1.704	2.23E-16
PCDH10	protocadherin 10	-4.398	1.31E-101
PCDH20	protocadherin 20	-8.649	6.68E-183
PCDH9	protocadherin 9	-1.62	0.00000205
PCDHGC3	protocadherin gamma subfamily C, 3	1.928	1.23E-31
PCDHGC5	protocadherin gamma subfamily C, 5	2.02	1.61E-10
PCSK4	proprotein convertase subtilisin/kexin type 4	-2.162	0.00117
PDCD1LG2	programmed cell death 1 ligand 2	1.583	0.00000197
PDE2A	phosphodiesterase 2A	3.198	0.000000547
PDE4A	phosphodiesterase 4A	-2.182	1.33E-20
PDE5A	phosphodiesterase 5A	3.097	0.00109
PDGFC	platelet derived growth factor C	1.536	1.33E-16
PDGFD	platelet derived growth factor D	1.646	2.45E-13
PDK4	pyruvate dehydrogenase kinase 4	4.156	5.95E-16
PDZD4	PDZ domain containing 4	1.928	0.00000127
PEAR1	platelet endothelial aggregation receptor 1	-2.38	7.84E-10
PEG10	paternally expressed 10	-2.032	6.83E-64
PER3	period circadian regulator 3	1.703	4.31E-21
PFKP	phosphofructokinase, platelet	-1.553	1.55E-13
PGF	placental growth factor	-2.295	1.9E-16
PHLDA2	pleckstrin homology like domain family A member 2	-2.639	1.35E-08
PHRF1	PHD and ring finger domains 1	-1.794	2.52E-09
PIAS4	protein inhibitor of activated STAT 4	-1.721	0.000000184

PICK1	protein interacting with PRKCA 1	-2.398	1.41E-26
PIDD1	p53-induced death domain protein 1	-1.81	0.000227
PIEZO2	piezo type mechanosensitive ion channel component 2	-4.77	3.59E-28
PIGZ	phosphatidylinositol glycan anchor biosynthesis class Z	2.09	0.00113
PIK3CD-AS2	PIK3CD antisense RNA 2	-3.667	0.0000832
PITPNM3	PITPNM family member 3	1.664	0.000000201
PKD1P5	polycystin 1, transient receptor potential channel interacting pseudogene 5	-2.089	0.00023
PKMYT1	protein kinase, membrane associated tyrosine/threonine 1	-2.06	1.45E-08
PKN1	protein kinase N1	-1.569	3.95E-11
PKP2	plakophilin 2	-1.568	3.72E-11
PKP3	plakophilin 3	-4.478	0.00000174
PLAAT4	phospholipase A and acyltransferase 4	2.096	8.32E-09
PLAC8	placenta associated 8	-14.677	1.81E-11
PLAT	plasminogen activator, tissue type	-2.616	2.05E-66
PLAUR	plasminogen activator, urokinase receptor	1.671	4.6E-21
PLB1	phospholipase B1	1.932	0.000249
PLCB4	phospholipase C beta 4	2.068	1.02E-11
PLEKHA7	pleckstrin homology domain containing A7	-1.999	2.63E-09
PLEKHG4B	pleckstrin homology and RhoGEF domain containing G4B	2.195	0.000858
PLEKHN1	pleckstrin homology domain containing N1	-1.794	0.0000399
PLP1	proteolipid protein 1	-4.355	0.000000646
PLS1	plastin 1	1.601	5.06E-13
PLXNA4	plexin A4	2.804	8.09E-10
PLXND1	plexin D1	-1.554	1.69E-12
PNMA2	PNMA family member 2	2.557	0.000000108
PODXL	podocalyxin like	2.088	1.12E-62
PODXL2	podocalyxin like 2	1.895	1.08E-11
POSTN	periostin	-34.329	3.17E-37
POU2F2	POU class 2 homeobox 2	2.489	0.0000765
PPFIA2	PTPRF interacting protein alpha 2	-2.017	0.000000104
PPM1H	protein phosphatase, Mg2+/Mn2+ dependent 1H	4.584	2.65E-09
PPM1M	protein phosphatase, Mg2+/Mn2+ dependent 1M	-1.826	2.64E-08
PPP1R35	protein phosphatase 1 regulatory subunit 35	-1.714	8.61E-08
PPP1R3B	protein phosphatase 1 regulatory subunit 3B	1.654	3.2E-10
PPP2R2B	protein phosphatase 2 regulatory subunit Bbeta	-1.91	9.16E-36
PPP2R2C	protein phosphatase 2 regulatory subunit Bgamma	5.685	2.19E-13
PPP4R4	protein phosphatase 4 regulatory subunit 4	3.351	0.0000605

PREX1	phosphatidylinositol-3,4,5-trisphosphate dependent Rac exchange factor 1	2.032	8.23E-29
PRKAR1B	protein kinase cAMP-dependent type I regulatory subunit beta	-1.839	1.73E-09
PRKCZ	protein kinase C zeta	1.735	0.000000031
PRKG1	protein kinase cGMP-dependent 1	6.435	1.15E-11
PRLR	prolactin receptor	-1.975	1.1E-09
PROX1	prospero homeobox 1	-1.775	6.83E-08
PRR16	proline rich 16	1.873	1.81E-09
PRSS23	serine protease 23	1.858	1.87E-36
PRSS35	serine protease 35	-2.227	1.5E-34
PRUNE2	prune homolog 2 with BCH domain	-1.878	1.14E-09
PSD4	pleckstrin and Sec7 domain containing 4	2.436	1.35E-23
PSG5	pregnancy specific beta-1-glycoprotein 5	-5.325	0.000000773
PSG9	pregnancy specific beta-1-glycoprotein 9	-3.653	0.000000334
PSMB9	proteasome subunit beta 9	1.721	7.31E-08
PTCH1	patched 1	-2.383	3.2E-10
PTMAP5	prothymosin alpha pseudogene 5	-1.583	0.000272
PTPRB	protein tyrosine phosphatase receptor type B	-1.667	6.46E-18
PTPRE	protein tyrosine phosphatase receptor type E	2.072	1.15E-23
PTPRU	protein tyrosine phosphatase receptor type U	3.08	1.54E-31
PTPRZ1	protein tyrosine phosphatase receptor type Z1	-1.542	0.00000163
PTX3	pentraxin 3	1.827	2.69E-21
PXMP4	peroxisomal membrane protein 4	-1.666	5.29E-09
PXYLP1	2-phosphoxylose phosphatase 1	1.856	3.97E-10
PYCR1	pyrroline-5-carboxylate reductase 1	-1.716	5.82E-13
RAB11FIP4	RAB11 family interacting protein 4	-2.946	1.23E-08
RAB29	RAB29, member RAS oncogene family	1.658	2.85E-18
RAB6B	RAB6B, member RAS oncogene family	1.657	5.44E-11
RAET1G	retinoic acid early transcript 1G	2.018	0.000151
RAMP1	receptor activity modifying protein 1	-2.432	0.000563
RANGRF	RAN guanine nucleotide release factor	-1.59	2.08E-10
RAP1GAP2	RAP1 GTPase activating protein 2	1.795	0.000000857
RASGRF1	Ras protein specific guanine nucleotide releasing factor 1	-2.563	0.0000678
RASSF7	Ras association domain family member 7	-2.182	0.0000198
REEP1	receptor accessory protein 1	2.295	3.11E-08
REXO1	RNA exonuclease 1 homolog	-1.733	0.0000366
RFX8	RFX family member 8, lacking RFX DNA binding domain	2.236	9.37E-23
RGMA	repulsive guidance molecule BMP co-receptor a	-3.126	3.6E-24

RGS20	regulator of G protein signaling 20	2.006	1.13E-13
RGS7	regulator of G protein signaling 7	-1.842	1.5E-13
RHBDD3	rhomboid domain containing 3	-1.622	0.0000409
RHOJ	ras homolog family member J	-2.602	3.4E-61
RHOT2	ras homolog family member T2	-1.657	0.00000534
RIMBP2	RIMS binding protein 2	11.445	3.17E-55
RIMS3	regulating synaptic membrane exocytosis 3	-1.629	0.000000345
RIN1	Ras and Rab interactor 1	-1.681	0.000105
RIN2	Ras and Rab interactor 2	1.649	5.2E-21
RIPOR2	RHO family interacting cell polarization regulator 2	-4.163	3.59E-106
RNF112	ring finger protein 112	-4.107	1.04E-11
RNF126	ring finger protein 126	-1.985	0.0000849
RNF152	ring finger protein 152	2.231	0.000000058
RNF157	ring finger protein 157	-1.702	1.49E-13
RNF208	ring finger protein 208	-4.672	0.000000355
RNF24	ring finger protein 24	2.121	1.08E-45
ROR1	receptor tyrosine kinase like orphan receptor 1	2.159	5.55E-17
RORA	RAR related orphan receptor A	2.981	0.00102
ROS1	ROS proto-oncogene 1, receptor tyrosine kinase	9.447	1.19E-90
RP11_134G810		1.877	0.000221
RP11_138I14		1.851	0.000394
RP11_159D122		1.68	0.00000632
RP11_268J155		-2.281	7.93E-10
RP11_284F2110		3.501	8.63E-54
RP11_284F217		2.87	0.000000012
RP11_284F219		4.475	3.39E-09
RP11_31F191		3.343	0.000847
RP11_320L112		2.908	0.00000582
RP11_39C101		1.746	0.000000345
RP11_424C202		-1.753	0.000189
RP11_888D104		2.759	0.000136
RP1_140K85		3.168	0.0000394
RPS6KA2	ribosomal protein S6 kinase A2	2.948	1.28E-33
RPUSD1	RNA pseudouridine synthase domain containing 1	-1.894	0.0000292
RTL9	retrotransposon Gag like 9	2.141	2.17E-20
RTN2	reticulon 2	2.025	3.21E-09
S100A2	S100 calcium binding protein A2	3.306	4.58E-54

S100A3	S100 calcium binding protein A3	2.233	0.00000123
S100A4	S100 calcium binding protein A4	2.146	4.67E-11
S100B	S100 calcium binding protein B	-3.455	8.68E-72
SAA1	serum amyloid A1	6.632	0.00000548
SAMD10	sterile alpha motif domain containing 10	-1.849	9.35E-08
SAMD11	sterile alpha motif domain containing 11	-2.534	2.82E-13
SAMD5	sterile alpha motif domain containing 5	-3.21	6.17E-34
SAMD9L	sterile alpha motif domain containing 9 like	1.652	6.76E-15
SATB1	SATB homeobox 1	1.761	0.00000083
SCN4B	sodium voltage-gated channel beta subunit 4	-2.184	0.000208
SCNN1A	sodium channel epithelial 1 alpha subunit	3.974	1.61E-18
SCNN1B	sodium channel epithelial 1 beta subunit	3.38	8.23E-08
SCPEP1	serine carboxypeptidase 1	1.732	2.36E-29
SCUBE3	signal peptide, CUB domain and EGF like domain containing 3	1.573	6.28E-12
SDCBP2-AS1	SDCBP2 antisense RNA 1	2.239	0.000665
SEL1L3	SEL1L family member 3	2.174	1.44E-50
SELENOP	selenoprotein P	2.595	0.00000177
SELL	selectin L	3.646	3.72E-10
SEMA3B	semaphorin 3B	-5.78	8.11E-48
SEMA3C	semaphorin 3C	1.877	2.54E-37
SEMA3F	semaphorin 3F	6.566	1.18E-21
SEMA5A	semaphorin 5A	-1.636	0.0000665
SEMA7A	semaphorin 7A (John Milton Hagen blood group)	1.93	8.51E-34
SEPTIN3	septin 3	1.666	0.00000365
SEPTIN5	septin 5	-1.782	5.29E-08
SEPTIN6	septin 6	1.83	1.81E-12
SERINC2	serine incorporator 2	5.888	2.38E-69
SERPINA1	serpin family A member 1	2.51	1.39E-15
SERPINA5	serpin family A member 5	2.157	0.0000649
SERPINE1	serpin family E member 1	2.024	5.43E-41
SERPINE2	serpin family E member 2	1.746	2.22E-30
SERPINH1	serpin family H member 1	-3.539	8.57E-63
SERTAD4-AS1	SERTAD4 antisense RNA 1	-1.959	0.00000269
SETBP1	SET binding protein 1	1.636	4.29E-12
SF3A2	splicing factor 3a subunit 2	-1.556	0.000000251
SFR1	SWI5 dependent homologous recombination repair protein 1	1.574	0.000000892
SFRP1	secreted frizzled related protein 1	1.742	6.69E-16

SGK1	serum/glucocorticoid regulated kinase 1	-1.522	1.37E-12
SGSH	N-sulfoglucosamine sulfohydrolase	-1.989	1.66E-23
SH2D4A	SH2 domain containing 4A	-2.931	0.00000128
SH3BGRL2	SH3 domain binding glutamate rich protein like 2	1.972	9.96E-09
SH3D21	SH3 domain containing 21	-3.155	3.35E-37
SH3PXD2A	SH3 and PX domains 2A	1.953	3.46E-36
SHC2	SHC adaptor protein 2	-2.599	0.0000566
SHISAL1	shisa like 1	3.754	0.00000281
SHROOM2	shroom family member 2	-3.461	0.00000742
SIAE	sialic acid acetylesterase	1.577	4.21E-12
SIGLEC22P	sialic acid binding Ig like lectin 22, pseudogene	6.637	0.00000666
SIGMAR1	sigma non-opioid intracellular receptor 1	-1.597	0.00000807
SIPA1	signal-induced proliferation-associated 1	-1.916	0.0000049
SLAIN1	SLAIN motif family member 1	-1.515	5.8E-12
SLC12A7	solute carrier family 12 member 7	3.216	9.47E-18
SLC12A8	solute carrier family 12 member 8	-2.564	2.59E-08
SLC15A2	solute carrier family 15 member 2	2.838	0.00000304
SLC16A14	solute carrier family 16 member 14	-2.161	0.0000256
SLC16A2	solute carrier family 16 member 2	1.568	8.78E-20
SLC16A3	solute carrier family 16 member 3	-1.749	7.87E-08
SLC16A7	solute carrier family 16 member 7	-3.038	0.00000932
SLC16A9	solute carrier family 16 member 9	-2.298	3.79E-38
SLC1A3	solute carrier family 1 member 3	1.898	4.43E-34
SLC20A1	solute carrier family 20 member 1	1.546	1.38E-23
SLC22A4	solute carrier family 22 member 4	1.785	0.000000122
SLC25A22	solute carrier family 25 member 22	-1.577	0.00000705
SLC25A23	solute carrier family 25 member 23	1.94	3.47E-30
SLC25A27	solute carrier family 25 member 27	4.028	0.000167
SLC25A28	solute carrier family 25 member 28	-1.653	5.41E-14
SLC26A11	solute carrier family 26 member 11	-2.286	2.45E-16
SLC27A6	solute carrier family 27 member 6	-3.191	0.000262
SLC2A12	solute carrier family 2 member 12	7.566	4.49E-54
SLC2A13	solute carrier family 2 member 13	3.614	4.54E-10
SLC2A8	solute carrier family 2 member 8	-1.562	0.000000461
SLC30A3	solute carrier family 30 member 3	4.183	4.06E-08
SLC37A2	solute carrier family 37 member 2	2.434	1.32E-08
SLC38A11	solute carrier family 38 member 11	1.794	0.000291

SLC38A2	solute carrier family 38 member 2	1.61	4.48E-17
SLC38A3	solute carrier family 38 member 3	4.405	3.62E-27
SLC39A8	solute carrier family 39 member 8	1.56	8.59E-10
SLC40A1	solute carrier family 40 member 1	-1.941	0.000966
SLC43A1	solute carrier family 43 member 1	1.773	0.000584
SLC43A2	solute carrier family 43 member 2	-1.715	4.38E-08
SLC43A3	solute carrier family 43 member 3	-1.602	2.75E-11
SLC44A5	solute carrier family 44 member 5	-5.212	7.04E-18
SLC7A11	solute carrier family 7 member 11	-1.683	2.2E-14
SLC8A1	solute carrier family 8 member A1	3.513	5.44E-67
SLCO4A1	solute carrier organic anion transporter family member 4A1	3.202	9.96E-41
SLCO5A1	solute carrier organic anion transporter family member 5A1	-2.19	4.33E-16
SLPI	secretory leukocyte peptidase inhibitor	22.511	9.14E-25
SMIM25	small integral membrane protein 25	2.019	0.000217
SMPDL3B	sphingomyelin phosphodiesterase acid like 3B	4.235	2.16E-08
SMTN	smoothelin	-1.94	1.51E-14
SNHG11	small nucleolar RNA host gene 11	-1.98	0.0000312
SNHG19	small nucleolar RNA host gene 19	-1.71	0.00000497
SNHG25		-1.944	0.000315
SNHG9	small nucleolar RNA host gene 9	-2.093	1.83E-08
SOCS2	suppressor of cytokine signaling 2	-1.678	9.35E-17
SOCS2-AS1	SOCS2 antisense RNA 1	-1.749	7.81E-08
SOCS5	suppressor of cytokine signaling 5	1.559	1.5E-15
SORBS3	sorbin and SH3 domain containing 3	-1.679	8.28E-20
SORCS2	sortilin related VPS10 domain containing receptor 2	6.164	5.14E-37
SOX13	SRY-box transcription factor 13	-1.704	2.64E-22
SOX2	SRY-box transcription factor 2	-1.525	7.59E-11
SOX2-OT	SOX2 overlapping transcript	-3.73	5.66E-109
SOX6	SRY-box transcription factor 6	-2.499	0.00000727
SP140	SP140 nuclear body protein	2.572	0.000751
SPA17	sperm autoantigenic protein 17	1.621	2.25E-09
SPAAR	small regulatory polypeptide of amino acid response	-1.663	0.000000206
SPATC1L	spermatogenesis and centriole associated 1 like	-2.338	3.2E-09
SPINT1	serine peptidase inhibitor, Kunitz type 1	-2.504	0.00000219
SPOCK1	SPARC (osteonectin), cwcv and kazal like domains proteoglycan 1	-1.666	1.14E-25
SPP1	secreted phosphoprotein 1	-2.433	1.05E-69
SPSB1	splA/ryanodine receptor domain and SOCS box containing 1	2.68	3.87E-33

SQOR	sulfide quinone oxidoreductase	2.022	7.05E-18
SSBP4	single stranded DNA binding protein 4	-2.007	0.0000353
ST8SIA1	ST8 alpha-N-acetyl-neuraminide alpha-2,8-sialyltransferase 1	3.017	0.0000345
STAT5A	signal transducer and activator of transcription 5A	-2.034	0.00000742
STC1	stanniocalcin 1	1.818	1.41E-31
STC2	stanniocalcin 2	-1.564	8.02E-13
STEAP1	STEAP family member 1	5.707	5.36E-28
STEAP2	STEAP2 metalloreductase	2.567	0.00000907
STK17A	serine/threonine kinase 17a	1.586	2.32E-18
STK32B	serine/threonine kinase 32B	-1.811	2.5E-09
STMN3	stathmin 3	1.949	2.63E-11
STOM	stomatin	-2.093	7.3E-44
STON2	stonin 2	1.784	2.29E-12
STRA6	stimulated by retinoic acid 6	-3.206	1.29E-36
STRN4	striatin 4	-1.539	2.79E-08
STXBP6	syntaxin binding protein 6	1.633	0.0000141
SUGT1P1	SUGT1 pseudogene 1	-1.982	0.00052
SULF1	sulfatase 1	-4.301	1.86E-71
SULF2	sulfatase 2	1.66	1.48E-22
SURF2	surfeit 2	-1.821	0.000171
SUSD1	sushi domain containing 1	-1.961	0.00000171
SUSD2	sushi domain containing 2	2.146	0.000666
SYDE1	synapse defective Rho GTPase homolog 1	-1.649	5.22E-08
SYNC	syncoilin, intermediate filament protein	1.675	6.72E-17
SYNE3	spectrin repeat containing nuclear envelope family member 3	-2.206	0.000166
SYNGR1	synaptogyrin 1	-2.983	7.61E-11
SYNPO	synaptopodin	1.865	1.86E-26
SYTL2	synaptotagmin like 2	1.587	0.00000715
TACR1	tachykinin receptor 1	2.171	5.41E-20
TAGLN	transgelin	-2.988	4.33E-18
TAGLN3	transgelin 3	1.867	0.00101
TBC1D4	TBC1 domain family member 4	2.36	4.63E-40
TBC1D8	TBC1 domain family member 8	1.591	0.00000019
TBC1D8B	TBC1 domain family member 8B	1.705	0.0000138
TBX2	T-box transcription factor 2	-1.638	0.0000118
TCN1	transcobalamin 1	3.125	4.9E-26
TCN2	transcobalamin 2	1.702	0.000373

TEAD4	TEA domain transcription factor 4	-1.594	0.00000185
TEF	TEF transcription factor, PAR bZIP family member	2.241	4.61E-36
TELO2	telomere maintenance 2	-1.809	0.000074
TENM2	teneurin transmembrane protein 2	1.623	1.28E-24
TENM4	teneurin transmembrane protein 4	1.626	7.98E-16
TEX15	testis expressed 15, meiosis and synapsis associated	4.88	2.81E-08
TEX9	testis expressed 9	1.612	0.0000127
TFAP2B	transcription factor AP-2 beta	-2.125	0.00461
TFAP2C	transcription factor AP-2 gamma	-1.613	9.24E-25
TFCP2L1	transcription factor CP2 like 1	2.093	0.000299
TFEB	transcription factor EB	1.69	9.51E-08
TGFB2	transforming growth factor beta 2	1.628	1.51E-13
TGFB3	transforming growth factor beta 3	2.337	5.3E-23
TGFBI	transforming growth factor beta induced	1.536	4.69E-18
TGM2	transglutaminase 2	-1.903	2.09E-27
THEMIS2	thymocyte selection associated family member 2	-3.696	0.0000227
THNSL2	threonine synthase like 2	-4.932	8.36E-13
THSD1	thrombospondin type 1 domain containing 1	2.273	7.58E-26
TIAM1	T cell lymphoma invasion and metastasis 1	1.762	4.6E-16
TIGD5	tigger transposable element derived 5	-1.801	0.000122
TIMP4	TIMP metallopeptidase inhibitor 4	2.156	6.17E-13
TKTL1	transketolase like 1	3.465	2.62E-29
TLCD4	TLC domain containing 4	1.559	0.000000404
TLR1	toll like receptor 1	2.271	1.34E-08
TLR3	toll like receptor 3	2.517	0.000153
TLR6	toll like receptor 6	1.788	7E-14
TLX2	T cell leukemia homeobox 2	-2.376	0.00102
TM4SF18	transmembrane 4 L six family member 18	1.686	0.0000123
TMC7	transmembrane channel like 7	1.702	0.0000635
TMCC1-AS1	TMCC1 antisense RNA 1 (head to head)	1.86	0.000136
TMCO6	transmembrane and coiled-coil domains 6	-2.356	0.000338
TMEM100	transmembrane protein 100	-2.676	0.00000698
TMEM140	transmembrane protein 140	1.862	0.0000508
TMEM158	transmembrane protein 158 (gene/pseudogene)	3.105	1.66E-79
TMEM171	transmembrane protein 171	-3.343	0.000000208
TMEM179	transmembrane protein 179	2.938	0.00000903
TMEM200A	transmembrane protein 200A	-4.624	1.21E-08

TMEM200B	transmembrane protein 200B	-1.787	7.24E-09
TMEM200C	transmembrane protein 200C	-1.871	4.42E-16
TMEM201	transmembrane protein 201	-1.73	2.33E-08
TMEM220	transmembrane protein 220	-3.503	5.44E-11
TMEM229B	transmembrane protein 229B	-4.131	1.47E-08
TMEM25	transmembrane protein 25	-2.872	4.55E-11
TMEM255A	transmembrane protein 255A	3.066	3.47E-14
TMEM255B	transmembrane protein 255B	6.102	4.14E-14
TMEM38A	transmembrane protein 38A	1.794	0.000000163
TMEM40	transmembrane protein 40	-3.112	0.000275
TMEM71	transmembrane protein 71	2.654	3.32E-20
TMSB15A	thymosin beta 15a	4.206	7.84E-16
TMTC1	transmembrane O-mannosyltransferase targeting cadherins 1	2.252	4.12E-39
TMUB1	transmembrane and ubiquitin like domain containing 1	-1.898	0.000000759
TNC	tenascin C	1.842	8.79E-33
TNFAIP6	TNF alpha induced protein 6	2.475	0.00101
TNFRSF21	TNF receptor superfamily member 21	2.926	3.63E-80
TNFSF10	TNF superfamily member 10	3.003	1.67E-34
TNFSF4	TNF superfamily member 4	-9.034	1.31E-115
TNNT1	troponin T1, slow skeletal type	2.322	3.53E-14
TOR4A	torsin family 4 member A	-3.395	1.53E-18
TOX	thymocyte selection associated high mobility group box	2.217	2.73E-13
TP53I11	tumor protein p53 inducible protein 11	2.87	2.66E-53
TPPP	tubulin polymerization promoting protein	2.821	1.99E-10
TPRG1	tumor protein p63 regulated 1	3.157	5.13E-09
TRABD	TraB domain containing	-1.829	0.0000723
TRABD2A	TraB domain containing 2A	3.873	1.37E-39
TRAF1	TNF receptor associated factor 1	2.083	1.82E-27
TRANK1	tetratricopeptide repeat and ankyrin repeat containing 1	1.752	6.78E-09
TRAPPC9	trafficking protein particle complex 9	-1.673	2.07E-13
TRIM38	tripartite motif containing 38	-3.059	1.19E-25
TRIM6	tripartite motif containing 6	2.945	2.14E-10
TRPC3	transient receptor potential cation channel subfamily C member 3	-3.484	1.05E-10
TRPM3	transient receptor potential cation channel subfamily M member 3	-3.026	1.28E-16
TSEN54	tRNA splicing endonuclease subunit 54	-1.637	5.56E-13
TSHZ2	teashirt zinc finger homeobox 2	4.002	0.000000171
TSPAN1	tetraspanin 1	2.23	0.000975

TSPAN12	tetraspanin 12	5.181	0.000000453
TSTD1	thiosulfate sulfurtransferase like domain containing 1	-2.131	7.03E-10
TTBK1	tau tubulin kinase 1	-4.738	2.46E-28
TTC39A	tetratricopeptide repeat domain 39A	-3.43	0.0000647
TTC3P1	tetratricopeptide repeat domain 3 pseudogene 1	2.038	1.23E-20
TTYH2	tweety family member 2	2.043	0.000202
TUBA4A	tubulin alpha 4a	1.557	1.71E-08
TUBB2A	tubulin beta 2A class IIa	-1.552	9.53E-09
TUBB2B	tubulin beta 2B class IIb	-2.648	5.58E-27
TUBB4A	tubulin beta 4A class IVa	2.204	0.000000241
TXK	TXK tyrosine kinase	-2.777	4.86E-10
TYMSOS	TYMS opposite strand	-2.557	0.000000211
UBE2L6	ubiquitin conjugating enzyme E2 L6	1.645	2.21E-09
UCA1	urothelial cancer associated 1	82.794	2.51E-26
UCN2	urocortin 2	1.718	3.51E-11
UFSP1	UFM1 specific peptidase 1 (inactive)	-2.448	0.000819
UNC5B	unc-5 netrin receptor B	2.051	0.000389
USP46-AS1	USP46 antisense RNA 1	2.025	0.00153
UST	uronyl 2-sulfotransferase	1.823	1.17E-24
VAC14-AS1	VAC14 antisense RNA 1	3.205	0.000657
VAT1L	vesicle amine transport 1 like	-8.944	1.45E-22
VDR	vitamin D receptor	1.862	1.33E-12
VEGFA	vascular endothelial growth factor A	1.849	2.71E-38
VGLL3	vestigial like family member 3	-2.457	3.48E-09
VIT	vitrin	-2.857	7.08E-24
VPS35L	VPS35 endosomal protein sorting factor like	-1.643	1.38E-08
VSNL1	visinin like 1	-1.622	2.33E-08
VSTM4	V-set and transmembrane domain containing 4	4.949	0.0000156
VWA5A	von Willebrand factor A domain containing 5A	2.461	2.68E-10
VWCE	von Willebrand factor C and EGF domains	3.092	0.0000605
VWDE	von Willebrand factor D and EGF domains	-1.831	1.58E-11
WDR34	WD repeat domain 34	-1.538	0.000000881
WDR90	WD repeat domain 90	-1.819	0.0000016
WIPI1	WD repeat domain, phosphoinositide interacting 1	1.811	7.36E-12
WNK2	WNK lysine deficient protein kinase 2	-4.518	2.56E-08
WNT5B	Wnt family member 5B	1.808	1.01E-15
WSCD1	WSC domain containing 1	-1.62	5.15E-09

WT1	WT1 transcription factor	-1.812	0.00003
WWC1	WW and C2 domain containing 1	1.735	2.5E-15
WWC3	WWC family member 3	-1.548	6.04E-15
XDH	xanthine dehydrogenase	3.199	9.22E-39
YDJC	YdjC chitooligosaccharide deacetylase homolog	-1.947	0.0000335
YPEL2	yippee like 2	1.844	0.000753
YPEL3	yippee like 3	-1.991	0.0000834
ZADH2	zinc binding alcohol dehydrogenase domain containing 2	1.691	1.46E-16
ZBTB38	zinc finger and BTB domain containing 38	1.538	2.13E-18
ZBTB47	zinc finger and BTB domain containing 47	-1.742	0.000000457
ZC3H12B	zinc finger CCCH-type containing 12B	2.212	0.000175
ZCWPW1	zinc finger CW-type and PWWP domain containing 1	1.972	0.00000614
ZDHHC12	zinc finger DHHC-type containing 12	-1.574	0.00000499
ZFHX2	zinc finger homeobox 2	2.231	0.00000794
ZFHX4-AS1	ZFHX4 antisense RNA 1	-2.962	7.07E-17
ZFP82	ZFP82 zinc finger protein	-1.648	0.000000607
ZFPM2-AS1	ZFPM2 antisense RNA 1	-2.319	1.82E-08
ZFYVE28	zinc finger FYVE-type containing 28	2.075	0.00012
ZGPAT	zinc finger CCCH-type and G-patch domain containing	-1.793	0.000444
ZIC2	Zic family member 2	5.998	0.00000023
ZMIZ1	zinc finger MIZ-type containing 1	1.588	8.14E-17
ZMIZ1-AS1	ZMIZ1 antisense RNA 1	2.43	2.95E-12
ZNF239	zinc finger protein 239	-1.624	0.0000191
ZNF415	zinc finger protein 415	-1.61	0.00000191
ZNF469	zinc finger protein 469	-1.59	0.000142
ZNF506	zinc finger protein 506	-2.186	4.32E-14
ZNF542P	zinc finger protein 542, pseudogene	-1.717	1.01E-13
ZNF593	zinc finger protein 593	-1.584	0.000157
ZNF598	zinc finger protein 598	-1.745	0.0000243
ZNF620	zinc finger protein 620	-1.861	1.47E-08
ZNF660	zinc finger protein 660	-4.087	0.000000461
ZNF677	zinc finger protein 677	-1.755	2.56E-10
ZNF688	zinc finger protein 688	-1.903	0.00276
ZNF69	zinc finger protein 69	-1.934	0.00000147
ZNF702P	zinc finger protein 702, pseudogene	-2.676	2.46E-08
ZNF730	zinc finger protein 730	-2.211	0.0000413
ZNF771	zinc finger protein 771	-1.837	0.00104

ZNF793	zinc finger protein 793	-3.166	3.9E-13
ZNF853	zinc finger protein 853	-2.96	8.99E-21
ZSCAN5A	zinc finger and SCAN domain containing 5A	1.949	0.000000794
ZSWIM5	zinc finger SWIM-type containing 5	-1.652	0.000000167
