# Supplementary material for: mTORC2/Rac1 Pathway Predisposes Cancer Aggressiveness in IDH1-Mutated Glioma
Source: Cancers (Basel). 2020 Mar 26;12(4):787. doi: 10.3390/cancers12040787 (PMC7226122; doi:10.3390/cancers12040787)
Supplement: Supplementary file 1 [file cancers-12-00787-s001.zip › cancers-733430-revised-suppl-3.18/Supplementary Table 2.pdf]

Supplementary Table 2. List of differentially expressed cell movement related genes in U251 IDH1 R132H compared with U251 IDH1 WT

Gene Symbol	Entrez Gene Name	Fold Change	FDR
A2M	alpha-2-macroglobulin	1.621	3.04E-19
ABCA1	ATP binding cassette subfamily A member 1	1.726	3.22E-27
ABCA4	ATP binding cassette subfamily A member 4	3.462	0.000074
ACKR3	atypical chemokine receptor 3	6.536	5.38E-09
ACTA2	actin alpha 2, smooth muscle	1.674	7.62E-10
ADORA2B	adenosine A2b receptor	1.569	4.84E-19
ADRB2	adrenoceptor beta 2	2.406	0.0000882
AGT	angiotensinogen	1.879	0.00902
AHNAK	AHNAK nucleoprotein	1.722	6.65E-19
AJAP1	adherens junctions associated protein 1	-3.059	3.31E-09
AJUBA	ajuba LIM protein	-1.552	1.95E-12
ALK	ALK receptor tyrosine kinase	-3.854	2.99E-16
AMOT	angiomotin	-1.603	1.86E-15
ANGPTL4	angiopoietin like 4	7.751	2.5E-24
APLN	apelin	-1.833	0.00000355
ARHGAP24	Rho GTPase activating protein 24	13.636	1.6E-19
ARHGDIA	Rho GDP dissociation inhibitor alpha	-1.633	0.000000307
ARRB1	arrestin beta 1	1.832	0.0000138
ASIC1	acid sensing ion channel subunit 1	1.862	3.01E-09
ATOH8	atonal bHLH transcription factor 8	-2.78	0.000000518
AZGP1	alpha-2-glycoprotein 1, zinc-binding	-6.456	9.34E-114
BCAN	brevican	3.501	8.63E-54
BCAR3	BCAR3 adaptor protein, NSP family member	1.588	1.94E-18
BHLHE40	basic helix-loop-helix family member e40	1.776	1.65E-29
BHLHE41	basic helix-loop-helix family member e41	2.669	1.57E-51
BMP4	bone morphogenetic protein 4	6.467	0.00000848
BOC	BOC cell adhesion associated, oncogene regulated	1.806	4.05E-19
C3	complement C3	7.404	7.74E-28
C3AR1	complement C3a receptor 1	2.566	0.000764
CA9	carbonic anhydrase 9	-2.025	4.18E-24
CARD10	caspase recruitment domain family member 10	-1.741	9.84E-08
CASP1	caspase 1	1.876	0.00000248
CCN2	cellular communication network factor 2	-1.86	6.31E-54
CD55	CD55 molecule (Cromer blood group)	3.675	1.84E-13
CD82	CD82 molecule	2.865	2.64E-76

CD93	CD93 molecule	3.717	0.000000976
CD200	CD200 molecule	-2.28	3.28E-11
CD274	CD274 molecule	-1.688	3.29E-17
CDKN1A	cyclin dependent kinase inhibitor 1A	1.706	1.94E-17
CELSR2	cadherin EGF LAG seven-pass G-type receptor 2	-1.586	1.36E-13
CEMIP	cell migration inducing hyaluronidase 1	2.334	1.54E-20
CGA	glycoprotein hormones, alpha polypeptide	-3.603	8.64E-08
CHFR	checkpoint with forkhead and ring finger domains	-4.147	4.13E-36
CHI3L1	chitinase 3 like 1	38.204	7.44E-269
CHRD	chordin	-2.433	0.00115
CLCA2	chloride channel accessory 2	3.86	0.0000115
CLCN4	chloride voltage-gated channel 4	-1.667	1.11E-08
CLDN1	claudin 1	2.003	6.6E-11
CLU	clusterin	2.442	1.98E-35
CMTM8	CKLF like MARVEL transmembrane domain containing 8	-1.817	2.23E-11
CNR1	cannabinoid receptor 1	-2.776	3.45E-65
CNTNAP2	contactin associated protein like 2	6.052	3.32E-31
COL11A1	collagen type XI alpha 1 chain	-1.807	5.44E-32
COL17A1	collagen type XVII alpha 1 chain	3.841	3.96E-94
CORO1A	coronin 1A	-1.667	0.000215
CORO1B	coronin 1B	-1.567	0.00000026
CPEB1	cytoplasmic polyadenylation element binding protein 1	2.063	1.19E-08
CRYAB	crystallin alpha B	-5.072	3.67E-121
CSF1	colony stimulating factor 1	1.716	1.92E-22
CSF2	colony stimulating factor 2	2.847	0.000000504
CTNNA2	catenin alpha 2	-3.667	2.02E-42
CTNND2	catenin delta 2	-1.703	1.61E-10
CTSH	cathepsin H	1.854	3.22E-24
CTSS	cathepsin S	3.604	5.28E-34
CTSV	cathepsin V	1.922	1.15E-11
CTSZ	cathepsin Z	-1.888	1.34E-17
CXADR	CXADR Ig-like cell adhesion molecule	2.294	1.04E-29
CXCL12	C-X-C motif chemokine ligand 12	5.381	1.91E-28
DAB1	DAB adaptor protein 1	-4.372	5.76E-09
DCLK1	doublecortin like kinase 1	1.64	4.03E-11
DIO2	iodothyronine deiodinase 2	-4.564	6.28E-67
DIRAS3	DIRAS family GTPase 3	-1.552	4.68E-12

DLC1	DLC1 Rho GTPase activating protein	2.1	1.49E-21
DLL1	delta like canonical Notch ligand 1	2.146	0.0000406
DNM1	dynamin 1	1.656	1.05E-20
DOCK3	dedicator of cytokinesis 3	2.056	3.13E-13
DOCK4	dedicator of cytokinesis 4	1.542	4.09E-11
DPYSL5	dihydropyrimidinase like 5	6.085	6.83E-100
DRD2	dopamine receptor D2	1.654	1.15E-19
DSE	dermatan sulfate epimerase	2.116	7.93E-23
DSG2	desmoglein 2	1.607	1.13E-21
DUSP10	dual specificity phosphatase 10	1.874	9.27E-18
EDIL3	EGF like repeats and discoidin domains 3	2.756	3.5E-50
EDN1	endothelin 1	-2.74	4.29E-57
EDNRB	endothelin receptor type B	-1.729	1.44E-11
EFNA1	ephrin A1	2.559	2.33E-10
EN1	engrailed homeobox 1	-1.639	0.000000336
ENG	endoglin	-2.715	6.67E-15
EPAS1	endothelial PAS domain protein 1	2.101	1.89E-46
EPB41L4B	erythrocyte membrane protein band 4.1 like 4B	1.948	3.54E-13
EPHA3	EPH receptor A3	-1.676	1.3E-25
EPHA4	EPH receptor A4	-1.628	0.00000532
EPHA5	EPH receptor A5	2.213	3.34E-17
EPHB2	EPH receptor B2	2.581	1.21E-75
ERAP1	endoplasmic reticulum aminopeptidase 1	1.734	4.9E-26
F3	coagulation factor III, tissue factor	-2.008	1.17E-33
F11R	F11 receptor	-1.762	2.76E-09
FABP7	fatty acid binding protein 7	-1.819	6.11E-27
FAM43A	family with sequence similarity 43 member A	-1.861	0.00000184
FAP	fibroblast activation protein alpha	3.686	2.29E-34
FAS	Fas cell surface death receptor	1.641	4.42E-13
FAT3	FAT atypical cadherin 3	-1.612	3.57E-12
FBLN1	fibulin 1	1.563	4.08E-11
FBLN5	fibulin 5	6.677	0.0000003
FBXO4	F-box protein 4	-1.716	6.11E-10
FER	FER tyrosine kinase	-1.626	2.99E-11
FEZF1	FEZ family zinc finger 1	-3.652	0.00000713
FLRT2	fibronectin leucine rich transmembrane protein 2	2.135	2.88E-30
FLRT3	fibronectin leucine rich transmembrane protein 3	-2.322	9.72E-31

FOXA1	forkhead box A1	-1.89	5.44E-12
FOXF1	forkhead box F1	5.763	6.41E-16
FOXF2	forkhead box F2	1.672	0.0000345
FOXG1	forkhead box G1	-1.793	1.31E-11
FOXO4	forkhead box O4	1.869	0.0000164
FOXQ1	forkhead box Q1	2.579	2.64E-08
FPR1	formyl peptide receptor 1	7.918	7.42E-39
FST	follistatin	-1.824	1.69E-12
GAP43	growth associated protein 43	-2.231	3.66E-23
GAS1	growth arrest specific 1	1.877	0.00000291
GATA2	GATA binding protein 2	-1.654	0.00000449
GATA3	GATA binding protein 3	-2.292	5.88E-29
GCNT1	glucosaminyl (N-acetyl) transferase 1	1.831	2.5E-28
GJA1	gap junction protein alpha 1	1.901	3.22E-48
GJB2	gap junction protein beta 2	2.304	5.34E-61
GNAL	G protein subunit alpha L	4.213	7.05E-16
GNAO1	G protein subunit alpha o1	1.872	4.29E-09
GPM6A	glycoprotein M6A	-4.538	6.82E-135
GSN	gelsolin	-1.772	3.57E-17
HAS3	hyaluronan synthase 3	2.883	9.38E-62
HBP1	HMG-box transcription factor 1	1.594	2.02E-11
HMOX1	heme oxygenase 1	1.782	4E-27
HOXA7	homeobox A7	-4.225	3.84E-08
HSPA5	heat shock protein family A (Hsp70) member 5	1.589	1.45E-26
ICAM1	intercellular adhesion molecule 1	-2.088	0.000942
ICOSLG/LOC102723996	inducible T cell costimulator ligand	1.809	9.83E-15
IDO1	indoleamine 2,3-dioxygenase 1	6.136	9.45E-19
IGFBP2	insulin like growth factor binding protein 2	-1.778	2.63E-16
IGFBP3	insulin like growth factor binding protein 3	2.047	1.53E-36
IGFBP4	insulin like growth factor binding protein 4	7.783	8.13E-307
IGFBP5	insulin like growth factor binding protein 5	-4.757	6.68E-183
IGFBP6	insulin like growth factor binding protein 6	1.592	0.00000276
IL7	interleukin 7	2.089	0.0000152
IL11	interleukin 11	2.625	5.17E-36
IL24	interleukin 24	9.158	0.00000516
IL13RA2	interleukin 13 receptor subunit alpha 2	2.404	1.93E-46
IL15RA	interleukin 15 receptor subunit alpha	2.315	5.92E-08

IL27RA	interleukin 27 receptor subunit alpha	2.194	1.98E-20
IL4R	interleukin 4 receptor	5.011	3.94E-46
INHBA	inhibin subunit beta A	-1.859	1.29E-18
ITGA1	integrin subunit alpha 1	2.135	9.01E-21
ITGA2	integrin subunit alpha 2	2.682	4.09E-73
ITGB3	integrin subunit beta 3	2.002	1.63E-29
ITGBL1	integrin subunit beta like 1	-1.701	2.03E-10
JAG2	jagged canonical Notch ligand 2	3.125	0.000000149
JUP	junction plakoglobin	-2.752	8.76E-12
KALRN	kalirin RhoGEF kinase	2.136	1.71E-20
KCNK2	potassium two pore domain channel subfamily K member 2	1.803	0.000161
KCNK5	potassium two pore domain channel subfamily K member 5	16.382	0.000000252
KCNMA1	potassium calcium-activated channel subfamily M alpha 1	3.006	4.22E-136
KCNN4	potassium calcium-activated channel subfamily N member 4	1.856	1.21E-22
KDM5B	lysine demethylase 5B	1.683	2.64E-22
KIAA0319	KIAA0319	2.06	1.41E-09
KIRREL3	kirre like nephrin family adhesion molecule 3	-1.734	7.06E-10
KITLG	KIT ligand	2.576	1.83E-43
KLF17	Kruppel like factor 17	2.727	0.000237
L1CAM	L1 cell adhesion molecule	2.628	2.77E-35
LAMC2	laminin subunit gamma 2	2.224	2.15E-10
LCP1	lymphocyte cytosolic protein 1	1.668	6.12E-13
LDHA	lactate dehydrogenase A	1.58	2.08E-10
LGR4	leucine rich repeat containing G protein-coupled receptor 4	3.498	2.53E-55
LIPE	lipase E, hormone sensitive type	-2.689	1.81E-10
LMCD1	LIM and cysteine rich domains 1	-1.97	2.54E-18
LOX	lysyl oxidase	3.94	8.54E-53
LRP1	LDL receptor related protein 1	1.68	3.67E-15
MARCKSL1	MARCKS like 1	-1.529	3.76E-10
MATN2	matrilin 2	3.829	1.51E-147
MDGA1	MAM domain containing glycosylphosphatidylinositol anchor 1	2.117	5.05E-32
MDK	midkine	-4.893	1.48E-33
MEF2C	myocyte enhancer factor 2C	-1.725	6.84E-10
MELTF	melanotransferrin	-2.317	3.67E-12
MGLL	monoglyceride lipase	1.841	7.94E-35
MGP	matrix Gla protein	2.134	1.13E-36
MME	membrane metalloendopeptidase	2.313	6.62E-23

MMP3	matrix metallopeptidase 3	5.395	4E-16
MMP7	matrix metallopeptidase 7	5.167	5.19E-106
MMP9	matrix metallopeptidase 9	5.757	0.00000972
MMP14	matrix metallopeptidase 14	2.709	3.19E-91
MTSS1	MTSS I-BAR domain containing 1	8.902	4.35E-44
MYRF	myelin regulatory factor	1.608	3.76E-12
NEDD9	neural precursor cell expressed, developmentally down-regulated 9	-1.666	1.3E-20
NEO1	neogenin 1	2.024	4.28E-29
NES	nestin	-2.734	8.27E-102
NEUROG2	neurogenin 2	-3.742	0.0000456
NFASC	neurofascin	2.278	1.4E-44
NFIA	nuclear factor I A	-2.018	0.000511
NISCH	nischarin	-1.654	1.25E-15
NOTCH1	notch receptor 1	-2.329	7.18E-09
NR1H4	nuclear receptor subfamily 1 group H member 4	-9.412	1.25E-32
NRXN3	neurexin 3	-4.214	4.87E-20
NTN1	netrin 1	3.566	3.76E-34
NTN4	netrin 4	2.923	5.76E-59
NTRK3	neurotrophic receptor tyrosine kinase 3	-2.437	1.3E-25
NUDT16L1	nudix hydrolase 16 like 1	-1.821	0.000000155
OCLN	occludin	1.854	0.000103
ONECUT1	one cut homeobox 1	-3.15	0.00545
OPRD1	opioid receptor delta 1	-10.386	0.000000555
OTULINL	OTU deubiquitinase with linear linkage specificity like	1.9	0.00000042
P4HA2	prolyl 4-hydroxylase subunit alpha 2	1.604	1.44E-17
PAPPA	pappalysin 1	2.136	2.98E-29
PARVB	parvin beta	-2.785	0.000544
PCDH10	protocadherin 10	-4.398	1.31E-101
PCSK4	proprotein convertase subtilisin/kexin type 4	-2.162	0.00117
PDCD1LG2	programmed cell death 1 ligand 2	1.583	0.00000197
PDE2A	phosphodiesterase 2A	3.198	0.000000547
PDGFD	platelet derived growth factor D	1.646	2.45E-13
PGF	placental growth factor	-2.295	1.9E-16
PHLDA2	pleckstrin homology like domain family A member 2	-2.639	1.35E-08
PICK1	protein interacting with PRKCA 1	-2.398	1.41E-26
PKN1	protein kinase N1	-1.569	3.95E-11
PKP2	plakophilin 2	-1.568	3.72E-11

PLAT	plasminogen activator, tissue type	-2.616	2.05E-66
PLAUR	plasminogen activator, urokinase receptor	1.671	4.6E-21
PLP1	proteolipid protein 1	-4.355	0.000000646
PLS1	plastin 1	1.601	5.06E-13
PLXNA4	plexin A4	2.804	8.09E-10
PLXND1	plexin D1	-1.554	1.69E-12
PODXL2	podocalyxin like 2	1.895	1.08E-11
PODXL	podocalyxin like	2.088	1.12E-62
POSTN	periostin	-34.329	3.17E-37
PREX1	phosphatidylinositol-3,4,5-trisphosphate dependent Rac exchange factor 1	2.032	8.23E-29
PRKAR1B	protein kinase cAMP-dependent type I regulatory subunit beta	-1.839	1.73E-09
PRKCZ	protein kinase C zeta	1.735	0.000000031
PRKG1	protein kinase cGMP-dependent 1	6.435	1.15E-11
PRLR	prolactin receptor	-1.975	1.1E-09
PROX1	prospero homeobox 1	-1.775	6.83E-08
PTPRU	protein tyrosine phosphatase receptor type U	3.08	1.54E-31
PTPRZ1	protein tyrosine phosphatase receptor type Z1	-1.542	0.00000163
PTX3	pentraxin 3	1.827	2.69E-21
RAMP1	receptor activity modifying protein 1	-2.432	0.000563
RASGRF1	Ras protein specific guanine nucleotide releasing factor 1	-2.563	0.0000678
RIN1	Ras and Rab interactor 1	-1.681	0.000105
RIPOR2	RHO family interacting cell polarization regulator 2	-4.163	3.59E-106
ROR1	receptor tyrosine kinase like orphan receptor 1	2.159	5.55E-17
RORA	RAR related orphan receptor A	2.981	0.00102
S100A2	S100 calcium binding protein A2	3.306	4.58E-54
S100A4	S100 calcium binding protein A4	2.146	4.67E-11
S100B	S100 calcium binding protein B	-3.455	8.68E-72
SAA1	serum amyloid A1	6.632	0.00000548
SATB1	SATB homeobox 1	1.761	0.00000083
SCNN1A	sodium channel epithelial 1 alpha subunit	3.974	1.61E-18
SCPEP1	serine carboxypeptidase 1	1.732	2.36E-29
SCUBE3	signal peptide, CUB domain and EGF like domain containing 3	1.573	6.28E-12
SELL	selectin L	3.646	3.72E-10
SEMA3B	semaphorin 3B	-5.78	8.11E-48
SEMA3C	semaphorin 3C	1.877	2.54E-37
SEMA3F	semaphorin 3F	6.566	1.18E-21
SEMA5A	semaphorin 5A	-1.636	0.0000665

SERPINA1	serpin family A member 1	2.51	1.39E-15
SERPINA5	serpin family A member 5	2.157	0.0000649
SERPINE1	serpin family E member 1	2.024	5.43E-41
SERPINE2	serpin family E member 2	1.746	2.22E-30
SERPINH1	serpin family H member 1	-3.539	8.57E-63
SFRP1	secreted frizzled related protein 1	1.742	6.69E-16
SGK1	serum/glucocorticoid regulated kinase 1	-1.522	1.37E-12
SH3PXD2A	SH3 and PX domains 2A	1.953	3.46E-36
SLC1A3	solute carrier family 1 member 3	1.898	4.43E-34
SLC2A8	solute carrier family 2 member 8	-1.562	0.000000461
SLC8A1	solute carrier family 8 member A1	3.513	5.44E-67
SLPI	secretory leukocyte peptidase inhibitor	22.511	9.14E-25
SOCS2	suppressor of cytokine signaling 2	-1.678	9.35E-17
SORBS3	sorbin and SH3 domain containing 3	-1.679	8.28E-20
SOX2	SRY-box transcription factor 2	-1.525	7.59E-11
SPINT1	serine peptidase inhibitor, Kunitz type 1	-2.504	0.00000219
SPP1	secreted phosphoprotein 1	-2.433	1.05E-69
SPSB1	splA/ryanodine receptor domain and SOCS box containing 1	2.68	3.87E-33
SRSF1	serine and arginine rich splicing factor 1	1.68	0.00000632
ST8SIA1	ST8 alpha-N-acetyl-neuraminide alpha-2,8-sialyltransferase 1	3.017	0.0000345
STAT5A	signal transducer and activator of transcription 5A	-2.034	0.00000742
STC1	stanniocalcin 1	1.818	1.41E-31
STMN3	stathmin 3	1.949	2.63E-11
SULF1	sulfatase 1	-4.301	1.86E-71
TACR1	tachykinin receptor 1	2.171	5.41E-20
TAGLN	transgelin	-2.988	4.33E-18
TBX2	T-box transcription factor 2	-1.638	0.0000118
TEAD4	TEA domain transcription factor 4	-1.594	0.00000185
TENM2	teneurin transmembrane protein 2	1.623	1.28E-24
TFAP2C	transcription factor AP-2 gamma	-1.613	9.24E-25
TGFB2	transforming growth factor beta 2	1.628	1.51E-13
TGFB3	transforming growth factor beta 3	2.337	5.3E-23
TGFBI	transforming growth factor beta induced	1.536	4.69E-18
TGM2	transglutaminase 2	-1.903	2.09E-27
TIAM1	T cell lymphoma invasion and metastasis 1	1.762	4.6E-16
TIMP4	TIMP metallopeptidase inhibitor 4	2.156	6.17E-13
TLR3	toll like receptor 3	2.517	0.000153

TMEM201	transmembrane protein 201	-1.73	2.33E-08
TNC	tenascin C	1.842	8.79E-33
TNFAIP6	TNF alpha induced protein 6	2.475	0.00101
TNFRSF21	TNF receptor superfamily member 21	2.926	3.63E-80
TNFSF4	TNF superfamily member 4	-9.034	1.31E-115
TNFSF10	TNF superfamily member 10	3.003	1.67E-34
TUBB2B	tubulin beta 2B class IIb	-2.648	5.58E-27
TXK	TXK tyrosine kinase	-2.777	4.86E-10
UBE2L6	ubiquitin conjugating enzyme E2 L6	1.645	2.21E-09
UNC5B	unc-5 netrin receptor B	2.051	0.000389
VDR	vitamin D receptor	1.862	1.33E-12
VEGFA	vascular endothelial growth factor A	1.849	2.71E-38
VSNL1	visinin like 1	-1.622	2.33E-08
WNT5B	Wnt family member 5B	1.808	1.01E-15
WT1	WT1 transcription factor	-1.812	0.00003
WWC1	WW and C2 domain containing 1	1.735	2.5E-15
XDH	xanthine dehydrogenase	3.199	9.22E-39
