# Supplementary material for: mTORC2/Rac1 Pathway Predisposes Cancer Aggressiveness in IDH1-Mutated Glioma
Source: Cancers (Basel). 2020 Mar 26;12(4):787. doi: 10.3390/cancers12040787 (PMC7226122; doi:10.3390/cancers12040787)
Supplement: Supplementary file 1 [file cancers-12-00787-s001.zip › cancers-733430-revised-suppl-3.18/Supplementary Table 3.pdf]

Supplementary Table 3. List of differentially expressed genes in U251 IDH1 R132C compared with U251 IDH1 WT

Gene Symbol	Entrez Gene Name	Fold Change	FDR
**A2M**	alpha-2-macroglobulin	3.563	6.56E-133
**ABCA1**	ATP binding cassette subfamily A member 1	1.722	2.68E-27
**ABCA4**	ATP binding cassette subfamily A member 4	2.997	0.000469
**ABCB11**	ATP binding cassette subfamily B member 11	-3.243	0.000775
**ABCB4**	ATP binding cassette subfamily B member 4	-21.743	1.71E-09
**ABHD8**	abhydrolase domain containing 8	-1.596	0.0000909
**ABI3BP**	ABI family member 3 binding protein	-3.192	2E-109
**ABTB2**	ankyrin repeat and BTB domain containing 2	1.685	6.03E-14
**AC0030921**		18.291	6.38E-16
**AC0041583**		-3.434	0.000428
**AC0051501**		-85.896	2.42E-80
**AC0057867**		2.821	0.000346
**AC0936277**		1.939	0.00102
**AC0937871**		-2.743	1.19E-13
**ACKR3**	atypical chemokine receptor 3	9.625	5.48E-16
**ACP6**	acid phosphatase 6, lysophosphatidic	-2.005	9.48E-23
**ACSL5**	acyl-CoA synthetase long chain family member 5	4.721	3.93E-19
**ACSS3**	acyl-CoA synthetase short chain family member 3	-1.628	0.00000828
**ACTA2**	actin alpha 2, smooth muscle	1.784	9.07E-13
**ACTG1P19**	actin gamma 1 pseudogene 19	-2.781	0.0000283
**ACTG2**	actin gamma 2, smooth muscle	-7.959	1.86E-14
**ADAM22**	ADAM metallopeptidase domain 22	1.902	0.000929
**ADAMTS10**	ADAM metallopeptidase with thrombospondin type 1 motif 10	2.726	0.0000112
**ADAMTS12**	ADAM metallopeptidase with thrombospondin type 1 motif 12	2.111	7.73E-21
**ADAMTS14**	ADAM metallopeptidase with thrombospondin type 1 motif 14	7.924	2.08E-16
**ADAMTS17**	ADAM metallopeptidase with thrombospondin type 1 motif 17	6.937	0.000000166
**ADAMTS2**	ADAM metallopeptidase with thrombospondin type 1 motif 2	55.763	5.74E-47
**ADCY1**	adenylate cyclase 1	4.659	0.00000345
**ADGRG1**	adhesion G protein-coupled receptor G1	1.776	1.13E-27
**ADGRG6**	adhesion G protein-coupled receptor G6	1.614	0.0000069
**ADRA1B**	adrenoceptor alpha 1B	-2.075	2.16E-19
**ADRA1D**	adrenoceptor alpha 1D	-3.194	5.93E-29
**ADRB2**	adrenoceptor beta 2	3.608	2.78E-11
**AFAP1L2**	actin filament associated protein 1 like 2	4.946	4.21E-34
**AFF3**	AF4/FMR2 family member 3	3.108	2.2E-21

**AGMAT**	agmatinase	-6.154	0.0000249
**AGTRAP**	angiotensin II receptor associated protein	2.354	4.42E-28
**AHNAK**	AHNAK nucleoprotein	1.597	2.68E-14
**AHNAK2**	AHNAK nucleoprotein 2	1.983	2.3E-29
**AHRR**	aryl-hydrocarbon receptor repressor	1.81	0.000214
**AJAP1**	adherens junctions associated protein 1	-1.892	0.000267
**AK5**	adenylate kinase 5	-3.62	1.28E-50
**AKAP6**	A-kinase anchoring protein 6	-4.078	2.49E-90
**AKR1C1/AKR1C2**	aldo-keto reductase family 1 member C2	5.096	0.0000729
**AKR1C1/AKR1C2**	aldo-keto reductase family 1 member C2	4.024	7.95E-49
**AKR1C3**	aldo-keto reductase family 1 member C3	1.801	4.2E-10
**ALDH1A3**	aldehyde dehydrogenase 1 family member A3	-2.212	2.04E-73
**ALDH1L2**	aldehyde dehydrogenase 1 family member L2	-2.141	0.000399
**ALK**	ALK receptor tyrosine kinase	-3.611	7.55E-17
**ALX4**	ALX homeobox 4	10.918	1.07E-30
**AMOT**	angiomotin	-1.883	1.14E-27
**AMOTL2**	angiomotin like 2	-2.151	7.65E-40
**ANGPTL4**	angiopoietin like 4	6.849	1.41E-21
**ANK1**	ankyrin 1	-4.228	1.76E-39
**ANKRD1**	ankyrin repeat domain 1	-1.892	5.94E-17
**ANKRD18DP**	ankyrin repeat domain 18D, pseudogene	-10.186	0.000109
**ANKRD30B**	ankyrin repeat domain 30B	-3.413	0.00000791
**ANKS1B**	ankyrin repeat and sterile alpha motif domain containing 1B	-5.821	1.21E-29
**ANTXR2**	ANTXR cell adhesion molecule 2	1.735	1.61E-23
**AOC2**	amine oxidase copper containing 2	1.822	0.00000216
**AOX1**	aldehyde oxidase 1	2.29	1.66E-19
**AP3B2**	adaptor related protein complex 3 subunit beta 2	2.574	3.58E-10
**APCDD1**	APC down-regulated 1	-1.981	9.45E-11
**APCDD1L**	APC down-regulated 1 like	3.046	8.92E-91
**APLN**	apelin	-1.897	0.000000293
**APOBEC3F**	apolipoprotein B mRNA editing enzyme catalytic subunit 3F	1.831	0.0000216
**APOL1**	apolipoprotein L1	1.932	4.98E-18
**APOL6**	apolipoprotein L6	2.056	8.18E-31
**ARAP2**	ArfGAP with RhoGAP domain, ankyrin repeat and PH domain 2	2.024	0.000469
**ARG2**	arginase 2	2.982	1.1E-17
**ARHGAP24**	Rho GTPase activating protein 24	24.153	6.13E-36
**ARHGAP25**	Rho GTPase activating protein 25	1.913	0.000237

**ARHGAP36**	Rho GTPase activating protein 36	-5.717	9E-11
**ARHGAP4**	Rho GTPase activating protein 4	2.338	0.00107
**ARHGAP42**	Rho GTPase activating protein 42	1.756	4.4E-10
**ARHGAP44**	Rho GTPase activating protein 44	6.028	0.00000046
**ARHGAP45**	Rho GTPase activating protein 45	-4.917	1.02E-13
**ARHGAP5-AS1**	ARHGAP5 antisense RNA 1 (head to head)	-2.443	3.1E-10
**ARNT2**	aryl hydrocarbon receptor nuclear translocator 2	-2.201	4.78E-46
**ARRB1**	arrestin beta 1	2.74	3.02E-17
**ASIC1**	acid sensing ion channel subunit 1	1.884	4.82E-10
**ASIC3**	acid sensing ion channel subunit 3	2.278	7.88E-10
**ASPHD1**	aspartate beta-hydroxylase domain containing 1	2.566	5.5E-09
**ASS1**	argininosuccinate synthase 1	2.585	3.66E-10
**ATOH8**	atonal bHLH transcription factor 8	-3.426	2.2E-10
**ATP10A**	ATPase phospholipid transporting 10A (putative)	3.379	1.69E-24
**ATP11A**	ATPase phospholipid transporting 11A	2.026	4.98E-32
**ATP2A1**	ATPase sarcoplasmic/endoplasmic reticulum Ca2+ transporting 1	1.984	0.000999
**ATP2B1**	ATPase plasma membrane Ca2+ transporting 1	1.599	1.43E-17
**ATP6V1C2**	ATPase H+ transporting V1 subunit C2	-2.653	3.93E-10
**ATP8A1**	ATPase phospholipid transporting 8A1	4.495	0.00000661
**ATRNL1**	attractin like 1	1.647	0.0000071
**AZGP1**	alpha-2-glycoprotein 1, zinc-binding	-7.972	7.84E-140
**B3GALT5**	beta-1,3-galactosyltransferase 5	3.147	7.43E-09
**B4GALNT3**	beta-1,4-N-acetyl-galactosaminyltransferase 3	-1.977	7.8E-23
**BASP1**	brain abundant membrane attached signal protein 1	35.633	8.34E-142
**BATF3**	basic leucine zipper ATF-like transcription factor 3	-1.889	0.00000313
**BBOX1-AS1**	BBOX1 antisense RNA 1	3.258	0.000132
**BCAR3**	BCAR3 adaptor protein, NSP family member	1.87	4.25E-34
**BCAT1**	branched chain amino acid transaminase 1	-1.565	1.62E-14
**BCHE**	butyrylcholinesterase	2.344	0.000122
**BCL2L11**	BCL2 like 11	2.262	2.49E-34
**BDH1**	3-hydroxybutyrate dehydrogenase 1	-1.635	2.91E-09
**BDH2**	3-hydroxybutyrate dehydrogenase 2	1.993	0.000161
**BEND4**	BEN domain containing 4	6.744	2.75E-18
**BEND5**	BEN domain containing 5	-3.789	0.000369
**BEX1**	brain expressed X-linked 1	-2.499	0.014
**BHLHE40**	basic helix-loop-helix family member e40	1.798	3.36E-31
**BHLHE41**	basic helix-loop-helix family member e41	2.204	2.43E-33

**BMP4**	bone morphogenetic protein 4	13.466	2.1E-16
**BNIP3L**	BCL2 interacting protein 3 like	1.575	3.03E-15
**BOC**	BOC cell adhesion associated, oncogene regulated	1.582	7.94E-12
**BRINP1**	BMP/retinoic acid inducible neural specific 1	122.851	1.73E-115
**BRSK2**	BR serine/threonine kinase 2	4.742	1.3E-09
**BST2**	bone marrow stromal cell antigen 2	3.008	0.000132
**BTBD11**	BTB domain containing 11	19.687	2.12E-42
**BTBD19**	BTB domain containing 19	1.641	9.37E-08
**BTN3A1**	butyrophilin subfamily 3 member A1	1.934	1.78E-15
**BTN3A3**	butyrophilin subfamily 3 member A3	1.796	6.28E-09
**BTNL8**	butyrophilin like 8	5.792	1.21E-08
**C10orf90**	chromosome 10 open reading frame 90	2.127	1.12E-09
**C11orf80**	chromosome 11 open reading frame 80	1.775	4.02E-09
**C11orf87**	chromosome 11 open reading frame 87	-6.448	0.000000851
**C16orf74**	chromosome 16 open reading frame 74	1.96	2.7E-09
**C19orf71**	chromosome 19 open reading frame 71	2.888	0.00993
**C1QTNF6**	C1q and TNF related 6	1.822	0.000155
**C1R**	complement C1r	2.349	8.7E-18
**C1RL**	complement C1r subcomponent like	2.109	3.68E-11
**C1S**	complement C1s	4.187	5.35E-39
**C20orf204**	chromosome 20 open reading frame 204	1.785	0.000187
**C3**	complement C3	8.149	9.18E-33
**C3AR1**	complement C3a receptor 1	2.708	0.00015
**C3orf14**	chromosome 3 open reading frame 14	-1.576	2.49E-10
**C3orf70**	chromosome 3 open reading frame 70	5.441	9.12E-13
**C4orf19**	chromosome 4 open reading frame 19	2.523	6.08E-26
**C5orf46**	chromosome 5 open reading frame 46	-3.031	2.41E-63
**CA11**	carbonic anhydrase 11	2.021	0.000000021
**CA12**	carbonic anhydrase 12	1.858	0.000000114
**CA8**	carbonic anhydrase 8	18.419	3.47E-34
**CACNA1A**	calcium voltage-gated channel subunit alpha1 A	8.839	2.67E-08
**CACNA1E**	calcium voltage-gated channel subunit alpha1 E	5.434	0.000000298
**CACNA2D3**	calcium voltage-gated channel auxiliary subunit alpha2delta 3	2.594	0.000000943
**CACNG4**	calcium voltage-gated channel auxiliary subunit gamma 4	2.74	1.18E-36
**CALB1**	calbindin 1	12.998	8.21E-08
**CALCRL**	calcitonin receptor like receptor	1.813	0.000799
**CALHM5**	calcium homeostasis modulator family member 5	1.947	7.53E-10

**CAMK2N1**	calcium/calmodulin dependent protein kinase II inhibitor 1	2.287	1.89E-24
**CAND2**	cullin associated and neddylation dissociated 2 (putative)	-7.143	3.63E-16
**CARD6**	caspase recruitment domain family member 6	1.836	1.52E-14
**CASP1**	caspase 1	1.686	0.000112
**CBS/CBSL**	cystathionine beta-synthase	-1.611	0.00000209
**CC2D2A**	coiled-coil and C2 domain containing 2A	-1.702	6.11E-16
**CCDC102B**	coiled-coil domain containing 102B	-2.558	9.03E-10
**CCDC106**	coiled-coil domain containing 106	-2.377	5.27E-15
**CCDC13**	coiled-coil domain containing 13	-4.96	0.0000587
**CCDC144A**	coiled-coil domain containing 144A	-3.939	1.25E-14
**CCDC148**	coiled-coil domain containing 148	-2.86	0.0015
**CCDC160**	coiled-coil domain containing 160	-2.429	0.000428
**CCDC184**	coiled-coil domain containing 184	-2.426	5.89E-10
**CCDC61**	coiled-coil domain containing 61	-1.726	5.33E-09
**CCDC80**	coiled-coil domain containing 80	1.6	5.01E-23
**CCN2**	cellular communication network factor 2	-1.719	1.31E-41
**CCN3**	cellular communication network factor 3	8.762	0.000000414
**CD101**	CD101 molecule	2.039	0.000742
**CD180**	CD180 molecule	6.58	3.56E-131
**CD200**	CD200 molecule	-3.566	4.24E-24
**CD274**	CD274 molecule	-1.775	4.28E-21
**CD300C**	CD300c molecule	85.504	2.01E-16
**CD33**	CD33 molecule	31.807	4.48E-182
**CD55**	CD55 molecule (Cromer blood group)	6.505	7.89E-34
**CD82**	CD82 molecule	3.469	5.08E-106
**CD93**	CD93 molecule	5.317	4.73E-13
**CDH6**	cadherin 6	-5.879	4.13E-90
**CDKN1A**	cyclin dependent kinase inhibitor 1A	2.463	1.04E-50
**CDON**	cell adhesion associated, oncogene regulated	1.796	7.47E-29
**CDYL2**	chromodomain Y like 2	7.753	5.36E-81
**CEBPD**	CCAAT enhancer binding protein delta	1.822	0.0016
**CECR2**	CECR2 histone acetyl-lysine reader	16.564	1.45E-10
**CECR7**	cat eye syndrome chromosome region, candidate 7	2.208	0.0000015
**CELSR2**	cadherin EGF LAG seven-pass G-type receptor 2	-1.773	6.12E-21
**CEMIP**	cell migration inducing hyaluronidase 1	2.056	3.7E-15
**CEP41**	centrosomal protein 41	-1.565	8.76E-12
**CERS1**	ceramide synthase 1	2.928	0.00041

**CERS4**	ceramide synthase 4	-1.613	0.0000456
**CES1P1**	carboxylesterase 1 pseudogene 1	80.492	5.08E-26
**CES1P2**	carboxylesterase 1 pseudogene 2	135.627	2.91E-42
**CFAP45**	cilia and flagella associated protein 45	1.909	0.0000193
**CFI**	complement factor I	1.559	2.36E-16
**CGA**	glycoprotein hormones, alpha polypeptide	-4.097	6.74E-10
**CGNL1**	cingulin like 1	-1.576	5.76E-21
**CHAF1A**	chromatin assembly factor 1 subunit A	-1.544	2.21E-16
**CHFR**	checkpoint with forkhead and ring finger domains	-2.15	8.44E-14
**CHI3L1**	chitinase 3 like 1	106.661	0
**CHI3L2**	chitinase 3 like 2	2.813	2.79E-11
**CHST1**	carbohydrate sulfotransferase 1	1.606	0.0000284
**CHST11**	carbohydrate sulfotransferase 11	2.246	9.43E-38
**CHST15**	carbohydrate sulfotransferase 15	12.731	7.37E-123
**CHSY3**	chondroitin sulfate synthase 3	-5.651	9.74E-20
**CIART**	circadian associated repressor of transcription	2.369	4.99E-18
**CIB2**	calcium and integrin binding family member 2	-1.707	0.0000362
**CKB**	creatine kinase B	-2.291	1.64E-13
**CLCA2**	chloride channel accessory 2	7.544	8.21E-17
**CLDN1**	claudin 1	2.41	3E-19
**CLDN15**	claudin 15	1.571	8.86E-08
**CLU**	clusterin	2.3	3.06E-31
**CMPK2**	cytidine/uridine monophosphate kinase 2	4.407	0.000000933
**CMTM8**	CKLF like MARVEL transmembrane domain containing 8	-2.096	1.11E-17
**CNIH2**	cornichon family AMPA receptor auxiliary protein 2	-2.894	0.00000892
**CNR1**	cannabinoid receptor 1	-4.256	4.75E-127
**CNTNAP2**	contactin associated protein like 2	4.58	5.84E-22
**COBL**	cordon-bleu WH2 repeat protein	16.452	2.48E-21
**COBLL1**	cordon-bleu WH2 repeat protein like 1	1.938	3.96E-30
**COL11A1**	collagen type XI alpha 1 chain	-1.771	3.56E-30
**COL13A1**	collagen type XIII alpha 1 chain	1.631	8.38E-10
**COL17A1**	collagen type XVII alpha 1 chain	3.338	2.25E-76
**COL25A1**	collagen type XXV alpha 1 chain	-2.458	0.000249
**COL8A1**	collagen type VIII alpha 1 chain	-1.802	9.58E-16
**COL8A2**	collagen type VIII alpha 2 chain	2.848	0.00000237
**COL9A2**	collagen type IX alpha 2 chain	-1.965	0.000000012
**COL9A3**	collagen type IX alpha 3 chain	-5.761	5.53E-20

**COLEC12**	collectin subfamily member 12	9.503	1.63E-18
**CORO2B**	coronin 2B	-1.782	8.28E-15
**CP**	ceruloplasmin	3.285	5.87E-105
**CPA4**	carboxypeptidase A4	1.705	9.84E-28
**CPA6**	carboxypeptidase A6	-1.541	3.39E-08
**CPE**	carboxypeptidase E	5.737	0.0000189
**CPEB1**	cytoplasmic polyadenylation element binding protein 1	2.093	2.03E-09
**CPM**	carboxypeptidase M	-3.12	0.0000149
**CPT1C**	carnitine palmitoyltransferase 1C	-2.2	6.14E-18
**CPVL**	carboxypeptidase vitellogenic like	2.028	0.000101
**CRACD**	capping protein inhibiting regulator of actin dynamics	-1.858	4.96E-09
**CRISPLD1**	cysteine rich secretory protein LCCL domain containing 1	2.745	1.95E-28
**CRLF1**	cytokine receptor like factor 1	3.335	0.00000259
**CRMP1**	collapsin response mediator protein 1	2.615	0.00000184
**CRY2**	cryptochrome circadian regulator 2	1.628	3.44E-11
**CRYAB**	crystallin alpha B	-3.99	2.82E-91
**CSAG1**	chondrosarcoma associated gene 1	3.737	0.000000263
**CSF1**	colony stimulating factor 1	1.911	2.78E-32
**CSF2**	colony stimulating factor 2	3.184	3.28E-09
**CSGALNACT1**	chondroitin sulfate N-acetylgalactosaminyltransferase 1	2.787	1.25E-08
**CSPG4P11**	chondroitin sulfate proteoglycan 4 pseudogene 11	-1.831	0.000948
**CSRNP3**	cysteine and serine rich nuclear protein 3	3.155	0.0000504
**CTA_384D836**		-1.653	0.000162
**CTA_390C1010**		2.389	0.0000384
**CTB_78F12**		1.696	0.0000592
**CTBS**	chitobiase	1.815	5.49E-16
**CTC_353G131**		-11.197	0.0000077
**CTD_2017D111**		1.793	0.0000183
**CTD_2611O126**		3.748	0.00017
**CTNNA2**	catenin alpha 2	-3.887	1.07E-48
**CTNND2**	catenin delta 2	-2.368	2.61E-26
**CTSH**	cathepsin H	1.948	7.06E-29
**CTSS**	cathepsin S	2.789	7.73E-22
**CXADR**	CXADR Ig-like cell adhesion molecule	1.947	1.92E-19
**CXCL12**	C-X-C motif chemokine ligand 12	6.535	1.97E-39
**CYB5A**	cytochrome b5 type A	-1.577	1.09E-11
**CYB5R2**	cytochrome b5 reductase 2	-1.673	2.75E-09

**CYBRD1**	cytochrome b reductase 1	1.579	1.66E-20
**CYP27C1**	cytochrome P450 family 27 subfamily C member 1	2.644	7.21E-17
**CYP2C8**	cytochrome P450 family 2 subfamily C member 8	4.168	0.0000372
**CYP2R1**	cytochrome P450 family 2 subfamily R member 1	3.227	0.0000858
**CYP46A1**	cytochrome P450 family 46 subfamily A member 1	-1.933	0.000000572
**CYTL1**	cytokine like 1	-12.183	1.62E-17
**DAAM2**	dishevelled associated activator of morphogenesis 2	2.688	0.000144
**DAB1**	DAB adaptor protein 1	-2.113	0.000701
**DARS-AS1**	DARS antisense RNA 1	2.602	0.00014
**DBP**	D-box binding PAR bZIP transcription factor	1.887	1.57E-08
**DDIT4**	DNA damage inducible transcript 4	1.64	4.43E-09
**DENND2D**	DENN domain containing 2D	-3.804	0.000111
**DEPP1**	DEPP1 autophagy regulator	2.264	1.51E-16
**DGAT2**	diacylglycerol O-acyltransferase 2	2.068	2.15E-15
**DGCR5**	DiGeorge syndrome critical region gene 5	2.083	0.0000354
**DGKE**	diacylglycerol kinase epsilon	1.736	0.0000241
**DGKI**	diacylglycerol kinase iota	1.564	7.02E-08
**DHODH**	dihydroorotate dehydrogenase (quinone)	-1.538	9.1E-13
**DHRS2**	dehydrogenase/reductase 2	3.351	1.67E-17
**DHRS9**	dehydrogenase/reductase 9	-4.562	2.47E-14
**DIO2**	iodothyronine deiodinase 2	-2.407	1.51E-27
**DIRAS1**	DIRAS family GTPase 1	-5.899	4.58E-26
**DIRAS3**	DIRAS family GTPase 3	-1.689	2.33E-17
**DISP2**	dispatched RND transporter family member 2	1.897	0.000375
**DLC1**	DLC1 Rho GTPase activating protein	2.454	5.33E-33
**DLL1**	delta like canonical Notch ligand 1	3.502	1.33E-14
**DMBT1**	deleted in malignant brain tumors 1	4.286	0.000000135
**DMBX1**	diencephalon/mesencephalon homeobox 1	3.814	3.43E-34
**DMRTA1**	DMRT like family A1	1.705	0.00000353
**DNAJB9**	DnaJ heat shock protein family (Hsp40) member B9	1.562	0.00000025
**DNER**	delta/notch like EGF repeat containing	1.602	2.83E-22
**DNHD1**	dynein heavy chain domain 1	1.939	7.94E-09
**DNM1**	dynamin 1	1.585	1.16E-17
**DNM3**	dynamin 3	-2.287	8.54E-26
**DNPH1**	2'-deoxynucleoside 5'-phosphate N-hydrolase 1	-1.659	0.000042
**DOCK3**	dedicator of cytokinesis 3	1.69	0.000000233
**DOCK4**	dedicator of cytokinesis 4	2.152	8.42E-37

**DOK5**	docking protein 5	-9.578	1.68E-54
**DPYSL5**	dihydropyrimidinase like 5	8.562	1.5E-149
**DRD2**	dopamine receptor D2	1.596	2.41E-17
**DSE**	dermatan sulfate epimerase	2.15	1.46E-24
**DSP**	desmoplakin	4.858	6.88E-14
**DTX3**	deltex E3 ubiquitin ligase 3	-2.117	1.4E-20
**DUSP1**	dual specificity phosphatase 1	1.538	7.83E-12
**DUSP10**	dual specificity phosphatase 10	1.904	5.14E-19
**DUSP5**	dual specificity phosphatase 5	2.144	3.6E-28
**DUXAP10**	double homeobox A pseudogene 10	7	3.89E-24
**DUXAP8**	double homeobox A pseudogene 8	14.656	3.15E-31
**DUXAP9**	double homeobox A pseudogene 9	7.291	3.17E-16
**DYRK3**	dual specificity tyrosine phosphorylation regulated kinase 3	1.856	0.00208
**E2F8**	E2F transcription factor 8	-1.612	8.19E-14
**EBF1**	EBF transcription factor 1	1.756	0.0000748
**ECSCR**	endothelial cell surface expressed chemotaxis and apoptosis regulator	5.299	5.33E-13
**EDIL3**	EGF like repeats and discoidin domains 3	2.962	5.07E-58
**EDIL3-DT**		2.544	0.0000264
**EDN1**	endothelin 1	-2.198	4.08E-37
**EDNRB**	endothelin receptor type B	-1.653	1.8E-10
**EEF1A2**	eukaryotic translation elongation factor 1 alpha 2	2.135	1.18E-22
**EEFSEC**	eukaryotic elongation factor, selenocysteine-tRNA specific	-2.121	4.63E-17
**EFEMP2**	EGF containing fibulin extracellular matrix protein 2	-3.334	1.67E-21
**EFNA1**	ephrin A1	2.769	1.33E-12
**EFNA4**	ephrin A4	1.642	0.0000207
**EHD3**	EH domain containing 3	2.26	3.85E-28
**ELFN2**	extracellular leucine rich repeat and fibronectin type III domain containing 2	2.166	4.57E-30
**ELMO1**	engulfment and cell motility 1	1.675	1.11E-12
**ELOVL2**	ELOVL fatty acid elongase 2	-2.215	0.000000304
**ELOVL7**	ELOVL fatty acid elongase 7	1.717	2.17E-08
**EN1**	engrailed homeobox 1	-1.664	4.09E-08
**ENPP2**	ectonucleotide pyrophosphatase/phosphodiesterase 2	8.436	9.52E-10
**ENPP5**	ectonucleotide pyrophosphatase/phosphodiesterase family member 5	1.806	0.000997
**EPAS1**	endothelial PAS domain protein 1	2.353	4.19E-62
**EPB41L4A**	erythrocyte membrane protein band 4.1 like 4A	2.263	2.06E-21
**EPB41L4B**	erythrocyte membrane protein band 4.1 like 4B	2.883	4.79E-37
**EPHA3**	EPH receptor A3	-2.238	3.18E-61

**EPHA4**	EPH receptor A4	-1.887	5E-10
**EPHA5**	EPH receptor A5	3.216	2.23E-41
**EPHB2**	EPH receptor B2	2.672	7.39E-82
**EPHX1**	epoxide hydrolase 1	1.816	2.84E-24
**EPHX4**	epoxide hydrolase 4	1.72	0.00000156
**EPSTI1**	epithelial stromal interaction 1	-1.775	0.00000341
**ERAP1**	endoplasmic reticulum aminopeptidase 1	1.659	1.85E-22
**ERAP2**	endoplasmic reticulum aminopeptidase 2	2.21	3.84E-54
**ERCC2**	ERCC excision repair 2, TFIIH core complex helicase subunit	-1.974	2.29E-29
**ERFE**	erythroferrone	2.313	2.14E-24
**ERICH3**	glutamate rich 3	-8.581	1.45E-09
**ERMP1**	endoplasmic reticulum metallopeptidase 1	1.988	2.38E-25
**ERP29**	endoplasmic reticulum protein 29	1.608	3.1E-21
**ETV1**	ETS variant transcription factor 1	1.885	1.44E-13
**EVC**	EvC ciliary complex subunit 1	-1.57	8.82E-15
**EVI2A**	ecotropic viral integration site 2A	1.999	3.88E-10
**EXTL1**	exostosin like glycosyltransferase 1	-9.48	5.62E-16
**EYA2**	EYA transcriptional coactivator and phosphatase 2	2.641	0.000207
**F11R**	F11 receptor	-2.053	6.97E-15
**F2RL2**	coagulation factor II thrombin receptor like 2	1.854	3.48E-13
**F3**	coagulation factor III, tissue factor	-1.814	6.8E-26
**FABP7**	fatty acid binding protein 7	-1.763	7.25E-25
**FAM106C**	family with sequence similarity 106 member C	-3.448	0.000144
**FAM131A**	family with sequence similarity 131 member A	-1.968	1.98E-15
**FAM131B**	family with sequence similarity 131 member B	1.994	0.0000479
**FAM13A-AS1**	FAM13A antisense RNA 1	1.87	0.000559
**FAM160A1**	family with sequence similarity 160 member A1	1.65	0.000000769
**FAM166C**	family with sequence similarity 166 member C	2.653	0.000409
**FAM167A**	family with sequence similarity 167 member A	3.374	0.0000133
**FAM171B**	family with sequence similarity 171 member B	1.583	4.19E-08
**FAM189A1**	family with sequence similarity 189 member A1	16.445	1.06E-95
**FAM20C**	FAM20C golgi associated secretory pathway kinase	1.6	8.45E-12
**FAM229B**	family with sequence similarity 229 member B	2.006	0.000000011
**FAM89A**	family with sequence similarity 89 member A	3.648	4.36E-24
**FAP**	fibroblast activation protein alpha	3.139	1.05E-26
**FAS**	Fas cell surface death receptor	1.58	1.88E-11
**FAT2**	FAT atypical cadherin 2	55.643	1.27E-52

**FAT3**	FAT atypical cadherin 3	-2.128	1.11E-29
**FBLN1**	fibulin 1	1.808	1.06E-19
**FBLN5**	fibulin 5	8.105	3.81E-10
**FBN1**	fibrillin 1	1.662	7.59E-24
**FBXO2**	F-box protein 2	4.151	0.000000687
**FBXO4**	F-box protein 4	-1.595	2.18E-08
**FCGRT**	Fc fragment of IgG receptor and transporter	-2.166	1.01E-11
**FCRLA**	Fc receptor like A	3.263	0.0000468
**FENDRR**	FOXF1 adjacent non-coding developmental regulatory RNA	31.188	6.63E-08
**FER**	FER tyrosine kinase	-1.906	1.42E-19
**FER1L4**	fer-1 like family member 4 (pseudogene)	3.348	4.31E-25
**FERMT3**	fermitin family member 3	-2.183	0.0000879
**FEZF1**	FEZ family zinc finger 1	-3.705	0.00000267
**FEZF1-AS1**	FEZF1 antisense RNA 1	-4.052	1.14E-19
**FGD6**	FYVE, RhoGEF and PH domain containing 6	2.125	1.33E-11
**FGFBP2**	fibroblast growth factor binding protein 2	-18.816	0.000000114
**FHL1**	four and a half LIM domains 1	1.569	2.12E-17
**FIBCD1**	fibrinogen C domain containing 1	3	4.69E-15
**FIGN**	fidgetin, microtubule severing factor	-1.581	1.8E-13
**FILIP1**	filamin A interacting protein 1	-2	5.94E-08
**FKBP10**	FKBP prolyl isomerase 10	-1.61	1.28E-11
**FKBP14**	FKBP prolyl isomerase 14	1.629	2.08E-15
**FLJ22447**	uncharacterized LOC400221	4.887	3.58E-40
**FLRT2**	fibronectin leucine rich transmembrane protein 2	2.523	5.77E-47
**FLRT3**	fibronectin leucine rich transmembrane protein 3	-2.591	4.48E-40
**FMN2**	formin 2	-2.078	2.37E-36
**FMNL1**	formin like 1	3.06	2.09E-77
**FN1**	fibronectin 1	1.697	9.78E-28
**FOSB**	FosB proto-oncogene, AP-1 transcription factor subunit	2.626	1.07E-08
**FOXA1**	forkhead box A1	-1.943	1.24E-13
**FOXF1**	forkhead box F1	9.586	7.82E-33
**FOXF2**	forkhead box F2	2.32	2.83E-14
**FOXG1**	forkhead box G1	-1.723	2.56E-10
**FOXQ1**	forkhead box Q1	3.167	1.43E-13
**FOXRED2**	FAD dependent oxidoreductase domain containing 2	-1.55	6.58E-14
**FOXS1**	forkhead box S1	9.543	5.26E-08
**FPR1**	formyl peptide receptor 1	9.942	2.2E-53

**FRAS1**	Fraser extracellular matrix complex subunit 1	2.04	0.000294
**FRMD3**	FERM domain containing 3	-1.778	1.17E-19
**FSCN1**	fascin actin-bundling protein 1	3.05	2.99E-26
**FSCN1P1**	fascin actin-bundling protein 1 pseudogene 1	4.125	0.0000015
**FSIP1**	fibrous sheath interacting protein 1	-1.808	0.000116
**GABBR2**	gamma-aminobutyric acid type B receptor subunit 2	-1.682	0.00000111
**GABRA3**	gamma-aminobutyric acid type A receptor alpha3 subunit	2.966	0.00000669
**GABRE**	gamma-aminobutyric acid type A receptor epsilon subunit	6.304	3.84E-63
**GACAT2**	gastric cancer associated transcript 2	-2.708	0.0000538
**GAL3ST4**	galactose-3-O-sulfotransferase 4	-2.252	0.0000359
**GALNT12**	polypeptide N-acetylgalactosaminyltransferase 12	-2.864	0.00119
**GALNT18**	polypeptide N-acetylgalactosaminyltransferase 18	11.63	1.76E-13
**GALNT6**	polypeptide N-acetylgalactosaminyltransferase 6	-3.171	3.65E-08
**GAP43**	growth associated protein 43	-2.792	2.47E-37
**GAREM2**	GRB2 associated regulator of MAPK1 subtype 2	-3.312	2.31E-14
**GAS1**	growth arrest specific 1	2.653	3.98E-16
**GAS6-DT**	GAS6 divergent transcript	2.79	4.33E-09
**GAS7**	growth arrest specific 7	2.09	5.69E-34
**GATA2**	GATA binding protein 2	-1.565	0.0000426
**GATA3**	GATA binding protein 3	-1.869	3.06E-18
**GBP4**	guanylate binding protein 4	4.338	7.62E-26
**GCNT1**	glucosaminyl (N-acetyl) transferase 1	1.863	1.68E-30
**GDPD5**	glycerophosphodiester phosphodiesterase domain containing 5	2.645	3.1E-17
**GFPT2**	glutamine-fructose-6-phosphate transaminase 2	1.89	8.84E-42
**GGT5**	gamma-glutamyltransferase 5	4.015	0.000145
**GIMAP2**	GTPase, IMAP family member 2	2.521	6.06E-12
**GIMAP5**	GTPase, IMAP family member 5	36.918	2.29E-12
**GIMAP6**	GTPase, IMAP family member 6	16.724	1.1E-63
**GJA1**	gap junction protein alpha 1	1.752	3.97E-37
**GJA3**	gap junction protein alpha 3	2.378	1.97E-11
**GJB2**	gap junction protein beta 2	2.138	5.13E-51
**GLDC**	glycine decarboxylase	2.071	1.16E-17
**GLDN**	gliomedin	-6.874	8.27E-17
**GLIS1**	GLIS family zinc finger 1	-2.254	6.55E-08
**GLP2R**	glucagon like peptide 2 receptor	12.327	2.63E-09
**GLT8D2**	glycosyltransferase 8 domain containing 2	1.597	0.000000135
**GLYATL1**	glycine-N-acyltransferase like 1	2.351	0.000962

**GNAL**	G protein subunit alpha L	5.46	2.26E-25
**GNAO1**	G protein subunit alpha o1	2.712	1.68E-24
**GNG7**	G protein subunit gamma 7	-3.954	5.1E-09
**GOPC**	golgi associated PDZ and coiled-coil motif containing	1.625	2.46E-19
**GPC4**	glypican 4	-1.591	6.39E-20
**GPM6A**	glycoprotein M6A	-6.968	2.93E-213
**GPM6B**	glycoprotein M6B	-1.915	2.23E-26
**GPR1**	G protein-coupled receptor 1	3.746	0.00000465
**GPR156**	G protein-coupled receptor 156	-2.638	2.02E-08
**GPR160**	G protein-coupled receptor 160	1.923	0.00000297
**GPR63**	G protein-coupled receptor 63	2.205	5.08E-11
**GPRC5B**	G protein-coupled receptor class C group 5 member B	-1.988	1.22E-17
**GPRC5C**	G protein-coupled receptor class C group 5 member C	15.858	0.000000102
**GPX3**	glutathione peroxidase 3	-2.987	9.17E-12
**GRAMD1B**	GRAM domain containing 1B	1.758	0.000000263
**GRIK4**	glutamate ionotropic receptor kainate type subunit 4	2.258	2.41E-11
**GRIN2A**	glutamate ionotropic receptor NMDA type subunit 2A	3.569	1.16E-12
**GRIP1**	glutamate receptor interacting protein 1	1.73	0.000000472
**GRK5**	G protein-coupled receptor kinase 5	1.568	2.1E-11
**GRPR**	gastrin releasing peptide receptor	-3.668	9.52E-25
**GSDMC**	gasdermin C	5.254	4.43E-11
**GSN**	gelsolin	-1.654	1.25E-13
**GULP1**	GULP PTB domain containing engulfment adaptor 1	-3.132	2.59E-56
**GYG2**	glycogenin 2	-3.188	5.56E-13
**H1-2**	H1.2 linker histone, cluster member	-1.618	2.57E-08
**H1FX-AS1**	H1FX antisense RNA 1	-1.786	0.000147
**H2AC6**	H2A clustered histone 6	-1.967	3.62E-12
**H3C4**	H3 clustered histone 4	-2.702	0.00501
**HAS3**	hyaluronan synthase 3	3.73	4.34E-100
**HBEGF**	heparin binding EGF like growth factor	1.54	1.97E-13
**HCFC1R1**	host cell factor C1 regulator 1	1.604	1.67E-10
**HCN2**	hyperpolarization activated cyclic nucleotide gated potassium and sodium channel 2	2.797	3.76E-19
**HDAC9**	histone deacetylase 9	-1.874	1.02E-30
**HEPH**	hephaestin	1.657	1.78E-10
**HERC5**	HECT and RLD domain containing E3 ubiquitin protein ligase 5	1.907	3.14E-19
**HERC6**	HECT and RLD domain containing E3 ubiquitin protein ligase family member 6	1.754	0.000000399
**HES4**	hes family bHLH transcription factor 4	-2.308	0.000202

**HEY2**	hes related family bHLH transcription factor with YRPW motif 2	3.85	4.08E-09
**HHIP**	hedgehog interacting protein	26.124	0.000000186
**HIP1R**	huntingtin interacting protein 1 related	1.554	1.73E-08
**HIVEP2**	HIVEP zinc finger 2	1.89	2.4E-26
**HLA-DPA1**	major histocompatibility complex, class II, DP alpha 1	11.46	3E-21
**HMGN5**	high mobility group nucleosome binding domain 5	8.616	2.14E-53
**HMOX1**	heme oxygenase 1	2.063	8.58E-43
**HOPX**	HOP homeobox	-1.747	0.00014
**HOXA7**	homeobox A7	-2.562	0.0000462
**HPS1**	HPS1 biogenesis of lysosomal organelles complex 3 subunit 1	-1.67	1.66E-10
**HRAT17**	heart tissue-associated transcript 17	-4.25	7.52E-09
**HRH2**	histamine receptor H2	22.904	2.61E-11
**HS3ST1**	heparan sulfate-glucosamine 3-sulfotransferase 1	-2.187	0.000477
**HS3ST3B1**	heparan sulfate-glucosamine 3-sulfotransferase 3B1	1.699	7.8E-16
**HSPA6**	heat shock protein family A (Hsp70) member 6	1.858	0.000000367
**HTRA1**	HtrA serine peptidase 1	1.637	1.22E-20
**ICOSLG/LOC102723996**	inducible T cell costimulator ligand	2.463	2.05E-36
**ID3**	inhibitor of DNA binding 3, HLH protein	-1.602	1.42E-09
**ID4**	inhibitor of DNA binding 4, HLH protein	1.543	9.97E-11
**IDO1**	indoleamine 2,3-dioxygenase 1	5.39	2.38E-16
**IFI44L**	interferon induced protein 44 like	-1.643	7.91E-11
**IFIT1**	interferon induced protein with tetratricopeptide repeats 1	1.868	0.000000132
**IFIT3**	interferon induced protein with tetratricopeptide repeats 3	1.629	3.6E-10
**IFITM10**	interferon induced transmembrane protein 10	1.919	8.72E-21
**IFRD2**	interferon related developmental regulator 2	-1.574	6.25E-09
**IGFBP2**	insulin like growth factor binding protein 2	-1.651	1.1E-12
**IGFBP3**	insulin like growth factor binding protein 3	3.696	3.52E-125
**IGFBP4**	insulin like growth factor binding protein 4	11.214	0
**IGFBP5**	insulin like growth factor binding protein 5	-2.43	1.78E-64
**IGFBP6**	insulin like growth factor binding protein 6	1.699	3.04E-08
**IGSF10**	immunoglobulin superfamily member 10	2.182	0.0000729
**IKBIP**	IKBKB interacting protein	1.589	2.89E-14
**IKZF2**	IKAROS family zinc finger 2	2.064	0.0000183
**IL11**	interleukin 11	2.713	1.05E-38
**IL13RA2**	interleukin 13 receptor subunit alpha 2	2.714	1.73E-60
**IL15**	interleukin 15	1.911	0.00000469
**IL15RA**	interleukin 15 receptor subunit alpha	2.697	4.51E-12

**IL24**	interleukin 24	11.493	0.000000015
**IL27RA**	interleukin 27 receptor subunit alpha	1.964	1.95E-15
**IL4R**	interleukin 4 receptor	5.355	2.26E-51
**IL7**	interleukin 7	2.553	5.47E-10
**IMPDH1P10**	inosine monophosphate dehydrogenase 1 pseudogene 10	-2.311	0.000641
**INAVA**	innate immunity activator	-2.805	0.000121
**INPP1**	inositol polyphosphate-1-phosphatase	-1.551	4.03E-10
**INPP5J**	inositol polyphosphate-5-phosphatase J	-2.114	0.000592
**IQGAP2**	IQ motif containing GTPase activating protein 2	-2.482	2.78E-08
**IRF1**	interferon regulatory factor 1	2.014	6.06E-22
**IRX1**	iroquois homeobox 1	-1.913	0.00000314
**ITGA1**	integrin subunit alpha 1	2.22	6.49E-24
**ITGA11**	integrin subunit alpha 11	1.932	0.00000194
**ITGA2**	integrin subunit alpha 2	2.636	3.65E-71
**ITGA9**	integrin subunit alpha 9	-1.728	1.47E-10
**ITGB2**	integrin subunit beta 2	2.405	0.00000294
**ITGB3**	integrin subunit beta 3	1.576	6.47E-13
**ITGB4**	integrin subunit beta 4	1.548	3.65E-10
**ITGB6**	integrin subunit beta 6	3.69	0.000102
**ITGB8**	integrin subunit beta 8	-1.584	1.01E-15
**ITGBL1**	integrin subunit beta like 1	-2.171	1.12E-21
**JAG2**	jagged canonical Notch ligand 2	5.436	3.04E-17
**JAKMIP3**	Janus kinase and microtubule interacting protein 3	1.586	1.81E-08
**JDP2**	Jun dimerization protein 2	2.568	0.000000059
**JMJD8**	jumonji domain containing 8	-1.64	5.28E-09
**JUP**	junction plakoglobin	-2.291	5.02E-09
**KALRN**	kalirin RhoGEF kinase	1.784	3.52E-12
**KCNAB2**	potassium voltage-gated channel subfamily A regulatory beta subunit 2	3.027	1.04E-48
**KCNB1**	potassium voltage-gated channel subfamily B member 1	18.182	1.5E-31
**KCND3**	potassium voltage-gated channel subfamily D member 3	-9.741	4.78E-26
**KCNH5**	potassium voltage-gated channel subfamily H member 5	-5.17	0.00000209
**KCNIP3**	potassium voltage-gated channel interacting protein 3	2.718	4.02E-19
**KCNIP4**	potassium voltage-gated channel interacting protein 4	-2.48	1.04E-08
**KCNJ2**	potassium inwardly rectifying channel subfamily J member 2	1.66	8.81E-10
**KCNK3**	potassium two pore domain channel subfamily K member 3	3.288	0.00000167
**KCNK5**	potassium two pore domain channel subfamily K member 5	78.236	7.63E-40
**KCNMA1**	potassium calcium-activated channel subfamily M alpha 1	2.784	8.59E-119

**KCNN4**	potassium calcium-activated channel subfamily N member 4	2.002	3.57E-29
**KCTD12**	potassium channel tetramerization domain containing 12	-1.591	8.17E-17
**KCTD15**	potassium channel tetramerization domain containing 15	-2.164	2.66E-44
**KDELR3**	KDEL endoplasmic reticulum protein retention receptor 3	2.249	6.64E-40
**KDM5B**	lysine demethylase 5B	1.675	2.3E-22
**KIAA0319**	KIAA0319	1.699	0.0000179
**KIF17**	kinesin family member 17	-2.513	0.000101
**KIF7**	kinesin family member 7	-1.6	0.000000174
**KIRREL3**	kirre like nephrin family adhesion molecule 3	-1.586	0.000000167
**KITLG**	KIT ligand	2.171	2.84E-29
**KLF17**	Kruppel like factor 17	5.808	1.86E-18
**KLHDC7A**	kelch domain containing 7A	7.596	0.0000237
**KLHL13**	kelch like family member 13	-1.612	5.86E-14
**KRT13**	keratin 13	-3.086	0.00169
**KRT18**	keratin 18	-1.756	1.48E-11
**KRT81**	keratin 81	-1.78	1.36E-11
**KSR1**	kinase suppressor of ras 1	1.763	1.55E-21
**L1CAM**	L1 cell adhesion molecule	4.844	1.93E-99
**LAIR1**	leukocyte associated immunoglobulin like receptor 1	14.254	3.1E-21
**LAMA1**	laminin subunit alpha 1	2.639	0.000093
**LAMA2**	laminin subunit alpha 2	2.611	1.1E-14
**LAMC2**	laminin subunit gamma 2	2.977	2.54E-21
**LAPTM5**	lysosomal protein transmembrane 5	1.929	9.31E-16
**LBH**	LBH regulator of WNT signaling pathway	2.17	0.000000162
**LCP1**	lymphocyte cytosolic protein 1	1.71	7.12E-15
**LDHA**	lactate dehydrogenase A	1.667	2.89E-13
**LGR4**	leucine rich repeat containing G protein-coupled receptor 4	3.779	8.68E-63
**LGR5**	leucine rich repeat containing G protein-coupled receptor 5	-3.714	3.42E-23
**LHFPL4**	LHFPL tetraspan subfamily member 4	3.295	0.0000641
**LIN7A**	lin-7 homolog A, crumbs cell polarity complex component	-2.969	0.00149
**LINC00327**	long intergenic non-protein coding RNA 327	-1.893	0.000236
**LINC00460**	long intergenic non-protein coding RNA 460	-1.81	0.00000666
**LINC00461**	long intergenic non-protein coding RNA 461	-2.049	1.01E-36
**LINC00565**	long intergenic non-protein coding RNA 565	3.33	0.0000132
**LINC00632**	long intergenic non-protein coding RNA 632	-2.134	0.00000176
**LINC00639**	long intergenic non-protein coding RNA 639	-3.302	2.41E-25
**LINC00707**	long intergenic non-protein coding RNA 707	-1.53	8.08E-13

**LINC00963**	long intergenic non-protein coding RNA 963	1.633	3.63E-15
**LINC01111**	long intergenic non-protein coding RNA 1111	-3.11	3.18E-10
**LINC01303**	long intergenic non-protein coding RNA 1303	2.979	9.37E-08
**LINC01503**	long intergenic non-protein coding RNA 1503	2.152	0.00000788
**LINC01551**	long intergenic non-protein coding RNA 1551	-1.748	0.000000147
**LINC02454**	long intergenic non-protein coding RNA 2454	2.163	0.000235
**LINC02535**	long intergenic non-protein coding RNA 2535	1.913	0.0000379
**LINC02547**	long intergenic non-protein coding RNA 2547	2.387	0.000648
**LINC02582**	long intergenic non-protein coding RNA 2582	10.845	0.0000069
**LINC02732**		-3.118	8.02E-17
**LINGO2**	leucine rich repeat and Ig domain containing 2	-4.671	6.03E-08
**LIPE**	lipase E, hormone sensitive type	-2.664	4.79E-11
**LIPG**	lipase G, endothelial type	2.63	0.0000533
**LLGL2**	LLGL scribble cell polarity complex component 2	-3.339	2.3E-15
**LMCD1**	LIM and cysteine rich domains 1	-2.107	1.33E-22
**LMO3**	LIM domain only 3	-2.687	4.14E-45
**LNCAROD**	lncRNA activating regulator of DKK1	-1.769	2.05E-18
**LNCOG**	lncRNA osteogenesis associated	5.653	0.00000919
**LOC100129534**	small nuclear ribonucleoprotein polypeptide N pseudogene	3.424	3.51E-08
**LOC100130283**	uncharacterized LOC100130283	2.185	0.0000899
**LOC100507516**	uncharacterized LOC100507516	-5.124	4.29E-48
**LOC101927533**	uncharacterized LOC101927533	5.909	0.000000504
**LOC101928663**	uncharacterized LOC101928663	2.373	3.44E-20
**LOC102723834**	uncharacterized LOC102723834	1.916	0.000043
**LOC102724814**	uncharacterized LOC102724814	1.671	0.000000487
**LOC105369203**		-2.467	2.98E-28
**LOC105371795**		2.256	0.000000856
**LOC107984895**	uncharacterized LOC107984895	2.228	0.000645
**LOC107984948**		2.913	2.1E-14
**LOC283335**	uncharacterized LOC283335	1.856	0.0000729
**LOC339192**	uncharacterized LOC339192	3.409	6.47E-10
**LOC643201**	centrosomal protein 192kDa pseudogene	3.523	1.53E-44
**LOX**	lysyl oxidase	9.077	1.32E-160
**LOXL1**	lysyl oxidase like 1	2.736	2.92E-17
**LOXL1-AS1**	LOXL1 antisense RNA 1	4.089	4.66E-27
**LOXL4**	lysyl oxidase like 4	-1.776	1.09E-15
**LPAL2**	lipoprotein(a) like 2, pseudogene	10.188	6.68E-09

**LPL**	lipoprotein lipase	-2.39	3.25E-20
**LPXN**	leupaxin	2.047	2.71E-36
**LRAT**	lecithin retinol acyltransferase	-3.106	1.9E-33
**LRATD2**	LRAT domain containing 2	-1.809	6.1E-23
**LRP1**	LDL receptor related protein 1	1.567	1.15E-11
**LRP4-AS1**	LRP4 antisense RNA 1	3.621	0.0000407
**LRRC17**	leucine rich repeat containing 17	6.193	1.96E-60
**LRRC2**	leucine rich repeat containing 2	-2.526	2.34E-21
**LRRC45**	leucine rich repeat containing 45	-1.941	0.000000315
**LRRC8B**	leucine rich repeat containing 8 VRAC subunit B	-2.349	2.9E-36
**LSAMP**	limbic system associated membrane protein	-1.89	0.000000359
**LSMEM1**	leucine rich single-pass membrane protein 1	1.869	0.000379
**LSP1**	lymphocyte specific protein 1	5.478	0.000000169
**LTBP1**	latent transforming growth factor beta binding protein 1	2.53	6.61E-52
**LTBP2**	latent transforming growth factor beta binding protein 2	1.662	2.59E-08
**LYPD1**	LY6/PLAUR domain containing 1	-1.737	2.93E-37
**MAFB**	MAF bZIP transcription factor B	2.972	0.000243
**MAGEA12**	MAGE family member A12	3.893	0.0000242
**MAGEB6**	MAGE family member B6	8.736	0.00000148
**MAGEH1**	MAGE family member H1	7.705	1.44E-66
**MAGI2**	membrane associated guanylate kinase, WW and PDZ domain containing 2	-1.734	1.11E-12
**MAMDC2**	MAM domain containing 2	2.101	4.41E-50
**MAN1A1**	mannosidase alpha class 1A member 1	1.757	2.05E-23
**MAOA**	monoamine oxidase A	4.884	0.00000178
**MAP1A**	microtubule associated protein 1A	1.568	1.37E-18
**MAP3K7CL**	MAP3K7 C-terminal like	2.061	0.000273
**MAP6**	microtubule associated protein 6	-2.76	0.0001
**MARCHF1**	membrane associated ring-CH-type finger 1	-2.174	8.82E-18
**MARCHF10**	membrane associated ring-CH-type finger 10	-2.905	0.00000788
**MAST4**	microtubule associated serine/threonine kinase family member 4	2.215	7.04E-27
**MATN2**	matrilin 2	3.153	6.26E-108
**MATN3**	matrilin 3	-2.846	0.00000142
**MBL1P**	mannose binding lectin 1, pseudogene	5.46	0.00000131
**MBNL3**	muscleblind like splicing regulator 3	-1.572	0.00000665
**MC1R**	melanocortin 1 receptor	2.066	2.06E-08
**MCF2**	MCF.2 cell line derived transforming sequence	-5.678	0.0000211
**MCM5**	minichromosome maintenance complex component 5	-1.858	2.79E-28

**MCTP1**	multiple C2 and transmembrane domain containing 1	2.688	6.78E-27
**MDGA1**	MAM domain containing glycosylphosphatidylinositol anchor 1	2.486	3.48E-48
**MDK**	midkine	-4.537	3.19E-33
**MEF2C**	myocyte enhancer factor 2C	-1.722	2.54E-10
**MEG3**	maternally expressed 3	1.899	4.77E-11
**MEIS2**	Meis homeobox 2	-1.623	2.27E-16
**MEIS3P1**	Meis homeobox 3 pseudogene 1	-4.505	1.19E-21
**MEIS3P2**	Meis homeobox 3 pseudogene 2	-2.907	0.000167
**MELTF**	melanotransferrin	-1.943	1.67E-08
**MEST**	mesoderm specific transcript	2.089	6.43E-47
**MEX3B**	mex-3 RNA binding family member B	1.696	0.000000166
**MGC12916**	uncharacterized protein MGC12916	2.336	9.33E-08
**MGLL**	monoglyceride lipase	2.107	4.07E-53
**MGP**	matrix Gla protein	2.171	3.23E-39
**MGST1**	microsomal glutathione S-transferase 1	1.541	1.79E-21
**MIR100HG**	mir-100-let-7a-2-mir-125b-1 cluster host gene	2.035	4.77E-29
**MIR503HG**	MIR503 host gene	1.963	1.15E-08
**MIRLET7BHG**	MIRLET7B host gene	-1.588	6.55E-08
**MKLN1-AS**	MKLN1 antisense RNA	1.84	0.00034
**MME**	membrane metalloendopeptidase	4.053	1.72E-72
**MMP1**	matrix metallopeptidase 1	9.258	1.88E-08
**MMP14**	matrix metallopeptidase 14	2.842	2.19E-100
**MMP17**	matrix metallopeptidase 17	-3.875	1.58E-09
**MMP2**	matrix metallopeptidase 2	1.663	7.65E-23
**MMP3**	matrix metallopeptidase 3	6.528	6.1E-21
**MMP7**	matrix metallopeptidase 7	2.754	6.64E-38
**MOXD1**	monooxygenase DBH like 1	-2.299	9.44E-71
**MPP2**	membrane palmitoylated protein 2	-2.045	2.89E-16
**MPP4**	membrane palmitoylated protein 4	1.933	1.63E-08
**MPP7**	membrane palmitoylated protein 7	2.112	0.000011
**MROCKI**	MARCKS cis regulating lncRNA promoter of cytokines and inflammation	-3.483	3.81E-31
**MSANTD2**	Myb/SANT DNA binding domain containing 2	-1.692	2.13E-15
**MSX2**	msh homeobox 2	1.804	0.000000333
**MT-ND5**	NADH dehydrogenase, subunit 5 (complex I)	1.533	5.74E-17
**MT-RNR1**	s-rRNA	2.141	7.48E-32
**MT-RNR2**	l-rRNA	2.438	3.38E-90
**MT-TT**	tRNA	2.442	0.000000839

**MT1E**	metallothionein 1E	-2.018	3.57E-16
**MTCL1**	microtubule crosslinking factor 1	-1.794	5.13E-31
**MTMR1**	myotubularin related protein 1	-2.111	1.39E-30
**MTRNR2L1**	MT-RNR2 like 1	2.725	3.41E-15
**MTRNR2L10**	MT-RNR2 like 10	3.155	5.89E-17
**MTRNR2L12**	MT-RNR2 like 12	2.419	5.64E-37
**MTRNR2L2**	MT-RNR2 like 2	2.107	1.31E-18
**MTRNR2L8**	MT-RNR2 like 8	2.166	6.05E-15
**MTSS1**	MTSS I-BAR domain containing 1	12.624	9.27E-67
**MTUS1**	microtubule associated scaffold protein 1	6.961	0.000000487
**MUC1**	mucin 1, cell surface associated	1.92	0.0000115
**MYBL2**	MYB proto-oncogene like 2	-1.58	4.97E-15
**MYD88**	MYD88 innate immune signal transduction adaptor	-1.525	3.33E-14
**MYEOV**	myeloma overexpressed	-2.16	1.42E-23
**MYH15**	myosin heavy chain 15	2.364	3.76E-10
**MYH16**	myosin heavy chain 16 pseudogene	3.412	0.000175
**MYH3**	myosin heavy chain 3	2.483	0.000000769
**MYLK2**	myosin light chain kinase 2	2.415	9.16E-13
**MYO1D**	myosin ID	1.646	8.93E-10
**MYO5C**	myosin VC	-2.038	1.6E-14
**MYOM1**	myomesin 1	-3.455	0.0000616
**MYOSLID**		1.815	0.000181
**MYRF**	myelin regulatory factor	1.792	1.52E-18
**MZF1-AS1**	MZF1 antisense RNA 1	2.525	7.41E-09
**NACAD**	NAC alpha domain containing	-1.793	0.000000152
**NALCN**	sodium leak channel, non-selective	-2.354	6.78E-10
**NANOS1**	nanos C2HC-type zinc finger 1	4.114	0.000151
**NAP1L2**	nucleosome assembly protein 1 like 2	4.014	1.3E-20
**NAT8L**	N-acetyltransferase 8 like	2.338	0.002
**NCEH1**	neutral cholesterol ester hydrolase 1	1.592	1.17E-25
**NDRG1**	N-myc downstream regulated 1	1.766	9.39E-22
**NDUFV2**	NADH:ubiquinone oxidoreductase core subunit V2	2.969	8.38E-09
**NEDD4**	NEDD4 E3 ubiquitin protein ligase	-1.565	3.45E-19
**NEDD9**	neural precursor cell expressed, developmentally down-regulated 9	-1.63	3.14E-19
**NEGR1**	neuronal growth regulator 1	-1.797	8.82E-13
**NEO1**	neogenin 1	2.588	5.02E-55
**NES**	nestin	-3.165	1.02E-132

**NEUROG2**	neurogenin 2	-4.847	0.000000577
**NFASC**	neurofascin	2.207	9.76E-42
**NHS**	NHS actin remodeling regulator	-1.787	9.47E-09
**NIPAL2**	NIPA like domain containing 2	-1.941	0.000114
**NIPSNAP1**	nipsnap homolog 1	-2.235	5.17E-25
**NISCH**	nischarin	-1.712	4.62E-18
**NKAIN3**	sodium/potassium transporting ATPase interacting 3	-6.854	6.71E-12
**NME3**	NME/NM23 nucleoside diphosphate kinase 3	-2.837	0.00000532
**NNMT**	nicotinamide N-methyltransferase	1.944	7.01E-26
**NOG**	noggin	5.035	0.000000414
**NOL6**	nucleolar protein 6	-1.549	6.97E-17
**NOTCH1**	notch receptor 1	-1.972	0.00000429
**NPAS1**	neuronal PAS domain protein 1	2.012	0.00664
**NPAS2**	neuronal PAS domain protein 2	2.581	1.87E-37
**NPTXR**	neuronal pentraxin receptor	3.57	7.34E-13
**NPY1R**	neuropeptide Y receptor Y1	-3.858	2.11E-22
**NR1D1**	nuclear receptor subfamily 1 group D member 1	1.54	2.64E-14
**NR1H4**	nuclear receptor subfamily 1 group H member 4	-23.037	1.32E-49
**NRG2**	neuregulin 2	-1.876	5.2E-14
**NRIP3**	nuclear receptor interacting protein 3	1.64	5.85E-15
**NRSN2-AS1**	NRSN2 antisense RNA 1	2.599	0.00000168
**NRXN3**	neurexin 3	-2.553	3.14E-11
**NSUN5**	NOP2/Sun RNA methyltransferase 5	-1.648	0.00000189
**NT5C**	5', 3'-nucleotidase, cytosolic	-1.705	3.28E-08
**NT5DC2**	5'-nucleotidase domain containing 2	-1.875	9.09E-30
**NTN1**	netrin 1	3.519	7.4E-35
**NTN4**	netrin 4	2.895	2.11E-59
**NTNG1**	netrin G1	4.845	6.59E-71
**NTNG2**	netrin G2	2.751	3.37E-09
**NTRK2**	neurotrophic receptor tyrosine kinase 2	1.889	0.000000121
**NTRK3**	neurotrophic receptor tyrosine kinase 3	-3.101	1.15E-40
**NUDT11**	nudix hydrolase 11	3.194	1.02E-09
**NUDT16**	nudix hydrolase 16	-1.559	1.06E-16
**NUDT16L1**	nudix hydrolase 16 like 1	-1.655	0.00000947
**NUDT4**	nudix hydrolase 4	-1.546	5E-17
**NUP210**	nucleoporin 210	-1.827	2.9E-36
**NXPH4**	neurexophilin 4	2.286	2.25E-11

**OLFM1**	olfactomedin 1	3.971	0.000229
**OLFML2B**	olfactomedin like 2B	1.821	2.77E-19
**ONECUT1**	one cut homeobox 1	-4.508	0.000161
**OPLAH**	5-oxoprolinase, ATP-hydrolysing	-1.943	0.000318
**OPN3**	opsin 3	-1.607	0.000000451
**OSBPL10**	oxysterol binding protein like 10	-1.537	1.15E-17
**OTOGL**	otogelin like	-2.51	2.95E-18
**OTULINL**	OTU deubiquitinase with linear linkage specificity like	2.152	8.2E-11
**P3H2**	prolyl 3-hydroxylase 2	1.872	2.43E-35
**P4HA1**	prolyl 4-hydroxylase subunit alpha 1	1.613	1.17E-19
**P4HA2**	prolyl 4-hydroxylase subunit alpha 2	1.842	1.71E-29
**P4HA3**	prolyl 4-hydroxylase subunit alpha 3	3.319	0.0000226
**P4HTM**	prolyl 4-hydroxylase, transmembrane	-1.746	5.83E-13
**PADI2**	peptidyl arginine deiminase 2	-3.142	0.00145
**PALM2AKAP2**	PALM2 and AKAP2 fusion	1.637	4.96E-09
**PAPPA**	pappalysin 1	1.582	6.52E-11
**PAQR6**	progestin and adipoQ receptor family member 6	1.757	0.00000128
**PARM1**	prostate androgen-regulated mucin-like protein 1	29.036	4.38E-154
**PAWR**	pro-apoptotic WT1 regulator	-1.615	1.46E-13
**PAX8**	paired box 8	3.03	1.07E-29
**PAX8-AS1**	PAX8 antisense RNA 1	6.171	3.22E-23
**PBX4**	PBX homeobox 4	-2.588	4.27E-09
**PBXIP1**	PBX homeobox interacting protein 1	1.822	2.91E-21
**PCDH1**	protocadherin 1	2.063	2.03E-12
**PCDH10**	protocadherin 10	-2.038	2.25E-28
**PCDH18**	protocadherin 18	-1.713	0.00000656
**PCDH20**	protocadherin 20	-15.77	2.73E-265
**PCDHGC3**	protocadherin gamma subfamily C, 3	2.026	7.14E-37
**PCDHGC5**	protocadherin gamma subfamily C, 5	2.403	1.96E-17
**PDE1C**	phosphodiesterase 1C	-1.636	3.07E-28
**PDE2A**	phosphodiesterase 2A	4.094	4.79E-11
**PDE3A**	phosphodiesterase 3A	4.803	0.000108
**PDE4A**	phosphodiesterase 4A	-3.09	5.34E-41
**PDE4D**	phosphodiesterase 4D	1.666	2.5E-11
**PDE5A**	phosphodiesterase 5A	6.322	6.2E-10
**PDGFD**	platelet derived growth factor D	1.913	1.48E-23
**PDGFRL**	platelet derived growth factor receptor like	12.268	3.72E-17

**PDK4**	pyruvate dehydrogenase kinase 4	6.046	3.77E-30
**PDPN**	podoplanin	-2.411	0.000481
**PDZD4**	PDZ domain containing 4	2.672	8.04E-17
**PEG10**	paternally expressed 10	-2.047	1.61E-65
**PEG3**	paternally expressed 3	14.946	3.46E-52
**PER1**	period circadian regulator 1	1.652	3.05E-13
**PER3**	period circadian regulator 3	1.701	2.18E-21
**PGM5**	phosphoglucomutase 5	-2.547	0.00000413
**PI3**	peptidase inhibitor 3	14.175	0.00000337
**PICK1**	protein interacting with PRKCA 1	-2.245	2.59E-24
**PIEZO2**	piezo type mechanosensitive ion channel component 2	-3.945	1.4E-25
**PIGZ**	phosphatidylinositol glycan anchor biosynthesis class Z	3.724	1.16E-13
**PIK3CD-AS2**	PIK3CD antisense RNA 2	-3.207	0.000198
**PIK3CG**	phosphatidylinositol-4,5-bisphosphate 3-kinase catalytic subunit gamma	6.096	7.61E-09
**PINK1**	PTEN induced kinase 1	1.621	3.66E-12
**PITPNM3**	PITPNM family member 3	1.937	1.02E-12
**PKN1**	protein kinase N1	-1.643	1.49E-13
**PKP2**	plakophilin 2	-1.845	1.47E-20
**PKP3**	plakophilin 3	-4.249	0.000000994
**PLAAT4**	phospholipase A and acyltransferase 4	1.843	0.00000234
**PLAC8**	placenta associated 8	-5.335	3.99E-08
**PLAT**	plasminogen activator, tissue type	-2.06	4.11E-39
**PLAUR**	plasminogen activator, urokinase receptor	1.826	4.73E-29
**PLCB4**	phospholipase C beta 4	2.301	1.54E-16
**PLEKHA7**	pleckstrin homology domain containing A7	-2.138	1.31E-11
**PLEKHG4B**	pleckstrin homology and RhoGEF domain containing G4B	3.545	2.51E-11
**PLP1**	proteolipid protein 1	-3.082	0.00005
**PLPP4**	phospholipid phosphatase 4	-1.586	0.000000109
**PLSCR1**	phospholipid scramblase 1	-1.537	8.56E-14
**PLSCR4**	phospholipid scramblase 4	1.673	0.00000789
**PLXNA4**	plexin A4	3.621	5.71E-17
**PLXND1**	plexin D1	-1.575	2.03E-13
**PNMA2**	PNMA family member 2	2.731	3.43E-09
**PODXL**	podocalyxin like	1.662	2.52E-30
**PODXL2**	podocalyxin like 2	2.079	7.8E-16
**POLR2J4**	RNA polymerase II subunit J4, pseudogene	3.691	0.00000864
**POM121L9P**	POM121 transmembrane nucleoporin like 9, pseudogene	12.677	0.00000296

**POSTN**	periostin	-31.228	4.96E-40
**POU3F2**	POU class 3 homeobox 2	-1.668	4.05E-20
**PPFIA2**	PTPRF interacting protein alpha 2	-2.108	3.57E-09
**PPM1H**	protein phosphatase, Mg2+/Mn2+ dependent 1H	8.865	5.95E-24
**PPM1M**	protein phosphatase, Mg2+/Mn2+ dependent 1M	-2.315	1.12E-15
**PPP1R35**	protein phosphatase 1 regulatory subunit 35	-1.584	0.00000506
**PPP1R3B**	protein phosphatase 1 regulatory subunit 3B	1.815	3.88E-15
**PPP1R9A**	protein phosphatase 1 regulatory subunit 9A	-1.641	9.62E-13
**PPP2R2B**	protein phosphatase 2 regulatory subunit Bbeta	-2.134	1.04E-49
**PPP2R2C**	protein phosphatase 2 regulatory subunit Bgamma	13.437	1.03E-40
**PPP4R4**	protein phosphatase 4 regulatory subunit 4	5.002	4.21E-10
**PREX1**	phosphatidylinositol-3,4,5-trisphosphate dependent Rac exchange factor 1	1.946	6.53E-26
**PRKCZ**	protein kinase C zeta	2.462	1.12E-23
**PRKD1**	protein kinase D1	-1.613	8.21E-13
**PRKG1**	protein kinase cGMP-dependent 1	6.935	1.61E-13
**PRLR**	prolactin receptor	-1.819	2.93E-08
**PROM2**	prominin 2	5.333	1.53E-08
**PROX1**	prospero homeobox 1	-2.021	5.96E-12
**PRR16**	proline rich 16	2.149	6.96E-15
**PRSS23**	serine protease 23	1.863	5.64E-37
**PRSS35**	serine protease 35	-2.011	4.01E-27
**PRTN3**	proteinase 3	8.726	0.000713
**PRUNE2**	prune homolog 2 with BCH domain	-3.102	1.55E-27
**PSD4**	pleckstrin and Sec7 domain containing 4	2.519	2.16E-26
**PSG5**	pregnancy specific beta-1-glycoprotein 5	-5.356	0.000000121
**PSG9**	pregnancy specific beta-1-glycoprotein 9	-2.526	0.0000565
**PSMA2**	proteasome subunit alpha 2	1.601	0.00000489
**PTCH1**	patched 1	-2.251	1.47E-09
**PTK7**	protein tyrosine kinase 7 (inactive)	-1.628	7.96E-13
**PTN**	pleiotrophin	-1.615	4.31E-16
**PTPRB**	protein tyrosine phosphatase receptor type B	-2.343	1.51E-48
**PTPRD**	protein tyrosine phosphatase receptor type D	-2.278	0.00000198
**PTPRE**	protein tyrosine phosphatase receptor type E	2.33	1.4E-33
**PTPRG-AS1**	PTPRG antisense RNA 1	-1.904	0.000000046
**PTPRU**	protein tyrosine phosphatase receptor type U	4.707	5.61E-64
**PTPRZ1**	protein tyrosine phosphatase receptor type Z1	-2.066	3.21E-17
**PTTG1IP**	PTTG1 interacting protein	1.52	6.67E-23

**PTX3**	pentraxin 3	1.716	1.38E-17
**PXMP4**	peroxisomal membrane protein 4	-1.726	1.09E-10
**PXYLP1**	2-phosphoxylose phosphatase 1	1.675	0.000000181
**PYCR1**	pyrroline-5-carboxylate reductase 1	-1.792	2.75E-15
**RAB11FIP1**	RAB11 family interacting protein 1	3.243	0.000129
**RAB11FIP4**	RAB11 family interacting protein 4	-2.026	0.0000712
**RAB29**	RAB29, member RAS oncogene family	1.553	3.23E-14
**RAB3IL1**	RAB3A interacting protein like 1	1.738	0.00000104
**RAB42**	RAB42, member RAS oncogene family	-2.78	0.000011
**RAB9B**	RAB9B, member RAS oncogene family	1.74	0.00000273
**RAC3**	Rac family small GTPase 3	-1.66	0.000057
**RADIL**	Rap associating with DIL domain	4.819	0.0000861
**RADX**	RPA1 related single stranded DNA binding protein, X-linked	1.978	0.000000385
**RAET1G**	retinoic acid early transcript 1G	2.661	1.66E-09
**RANGRF**	RAN guanine nucleotide release factor	-2.267	3.79E-31
**RAP1GAP2**	RAP1 GTPase activating protein 2	3.607	7.98E-39
**RAPGEF3**	Rap guanine nucleotide exchange factor 3	1.897	0.000535
**RAPGEF4**	Rap guanine nucleotide exchange factor 4	-2.171	6.56E-08
**RARB**	retinoic acid receptor beta	-1.562	3.38E-11
**RASGRP3**	RAS guanyl releasing protein 3	-1.759	2.84E-15
**RBM47**	RNA binding motif protein 47	1.615	0.00000142
**RCAN1**	regulator of calcineurin 1	1.76	5.64E-27
**REEP1**	receptor accessory protein 1	2.165	0.000000235
**RFTN1**	raftlin, lipid raft linker 1	2.015	1.35E-11
**RFX8**	RFX family member 8, lacking RFX DNA binding domain	2.273	5.99E-25
**RGMA**	repulsive guidance molecule BMP co-receptor a	-3.467	6.98E-29
**RGS20**	regulator of G protein signaling 20	2.161	6.93E-18
**RGS3**	regulator of G protein signaling 3	1.604	1.92E-19
**RGS7**	regulator of G protein signaling 7	-2.269	6.01E-24
**RHBDL2**	rhomboid like 2	1.717	0.000233
**RHOBTB1**	Rho related BTB domain containing 1	-1.594	1.39E-12
**RHOBTB3**	Rho related BTB domain containing 3	-1.526	1.12E-14
**RHOJ**	ras homolog family member J	-2.328	7.48E-50
**RHOU**	ras homolog family member U	1.91	6.94E-20
**RHOV**	ras homolog family member V	3.036	1.88E-08
**RIMBP2**	RIMS binding protein 2	19.767	2.35E-97
**RIMKLA**	ribosomal modification protein rimK like family member A	1.814	0.000581

**RIMS3**	regulating synaptic membrane exocytosis 3	-2.057	5.57E-15
**RIN2**	Ras and Rab interactor 2	1.604	4.16E-19
**RIPOR2**	RHO family interacting cell polarization regulator 2	-4.657	1.62E-118
**RN7SL689P**	RNA, 7SL, cytoplasmic 689, pseudogene	2.649	0.000257
**RNF112**	ring finger protein 112	-3.492	2.85E-11
**RNF144A**	ring finger protein 144A	1.575	2.5E-11
**RNF152**	ring finger protein 152	2.16	0.000000102
**RNF157**	ring finger protein 157	-2.092	5.96E-26
**RNF24**	ring finger protein 24	2.335	4.89E-59
**ROR1**	receptor tyrosine kinase like orphan receptor 1	2.115	6.3E-17
**RORA**	RAR related orphan receptor A	4.863	2.14E-08
**ROS1**	ROS proto-oncogene 1, receptor tyrosine kinase	9.621	2.31E-95
**RP11_1246C191**		1.833	1.04E-08
**RP11_134G810**		1.976	0.0000147
**RP11_159D122**		1.82	6.91E-08
**RP11_21L232**		2.674	0.000046
**RP11_284F2110**		4.138	2.04E-73
**RP11_284F217**		3.318	1.08E-11
**RP11_284F219**		5.106	5.46E-12
**RP11_320M162**		-4.077	4.44E-08
**RP11_364B143**		-1.951	0.00159
**RP11_404J231**		-1.663	0.000000347
**RP11_4C204**		2.795	0.000606
**RP11_541N103**		1.873	0.000283
**RP11_77H95**		5.426	0.0000502
**RP11_888D104**		2.477	0.000768
**RP1_140K85**		2.846	0.000256
**RP4_773N104**		1.59	1.63E-08
**RP4_791C191**		-2.379	0.000187
**RPS6KA2**	ribosomal protein S6 kinase A2	3.774	9.82E-55
**RRAS2**	RAS related 2	1.552	5.77E-11
**RRBP1**	ribosome binding protein 1	1.54	5.59E-14
**RTL9**	retrotransposon Gag like 9	2.219	2.84E-23
**RTN2**	reticulon 2	2.505	9.4E-17
**RTN4RL1**	reticulon 4 receptor like 1	6.044	0.000000329
**RTN4RL2**	reticulon 4 receptor like 2	3.527	0.0000504
**RUVBL1**	RuvB like AAA ATPase 1	-1.766	7.89E-21

**S100A2**	S100 calcium binding protein A2	3.807	4.46E-68
**S100A3**	S100 calcium binding protein A3	2.288	0.000000338
**S100A4**	S100 calcium binding protein A4	3.119	4.4E-26
**S100B**	S100 calcium binding protein B	-3.54	6.36E-78
**SAA1**	serum amyloid A1	9.709	6.01E-11
**SALL4**	spalt like transcription factor 4	-17.198	0.0000015
**SAMD10**	sterile alpha motif domain containing 10	-1.711	0.00000168
**SAMD11**	sterile alpha motif domain containing 11	-1.862	0.00000126
**SAMD5**	sterile alpha motif domain containing 5	-3.018	4.55E-32
**SAMD9L**	sterile alpha motif domain containing 9 like	1.728	3.58E-18
**SCAP**	SREBF chaperone	-1.624	3.7E-13
**SCD**	stearoyl-CoA desaturase	1.643	1.63E-24
**SCNN1A**	sodium channel epithelial 1 alpha subunit	4.96	1.39E-27
**SCNN1B**	sodium channel epithelial 1 beta subunit	3.244	9.33E-08
**SCPEP1**	serine carboxypeptidase 1	1.675	2.76E-26
**SDC1**	syndecan 1	1.705	1.49E-18
**SEL1L3**	SEL1L family member 3	2.012	2.08E-41
**SELENOP**	selenoprotein P	3.23	7.1E-11
**SELL**	selectin L	2.228	0.000827
**SEMA3B**	semaphorin 3B	-5.284	1.92E-45
**SEMA3C**	semaphorin 3C	1.751	7.87E-30
**SEMA3F**	semaphorin 3F	15.338	2.24E-62
**SEMA4B**	semaphorin 4B	1.7	7.73E-21
**SEMA7A**	semaphorin 7A (John Milton Hagen blood group)	2.073	2.46E-42
**SEPTIN3**	septin 3	1.808	1.42E-08
**SEPTIN6**	septin 6	2.095	1.84E-19
**SERBP1P1**	SERPINE1 mRNA binding protein 1 pseudogene 1	-1.705	0.000000138
**SERINC2**	serine incorporator 2	8.826	1.28E-112
**SERPINA1**	serpin family A member 1	2.641	2.26E-18
**SERPINA5**	serpin family A member 5	3.492	2.07E-15
**SERPINE1**	serpin family E member 1	1.891	7.97E-34
**SERPINE2**	serpin family E member 2	1.944	2.14E-43
**SERPINH1**	serpin family H member 1	-2.076	7.29E-23
**SESN2**	sestrin 2	1.631	9.92E-08
**SETBP1**	SET binding protein 1	1.558	3.77E-10
**SEZ6L2**	seizure related 6 homolog like 2	2.524	1.64E-18
**SFRP1**	secreted frizzled related protein 1	1.72	1.57E-15

**SFTA1P**	surfactant associated 1, pseudogene	100.677	2.21E-08
**SGSH**	N-sulfoglucosamine sulfohydrolase	-2.181	1.42E-30
**SH3BGRL2**	SH3 domain binding glutamate rich protein like 2	2.066	2.15E-10
**SH3D21**	SH3 domain containing 21	-2.999	8.97E-37
**SH3GL3**	SH3 domain containing GRB2 like 3, endophilin A3	4.54	2.29E-08
**SH3PXD2A**	SH3 and PX domains 2A	2.046	6.73E-42
**SHC2**	SHC adaptor protein 2	-3.209	0.000000399
**SHISA2**	shisa family member 2	2.003	0.00000164
**SHISAL1**	shisa like 1	6.728	3.75E-16
**SIAE**	sialic acid acetylesterase	1.562	5.89E-12
**SIGLEC22P**	sialic acid binding Ig like lectin 22, pseudogene	10.781	1.06E-10
**SIK1/SIK1B**	salt inducible kinase 1	1.721	5.58E-13
**SIPA1**	signal-induced proliferation-associated 1	-2.071	0.000000153
**SLAIN1**	SLAIN motif family member 1	-1.562	3.86E-14
**SLC12A7**	solute carrier family 12 member 7	4.334	5.59E-31
**SLC12A8**	solute carrier family 12 member 8	-2.565	4.65E-09
**SLC15A2**	solute carrier family 15 member 2	2.545	0.0000289
**SLC16A14**	solute carrier family 16 member 14	-2.293	0.00000167
**SLC16A2**	solute carrier family 16 member 2	1.628	2.51E-23
**SLC16A7**	solute carrier family 16 member 7	-2.334	0.000309
**SLC16A9**	solute carrier family 16 member 9	-3.059	3.69E-68
**SLC17A5**	solute carrier family 17 member 5	1.595	3.12E-08
**SLC1A1**	solute carrier family 1 member 1	1.91	2.79E-10
**SLC20A1**	solute carrier family 20 member 1	1.519	5.97E-22
**SLC22A4**	solute carrier family 22 member 4	2.126	2.09E-13
**SLC25A23**	solute carrier family 25 member 23	2.019	4.32E-35
**SLC25A27**	solute carrier family 25 member 27	7.436	2.27E-12
**SLC25A28**	solute carrier family 25 member 28	-1.667	7.94E-15
**SLC25A29**	solute carrier family 25 member 29	1.731	2.86E-15
**SLC26A11**	solute carrier family 26 member 11	-2.256	1.2E-16
**SLC27A6**	solute carrier family 27 member 6	-3.04	0.000208
**SLC29A4**	solute carrier family 29 member 4	1.723	0.000791
**SLC2A12**	solute carrier family 2 member 12	7	8.06E-51
**SLC2A13**	solute carrier family 2 member 13	6.809	5.66E-27
**SLC2A3**	solute carrier family 2 member 3	1.608	7.01E-12
**SLC30A3**	solute carrier family 30 member 3	4.602	5.92E-10
**SLC35F1**	solute carrier family 35 member F1	-1.837	0.00012

**SLC37A2**	solute carrier family 37 member 2	2.422	7.71E-09
**SLC38A11**	solute carrier family 38 member 11	3.982	2.47E-28
**SLC38A2**	solute carrier family 38 member 2	1.714	6.14E-22
**SLC38A3**	solute carrier family 38 member 3	4.593	3.58E-29
**SLC39A8**	solute carrier family 39 member 8	1.825	2.95E-18
**SLC40A1**	solute carrier family 40 member 1	-2.673	0.000000272
**SLC43A1**	solute carrier family 43 member 1	1.772	0.00041
**SLC44A3**	solute carrier family 44 member 3	2.381	0.00106
**SLC44A5**	solute carrier family 44 member 5	-7.144	1.28E-24
**SLC7A11**	solute carrier family 7 member 11	-2.003	2.7E-25
**SLC8A1**	solute carrier family 8 member A1	4.034	3.02E-87
**SLCO4A1**	solute carrier organic anion transporter family member 4A1	5.766	7.93E-91
**SLCO5A1**	solute carrier organic anion transporter family member 5A1	-2.195	9.25E-17
**SLIT2**	slit guidance ligand 2	-1.801	1.23E-38
**SLIT3**	slit guidance ligand 3	-1.789	2.3E-15
**SLPI**	secretory leukocyte peptidase inhibitor	151.869	2.22E-154
**SMAD1**	SMAD family member 1	-1.569	0.000000554
**SMIM25**	small integral membrane protein 25	2.735	8.86E-10
**SMPDL3B**	sphingomyelin phosphodiesterase acid like 3B	3.108	0.0000339
**SMTN**	smoothelin	-1.607	5.75E-08
**SNHG19**	small nucleolar RNA host gene 19	-1.706	0.00000245
**SNHG25**		-2.296	0.00000186
**SNHG9**	small nucleolar RNA host gene 9	-1.671	0.000048
**SOCS2**	suppressor of cytokine signaling 2	-2.038	1.84E-31
**SOCS2-AS1**	SOCS2 antisense RNA 1	-1.882	2.54E-10
**SOCS5**	suppressor of cytokine signaling 5	1.565	4.54E-16
**SORBS2**	sorbin and SH3 domain containing 2	-1.556	5.96E-10
**SORBS3**	sorbin and SH3 domain containing 3	-1.589	2.27E-16
**SORCS2**	sortilin related VPS10 domain containing receptor 2	12.847	4.26E-94
**SOX13**	SRY-box transcription factor 13	-1.897	2.16E-32
**SOX2**	SRY-box transcription factor 2	-1.684	2.25E-16
**SOX2-OT**	SOX2 overlapping transcript	-5.92	1.18E-191
**SPAG17**	sperm associated antigen 17	-2.195	2.2E-22
**SPATC1L**	spermatogenesis and centriole associated 1 like	-2.395	6.71E-10
**SPINT1**	serine peptidase inhibitor, Kunitz type 1	-1.966	0.000272
**SPOCK1**	SPARC (osteonectin), cwcv and kazal like domains proteoglycan 1	-1.736	3.34E-30
**SPP1**	secreted phosphoprotein 1	-2.283	4.95E-61

**SPSB1**	splA/ryanodine receptor domain and SOCS box containing 1	3.28	1.33E-51
**SPTB**	spectrin beta, erythrocytic	1.621	0.000000256
**SPTBN4**	spectrin beta, non-erythrocytic 4	-3.629	0.000141
**SQOR**	sulfide quinone oxidoreductase	1.871	1.33E-14
**SRSF12**	serine and arginine rich splicing factor 12	-3.288	6.53E-08
**SSBP3**	single stranded DNA binding protein 3	1.551	1.91E-13
**SSC5D**	scavenger receptor cysteine rich family member with 5 domains	3.656	3.28E-16
**SSH3**	slingshot protein phosphatase 3	-1.571	1.58E-08
**ST8SIA1**	ST8 alpha-N-acetyl-neuraminide alpha-2,8-sialyltransferase 1	2.597	0.000362
**ST8SIA4**	ST8 alpha-N-acetyl-neuraminide alpha-2,8-sialyltransferase 4	2.396	0.000125
**STAC**	SH3 and cysteine rich domain	-1.687	7.87E-23
**STAMBPL1**	STAM binding protein like 1	-1.612	2.52E-13
**STARD4**	StAR related lipid transfer domain containing 4	1.764	0.000000331
**STC1**	stanniocalcin 1	1.966	1.43E-40
**STEAP1**	STEAP family member 1	4.484	1.5E-20
**STEAP2**	STEAP2 metalloreductase	2.513	0.00000971
**STK17A**	serine/threonine kinase 17a	1.798	1.21E-29
**STK32B**	serine/threonine kinase 32B	-1.905	1.88E-11
**STMN3**	stathmin 3	2.641	1.76E-25
**STOM**	stomatin	-1.907	1.26E-34
**STON2**	stonin 2	1.753	3.78E-12
**STRA6**	stimulated by retinoic acid 6	-2.502	2.43E-25
**STX1A**	syntaxin 1A	1.555	4.99E-09
**STXBP6**	syntaxin binding protein 6	1.755	0.0000001
**SULF1**	sulfatase 1	-6.024	7.78E-106
**SULF2**	sulfatase 2	1.628	3.75E-21
**SURF2**	surfeit 2	-1.647	0.00195
**SUSD1**	sushi domain containing 1	-2.177	7.78E-09
**SUSD2**	sushi domain containing 2	4.342	5.75E-16
**SUSD4**	sushi domain containing 4	2.333	0.00000178
**SUSD6**	sushi domain containing 6	1.553	1.31E-09
**SV2A**	synaptic vesicle glycoprotein 2A	-1.557	2.88E-11
**SYNC**	syncoilin, intermediate filament protein	1.591	4.69E-14
**SYNE3**	spectrin repeat containing nuclear envelope family member 3	-2.172	0.000118
**SYNGR1**	synaptogyrin 1	-2.895	2.01E-11
**SYNPO**	synaptopodin	2.261	3.17E-47
**SYTL2**	synaptotagmin like 2	2.276	5.05E-19

**TACR1**	tachykinin receptor 1	1.902	4.9E-14
**TAGLN**	transgelin	-1.707	0.00000949
**TBC1D4**	TBC1 domain family member 4	2.196	3.21E-35
**TBC1D8**	TBC1 domain family member 8	1.693	8.46E-10
**TBC1D8B**	TBC1 domain family member 8B	1.988	1.71E-09
**TCHH**	trichohyalin	2.622	0.000124
**TCN1**	transcobalamin 1	2.896	4.33E-23
**TDP1**	tyrosyl-DNA phosphodiesterase 1	-1.652	1.41E-15
**TDRP**	testis development related protein	1.634	0.0000115
**TEF**	TEF transcription factor, PAR bZIP family member	2.278	3.33E-38
**TENM2**	teneurin transmembrane protein 2	1.597	3.26E-23
**TENM4**	teneurin transmembrane protein 4	1.556	1.97E-13
**TESK2**	testis associated actin remodelling kinase 2	1.863	0.000000481
**TEX15**	testis expressed 15, meiosis and synapsis associated	3.867	0.00000574
**TEX9**	testis expressed 9	1.614	0.00000617
**TFAP2B**	transcription factor AP-2 beta	-2.351	0.000808
**TFAP2C**	transcription factor AP-2 gamma	-1.655	7.95E-28
**TFB1M**	transcription factor B1, mitochondrial	-1.576	9.48E-11
**TFCP2L1**	transcription factor CP2 like 1	2.958	8.95E-10
**TFEB**	transcription factor EB	2.023	8.39E-15
**TFPI**	tissue factor pathway inhibitor	1.613	3.24E-13
**TGFB2**	transforming growth factor beta 2	1.642	2.78E-14
**TGFB3**	transforming growth factor beta 3	2.487	3E-27
**TGFBR3**	transforming growth factor beta receptor 3	2.115	0.00228
**TGM2**	transglutaminase 2	-1.531	9.53E-13
**THEMIS2**	thymocyte selection associated family member 2	-4.431	0.000000736
**THNSL2**	threonine synthase like 2	-5.221	1.42E-14
**THSD1**	thrombospondin type 1 domain containing 1	2.725	6.68E-40
**TIAM1**	T cell lymphoma invasion and metastasis 1	1.752	3.87E-16
**TIMP3**	TIMP metallopeptidase inhibitor 3	1.564	1.79E-11
**TIMP4**	TIMP metallopeptidase inhibitor 4	1.767	0.000000222
**TKTL1**	transketolase like 1	4.137	2.4E-41
**TLR1**	toll like receptor 1	2.275	3.33E-09
**TLR3**	toll like receptor 3	2.388	0.000318
**TLR4**	toll like receptor 4	-1.777	9.19E-29
**TLR6**	toll like receptor 6	1.794	1.24E-14
**TM4SF18**	transmembrane 4 L six family member 18	2.881	7.03E-25

**TMC7**	transmembrane channel like 7	1.924	0.00000016
**TMEFF2**	transmembrane protein with EGF like and two follistatin like domains 2	1.873	8.99E-08
**TMEM100**	transmembrane protein 100	-4.2	1.13E-10
**TMEM143**	transmembrane protein 143	1.773	0.000000305
**TMEM158**	transmembrane protein 158 (gene/pseudogene)	3.944	7.84E-121
**TMEM171**	transmembrane protein 171	-3.871	1.06E-09
**TMEM173**	transmembrane protein 173	1.702	3.7E-09
**TMEM179**	transmembrane protein 179	4.897	3.82E-15
**TMEM200A**	transmembrane protein 200A	-2.303	0.000799
**TMEM200C**	transmembrane protein 200C	-2.099	1.28E-22
**TMEM205**	transmembrane protein 205	-1.701	1.46E-13
**TMEM220**	transmembrane protein 220	-2.363	0.000000766
**TMEM229B**	transmembrane protein 229B	-3.001	0.00000127
**TMEM25**	transmembrane protein 25	-3.12	1.18E-13
**TMEM255A**	transmembrane protein 255A	2.295	8.73E-08
**TMEM255B**	transmembrane protein 255B	9.693	9.56E-27
**TMEM26**	transmembrane protein 26	-2.605	0.00015
**TMEM38A**	transmembrane protein 38A	1.694	0.00000191
**TMEM45A**	transmembrane protein 45A	-1.532	1.14E-14
**TMEM51**	transmembrane protein 51	1.553	1.38E-09
**TMEM63C**	transmembrane protein 63C	-6.501	7.19E-08
**TMEM71**	transmembrane protein 71	2.699	5.45E-22
**TMSB15A**	thymosin beta 15a	4.421	2.51E-18
**TMTC1**	transmembrane O-mannosyltransferase targeting cadherins 1	2.138	8.45E-35
**TMTC4**	transmembrane O-mannosyltransferase targeting cadherins 4	-1.9	6.69E-24
**TNC**	tenascin C	1.697	6.6E-25
**TNFAIP3**	TNF alpha induced protein 3	1.55	5.86E-10
**TNFAIP6**	TNF alpha induced protein 6	3.289	0.000000937
**TNFRSF11B**	TNF receptor superfamily member 11b	222.216	5.42E-18
**TNFRSF21**	TNF receptor superfamily member 21	3.604	2.06E-118
**TNFSF10**	TNF superfamily member 10	3.24	2.4E-41
**TNFSF4**	TNF superfamily member 4	-11.65	3.61E-140
**TNNT1**	troponin T1, slow skeletal type	2.432	1.62E-16
**TOX**	thymocyte selection associated high mobility group box	2.204	1.1E-13
**TP53I11**	tumor protein p53 inducible protein 11	3.572	1.11E-82
**TPPP**	tubulin polymerization promoting protein	4.229	9.65E-22
**TPRG1**	tumor protein p63 regulated 1	3.311	4.12E-10

**TRABD2A**	TraB domain containing 2A	4.818	3.55E-57
**TRAPPC9**	trafficking protein particle complex 9	-1.818	2.01E-18
**TRERF1**	transcriptional regulating factor 1	1.58	2.58E-13
**TRIM24**	tripartite motif containing 24	-1.534	5.75E-18
**TRIM29**	tripartite motif containing 29	57.105	3.16E-17
**TRIM38**	tripartite motif containing 38	-1.82	1.85E-09
**TRIM6**	tripartite motif containing 6	3.217	6.42E-13
**TRNP1**	TMF1 regulated nuclear protein 1	1.853	1.16E-12
**TRPM3**	transient receptor potential cation channel subfamily M member 3	-4.248	2.79E-27
**TSHZ2**	teashirt zinc finger homeobox 2	6.009	3.59E-15
**TSKU**	tsukushi, small leucine rich proteoglycan	1.908	1.98E-15
**TSPAN1**	tetraspanin 1	3.103	2.66E-08
**TSPAN12**	tetraspanin 12	8.052	3.53E-13
**TSPAN13**	tetraspanin 13	1.602	4.77E-16
**TSPAN15**	tetraspanin 15	-1.618	1.19E-14
**TSPAN2**	tetraspanin 2	-1.736	3.54E-13
**TSPAN9**	tetraspanin 9	1.594	8.68E-18
**TSTD1**	thiosulfate sulfurtransferase like domain containing 1	-2.546	3.29E-15
**TTBK1**	tau tubulin kinase 1	-4.513	4.79E-29
**TTC39A**	tetratricopeptide repeat domain 39A	-2.705	0.000397
**TTC3P1**	tetratricopeptide repeat domain 3 pseudogene 1	2.416	6.44E-34
**TTC6**	tetratricopeptide repeat domain 6	-5.327	1.66E-09
**TTYH2**	tweety family member 2	1.961	0.000311
**TUBB2B**	tubulin beta 2B class IIb	-2.6	4.38E-27
**TUBB4A**	tubulin beta 4A class IVa	2.371	5.68E-09
**TXK**	TXK tyrosine kinase	-3.695	6.4E-16
**TYMSOS**	TYMS opposite strand	-2.258	0.00000234
**UBE2L6**	ubiquitin conjugating enzyme E2 L6	1.581	0.000000032
**UCA1**	urothelial cancer associated 1	161.643	6.16E-47
**UCP2**	uncoupling protein 2	1.622	2.21E-11
**UNC5B**	unc-5 netrin receptor B	2.1	0.000144
**USP43**	ubiquitin specific peptidase 43	3.451	0.0000197
**VAC14-AS1**	VAC14 antisense RNA 1	3.813	0.0000133
**VAT1L**	vesicle amine transport 1 like	-6.721	2.55E-21
**VCAM1**	vascular cell adhesion molecule 1	-2.68	9.51E-08
**VCAN**	versican	-1.625	1.96E-19
**VDR**	vitamin D receptor	2.198	1.79E-21

**VEGFA**	vascular endothelial growth factor A	2.153	9.84E-61
**VEGFC**	vascular endothelial growth factor C	1.619	1.88E-17
**VEPH1**	ventricular zone expressed PH domain containing 1	1.862	5.55E-13
**VGLL3**	vestigial like family member 3	-1.815	0.0000268
**VIT**	vitrin	-3.433	3.5E-33
**VMAC**	vimentin type intermediate filament associated coiled-coil protein	-1.648	1.92E-09
**VPS35L**	VPS35 endosomal protein sorting factor like	-2.131	4.57E-19
**VSIG10L**	V-set and immunoglobulin domain containing 10 like	2.263	0.0000763
**VSNL1**	visinin like 1	-1.793	3.62E-12
**VSTM4**	V-set and transmembrane domain containing 4	4.545	0.0000423
**VWA5A**	von Willebrand factor A domain containing 5A	2.46	8.12E-11
**VWCE**	von Willebrand factor C and EGF domains	3.982	2.19E-08
**VWDE**	von Willebrand factor D and EGF domains	-2.023	6.44E-16
**WIPI1**	WD repeat domain, phosphoinositide interacting 1	1.774	1.87E-11
**WNT5B**	Wnt family member 5B	2.005	8.21E-23
**WNT7B**	Wnt family member 7B	2.028	0.0000648
**WWC3**	WWC family member 3	-1.529	2E-14
**XAF1**	XIAP associated factor 1	2.54	0.000000137
**XBP1**	X-box binding protein 1	1.565	9.34E-15
**XDH**	xanthine dehydrogenase	4.296	3.26E-65
**XKR4**	XK related 4	-2.449	1.9E-14
**XPC**	XPC complex subunit, DNA damage recognition and repair factor	-1.529	3.52E-10
**YPEL3**	yippee like 3	-2.222	0.00000198
**ZBTB20**	zinc finger and BTB domain containing 20	-1.786	0.000229
**ZBTB46**	zinc finger and BTB domain containing 46	1.746	0.000716
**ZBTB47**	zinc finger and BTB domain containing 47	-1.599	0.0000187
**ZC3H12B**	zinc finger CCCH-type containing 12B	2.843	1.51E-08
**ZCWPW1**	zinc finger CW-type and PWWP domain containing 1	1.905	0.00000949
**ZDHHC23**	zinc finger DHHC-type containing 23	-1.554	1.06E-10
**ZEB1**	zinc finger E-box binding homeobox 1	1.534	1.12E-14
**ZFHX4-AS1**	ZFHX4 antisense RNA 1	-3.914	7.41E-26
**ZFPM2-AS1**	ZFPM2 antisense RNA 1	-1.913	0.00000706
**ZFYVE28**	zinc finger FYVE-type containing 28	2.372	0.000000808
**ZIC2**	Zic family member 2	8.119	1.08E-11
**ZIC5**	Zic family member 5	4.072	0.000000228
**ZMAT1**	zinc finger matrin-type 1	9.174	0.000000246
**ZMIZ1**	zinc finger MIZ-type containing 1	1.556	1.42E-15

**ZMIZ1-AS1**	ZMIZ1 antisense RNA 1	2.293	4.25E-11
**ZNF239**	zinc finger protein 239	-1.603	0.000019
**ZNF467**	zinc finger protein 467	10.496	0.00000151
**ZNF506**	zinc finger protein 506	-2.025	1.9E-12
**ZNF512**	zinc finger protein 512	2.105	1.97E-26
**ZNF536**	zinc finger protein 536	3.378	0.000000185
**ZNF620**	zinc finger protein 620	-1.966	1.41E-10
**ZNF660**	zinc finger protein 660	-5.464	1.3E-09
**ZNF665**	zinc finger protein 665	-2.087	0.000482
**ZNF702P**	zinc finger protein 702, pseudogene	-2.699	3.03E-09
**ZNF793**	zinc finger protein 793	-2.513	2.09E-10
**ZNF853**	zinc finger protein 853	-3.612	2.92E-29
**ZNF879**	zinc finger protein 879	1.973	0.0000116
**ZP1**	zona pellucida glycoprotein 1	3.13	0.00000888
**ZSCAN5A**	zinc finger and SCAN domain containing 5A	1.955	0.000000373
**ZSWIM4**	zinc finger SWIM-type containing 4	1.774	6.64E-14
**ZSWIM5**	zinc finger SWIM-type containing 5	-1.744	1.32E-09
