# Supplementary material for: mTORC2/Rac1 Pathway Predisposes Cancer Aggressiveness in IDH1-Mutated Glioma
Source: Cancers (Basel). 2020 Mar 26;12(4):787. doi: 10.3390/cancers12040787 (PMC7226122; doi:10.3390/cancers12040787)
Supplement: Supplementary file 1 [file cancers-12-00787-s001.zip › cancers-733430-revised-suppl-3.18/Supplementary Table 4.pdf]

Supplementary Table 4. List of differentially expressed cell movement related genes in U251 IDH1 R132C compared with U251 IDH1 WT

Gene Symbol	Entrez Gene Name	Fold Change	FDR
A2M	alpha-2-macroglobulin	3.563	6.56E-133
ABCA1	ATP binding cassette subfamily A member 1	1.722	2.68E-27
ABCB4	ATP binding cassette subfamily B member 4	-21.743	1.71E-09
ACKR3	atypical chemokine receptor 3	9.625	5.48E-16
ACTA2	actin alpha 2, smooth muscle	1.784	9.07E-13
ADGRG1	adhesion G protein-coupled receptor G1	1.776	1.13E-27
ADRB2	adrenoceptor beta 2	3.608	2.78E-11
AHNAK	AHNAK nucleoprotein	1.597	2.68E-14
AJAP1	adherens junctions associated protein 1	-1.892	0.000267
ALK	ALK receptor tyrosine kinase	-3.611	7.55E-17
AMOT	angiomotin	-1.883	1.14E-27
ANGPTL4	angiopoietin like 4	6.849	1.41E-21
APLN	apelin	-1.897	0.000000293
ARHGAP4	Rho GTPase activating protein 4	2.338	0.00107
ARHGAP24	Rho GTPase activating protein 24	24.153	6.13E-36
ARRB1	arrestin beta 1	2.74	3.02E-17
ASIC1	acid sensing ion channel subunit 1	1.884	4.82E-10
ATOH8	atonal bHLH transcription factor 8	-3.426	2.2E-10
ATP8A1	ATPase phospholipid transporting 8A1	4.495	0.00000661
BCAN	brevican	4.138	2.04E-73
BCAR3	BCAR3 adaptor protein, NSP family member	1.87	4.25E-34
BCAT1	branched chain amino acid transaminase 1	-1.565	1.62E-14
BHLHE40	basic helix-loop-helix family member e40	1.798	3.36E-31
BHLHE41	basic helix-loop-helix family member e41	2.204	2.43E-33
BMP4	bone morphogenetic protein 4	13.466	2.1E-16
BST2	bone marrow stromal cell antigen 2	3.008	0.000132
C3	complement C3	8.149	9.18E-33
C3AR1	complement C3a receptor 1	2.708	0.00015
CACNA1E	calcium voltage-gated channel subunit alpha1 E	5.434	0.000000298
CALCRL	calcitonin receptor like receptor	1.813	0.000799
CASP1	caspase 1	1.686	0.000112
CBS/CBSL	cystathionine beta-synthase	-1.611	0.00000209
CCN2	cellular communication network factor 2	-1.719	1.31E-41
CCN3	cellular communication network factor 3	8.762	0.000000414
CD55	CD55 molecule (Cromer blood group)	6.505	7.89E-34

CD82	CD82 molecule	3.469	5.08E-106
CD93	CD93 molecule	5.317	4.73E-13
CD200	CD200 molecule	-3.566	4.24E-24
CD274	CD274 molecule	-1.775	4.28E-21
CDKN1A	cyclin dependent kinase inhibitor 1A	2.463	1.04E-50
CEBPD	CCAAT enhancer binding protein delta	1.822	0.0016
CELSR2	cadherin EGF LAG seven-pass G-type receptor 2	-1.773	6.12E-21
CEMIP	cell migration inducing hyaluronidase 1	2.056	3.7E-15
CGA	glycoprotein hormones, alpha polypeptide	-4.097	6.74E-10
CHFR	checkpoint with forkhead and ring finger domains	-2.15	8.44E-14
CHI3L1	chitinase 3 like 1	106.661	0
CHST1	carbohydrate sulfotransferase 1	1.606	0.0000284
CLCA2	chloride channel accessory 2	7.544	8.21E-17
CLU	clusterin	2.3	3.06E-31
CMTM8	CKLF like MARVEL transmembrane domain containing 8	-2.096	1.11E-17
CNR1	cannabinoid receptor 1	-4.256	4.75E-127
CNTNAP2	contactin associated protein like 2	4.58	5.84E-22
COL11A1	collagen type XI alpha 1 chain	-1.771	3.56E-30
COL17A1	collagen type XVII alpha 1 chain	3.338	2.25E-76
CPEB1	cytoplasmic polyadenylation element binding protein 1	2.093	2.03E-09
CRMP1	collapsin response mediator protein 1	2.615	0.00000184
CRYAB	crystallin alpha B	-3.99	2.82E-91
CSF1	colony stimulating factor 1	1.911	2.78E-32
CSF2	colony stimulating factor 2	3.184	3.28E-09
CTNNA2	catenin alpha 2	-3.887	1.07E-48
CTNND2	catenin delta 2	-2.368	2.61E-26
CTSH	cathepsin H	1.948	7.06E-29
CTSS	cathepsin S	2.789	7.73E-22
CXADR	CXADR Ig-like cell adhesion molecule	1.947	1.92E-19
CXCL12	C-X-C motif chemokine ligand 12	6.535	1.97E-39
CYP2C8	cytochrome P450 family 2 subfamily C member 8	4.168	0.0000372
DAB1	DAB adaptor protein 1	-2.113	0.000701
DDIT4	DNA damage inducible transcript 4	1.64	4.43E-09
DGKE	diacylglycerol kinase epsilon	1.736	0.0000241
DIO2	iodothyronine deiodinase 2	-2.407	1.51E-27
DLC1	DLC1 Rho GTPase activating protein	2.454	5.33E-33
DLL1	delta like canonical Notch ligand 1	3.502	1.33E-14

DMBT1	deleted in malignant brain tumors 1	4.286	0.000000135
DNM1	dynamin 1	1.585	1.16E-17
DOCK3	dedicator of cytokinesis 3	1.69	0.000000233
DOCK4	dedicator of cytokinesis 4	2.152	8.42E-37
DRD2	dopamine receptor D2	1.596	2.41E-17
DSE	dermatan sulfate epimerase	2.15	1.46E-24
DUSP1	dual specificity phosphatase 1	1.538	7.83E-12
DUSP10	dual specificity phosphatase 10	1.904	5.14E-19
EBF1	EBF transcription factor 1	1.756	0.0000748
ECSCR	endothelial cell surface expressed chemotaxis and apoptosis regulator	5.299	5.33E-13
EDIL3	EGF like repeats and discoidin domains 3	2.962	5.07E-58
EDN1	endothelin 1	-2.198	4.08E-37
EDNRB	endothelin receptor type B	-1.653	1.8E-10
EEF1A2	eukaryotic translation elongation factor 1 alpha 2	2.135	1.18E-22
EFNA1	ephrin A1	2.769	1.33E-12
EFNA4	ephrin A4	1.642	0.0000207
ELMO1	engulfment and cell motility 1	1.675	1.11E-12
EN1	engrailed homeobox 1	-1.664	4.09E-08
ENPP2	ectonucleotide pyrophosphatase/phosphodiesterase 2	8.436	9.52E-10
EPAS1	endothelial PAS domain protein 1	2.353	4.19E-62
EPB41L4B	erythrocyte membrane protein band 4.1 like 4B	2.883	4.79E-37
EPHA3	EPH receptor A3	-2.238	3.18E-61
EPHA4	EPH receptor A4	-1.887	5E-10
EPHB2	EPH receptor B2	2.672	7.39E-82
ERAP1	endoplasmic reticulum aminopeptidase 1	1.659	1.85E-22
EYA2	EYA transcriptional coactivator and phosphatase 2	2.641	0.000207
F3	coagulation factor III, tissue factor	-1.814	6.8E-26
F11R	F11 receptor	-2.053	6.97E-15
FABP7	fatty acid binding protein 7	-1.763	7.25E-25
FAP	fibroblast activation protein alpha	3.139	1.05E-26
FAS	Fas cell surface death receptor	1.58	1.88E-11
FAT3	FAT atypical cadherin 3	-2.128	1.11E-29
FBLN5	fibulin 5	8.105	3.81E-10
FBN1	fibrillin 1	1.662	7.59E-24
FBXO4	F-box protein 4	-1.595	2.18E-08
FER	FER tyrosine kinase	-1.906	1.42E-19
FERMT3	fermitin family member 3	-2.183	0.0000879

FEZF1	FEZ family zinc finger 1	-3.705	0.00000267
FHL1	four and a half LIM domains 1	1.569	2.12E-17
FLRT2	fibronectin leucine rich transmembrane protein 2	2.523	5.77E-47
FN1	fibronectin 1	1.697	9.78E-28
FOSB	FosB proto-oncogene, AP-1 transcription factor subunit	2.626	1.07E-08
FOXA1	forkhead box A1	-1.943	1.24E-13
FOXF1	forkhead box F1	9.586	7.82E-33
FOXF2	forkhead box F2	2.32	2.83E-14
FOXG1	forkhead box G1	-1.723	2.56E-10
FOXQ1	forkhead box Q1	3.167	1.43E-13
FPR1	formyl peptide receptor 1	9.942	2.2E-53
FSCN1	fascin actin-bundling protein 1	3.05	2.99E-26
GATA2	GATA binding protein 2	-1.565	0.0000426
GATA3	GATA binding protein 3	-1.869	3.06E-18
GCNT1	glucosaminyl (N-acetyl) transferase 1	1.863	1.68E-30
GGT5	gamma-glutamyltransferase 5	4.015	0.000145
GJA1	gap junction protein alpha 1	1.752	3.97E-37
GJB2	gap junction protein beta 2	2.138	5.13E-51
GNAO1	G protein subunit alpha o1	2.712	1.68E-24
GPM6A	glycoprotein M6A	-6.968	2.93E-213
GSN	gelsolin	-1.654	1.25E-13
HAS3	hyaluronan synthase 3	3.73	4.34E-100
HBEGF	heparin binding EGF like growth factor	1.54	1.97E-13
HDAC9	histone deacetylase 9	-1.874	1.02E-30
HEY2	hes related family bHLH transcription factor with YRPW motif 2	3.85	4.08E-09
HMOX1	heme oxygenase 1	2.063	8.58E-43
HOXA7	homeobox A7	-2.562	0.0000462
HRH2	histamine receptor H2	22.904	2.61E-11
HTRA1	HtrA serine peptidase 1	1.637	1.22E-20
ICOSLG/LOC102723996	inducible T cell costimulator ligand	2.463	2.05E-36
ID3	inhibitor of DNA binding 3, HLH protein	-1.602	1.42E-09
IDO1	indoleamine 2,3-dioxygenase 1	5.39	2.38E-16
IGFBP2	insulin like growth factor binding protein 2	-1.651	1.1E-12
IGFBP3	insulin like growth factor binding protein 3	3.696	3.52E-125
IGFBP4	insulin like growth factor binding protein 4	11.214	0
IGFBP5	insulin like growth factor binding protein 5	-2.43	1.78E-64
IGFBP6	insulin like growth factor binding protein 6	1.699	3.04E-08

IL7	interleukin 7	2.553	5.47E-10
IL11	interleukin 11	2.713	1.05E-38
IL15	interleukin 15	1.911	0.00000469
IL24	interleukin 24	11.493	0.000000015
IL13RA2	interleukin 13 receptor subunit alpha 2	2.714	1.73E-60
IL15RA	interleukin 15 receptor subunit alpha	2.697	4.51E-12
IL27RA	interleukin 27 receptor subunit alpha	1.964	1.95E-15
IL4R	interleukin 4 receptor	5.355	2.26E-51
ITGA1	integrin subunit alpha 1	2.22	6.49E-24
ITGA2	integrin subunit alpha 2	2.636	3.65E-71
ITGA9	integrin subunit alpha 9	-1.728	1.47E-10
ITGB2	integrin subunit beta 2	2.405	0.00000294
ITGB3	integrin subunit beta 3	1.576	6.47E-13
ITGB4	integrin subunit beta 4	1.548	3.65E-10
ITGB8	integrin subunit beta 8	-1.584	1.01E-15
ITGBL1	integrin subunit beta like 1	-2.171	1.12E-21
JAG2	jagged canonical Notch ligand 2	5.436	3.04E-17
JUP	junction plakoglobin	-2.291	5.02E-09
KALRN	kalirin RhoGEF kinase	1.784	3.52E-12
KCNK5	potassium two pore domain channel subfamily K member 5	78.236	7.63E-40
KCNMA1	potassium calcium-activated channel subfamily M alpha 1	2.784	8.59E-119
KCNN4	potassium calcium-activated channel subfamily N member 4	2.002	3.57E-29
KDM5B	lysine demethylase 5B	1.675	2.3E-22
KIAA0319	KIAA0319	1.699	0.0000179
KIRREL3	kirre like nephrin family adhesion molecule 3	-1.586	0.000000167
KITLG	KIT ligand	2.171	2.84E-29
KLF17	Kruppel like factor 17	5.808	1.86E-18
L1CAM	L1 cell adhesion molecule	4.844	1.93E-99
LAMA1	laminin subunit alpha 1	2.639	0.000093
LAMA2	laminin subunit alpha 2	2.611	1.1E-14
LAMC2	laminin subunit gamma 2	2.977	2.54E-21
LCP1	lymphocyte cytosolic protein 1	1.71	7.12E-15
LDHA	lactate dehydrogenase A	1.667	2.89E-13
LGR4	leucine rich repeat containing G protein-coupled receptor 4	3.779	8.68E-63
LIPE	lipase E, hormone sensitive type	-2.664	4.79E-11
LMCD1	LIM and cysteine rich domains 1	-2.107	1.33E-22
LOX	lysyl oxidase	9.077	1.32E-160

LRP1	LDL receptor related protein 1	1.567	1.15E-11
LSP1	lymphocyte specific protein 1	5.478	0.000000169
LTBP2	latent transforming growth factor beta binding protein 2	1.662	2.59E-08
MAFB	MAF bZIP transcription factor B	2.972	0.000243
MAGI2	membrane associated guanylate kinase, WW and PDZ domain containing 2	-1.734	1.11E-12
MATN2	matrilin 2	3.153	6.26E-108
MC1R	melanocortin 1 receptor	2.066	2.06E-08
MCF2	MCF.2 cell line derived transforming sequence	-5.678	0.0000211
MDGA1	MAM domain containing glycosylphosphatidylinositol anchor 1	2.486	3.48E-48
MDK	midkine	-4.537	3.19E-33
MEF2C	myocyte enhancer factor 2C	-1.722	2.54E-10
MELTF	melanotransferrin	-1.943	1.67E-08
MGLL	monoglyceride lipase	2.107	4.07E-53
MGP	matrix Gla protein	2.171	3.23E-39
MME	membrane metalloendopeptidase	4.053	1.72E-72
MMP1	matrix metallopeptidase 1	9.258	1.88E-08
MMP2	matrix metallopeptidase 2	1.663	7.65E-23
MMP3	matrix metallopeptidase 3	6.528	6.1E-21
MMP7	matrix metallopeptidase 7	2.754	6.64E-38
MMP14	matrix metallopeptidase 14	2.842	2.19E-100
MSX2	msh homeobox 2	1.804	0.000000333
MTSS1	MTSS I-BAR domain containing 1	12.624	9.27E-67
MTUS1	microtubule associated scaffold protein 1	6.961	0.000000487
MUC1	mucin 1, cell surface associated	1.92	0.0000115
MYD88	MYD88 innate immune signal transduction adaptor	-1.525	3.33E-14
MYRF	myelin regulatory factor	1.792	1.52E-18
NDRG1	N-myc downstream regulated 1	1.766	9.39E-22
NEDD4	NEDD4 E3 ubiquitin protein ligase	-1.565	3.45E-19
NEDD9	neural precursor cell expressed, developmentally down-regulated 9	-1.63	3.14E-19
NEO1	neogenin 1	2.588	5.02E-55
NES	nestin	-3.165	1.02E-132
NEUROG2	neurogenin 2	-4.847	0.000000577
NISCH	nischarin	-1.712	4.62E-18
NOG	noggin	5.035	0.000000414
NOTCH1	notch receptor 1	-1.972	0.00000429
NR1D1	nuclear receptor subfamily 1 group D member 1	1.54	2.64E-14
NR1H4	nuclear receptor subfamily 1 group H member 4	-23.037	1.32E-49

NTN1	netrin 1	3.519	7.4E-35
NTN4	netrin 4	2.895	2.11E-59
NTRK2	neurotrophic receptor tyrosine kinase 2	1.889	0.000000121
NTRK3	neurotrophic receptor tyrosine kinase 3	-3.101	1.15E-40
NUDT16L1	nudix hydrolase 16 like 1	-1.655	0.00000947
ONECUT1	one cut homeobox 1	-4.508	0.000161
P4HA2	prolyl 4-hydroxylase subunit alpha 2	1.842	1.71E-29
PAPPA	pappalysin 1	1.582	6.52E-11
PCDH10	protocadherin 10	-2.038	2.25E-28
PDE2A	phosphodiesterase 2A	4.094	4.79E-11
PDGFD	platelet derived growth factor D	1.913	1.48E-23
PDPN	podoplanin	-2.411	0.000481
PER1	period circadian regulator 1	1.652	3.05E-13
PICK1	protein interacting with PRKCA 1	-2.245	2.59E-24
PIK3CG	phosphatidylinositol-4,5-bisphosphate 3-kinase catalytic subunit gamma	6.096	7.61E-09
PKN1	protein kinase N1	-1.643	1.49E-13
PKP2	plakophilin 2	-1.845	1.47E-20
PLAT	plasminogen activator, tissue type	-2.06	4.11E-39
PLAUR	plasminogen activator, urokinase receptor	1.826	4.73E-29
PLP1	proteolipid protein 1	-3.082	0.00005
PLXND1	plexin D1	-1.575	2.03E-13
PODXL2	podocalyxin like 2	2.079	7.8E-16
PODXL	podocalyxin like	1.662	2.52E-30
POSTN	periostin	-31.228	4.96E-40
POU3F2	POU class 3 homeobox 2	-1.668	4.05E-20
PREX1	phosphatidylinositol-3,4,5-trisphosphate dependent Rac exchange factor 1	1.946	6.53E-26
PRKCZ	protein kinase C zeta	2.462	1.12E-23
PRKD1	protein kinase D1	-1.613	8.21E-13
PRKG1	protein kinase cGMP-dependent 1	6.935	1.61E-13
PRLR	prolactin receptor	-1.819	2.93E-08
PROX1	prospero homeobox 1	-2.021	5.96E-12
PRTN3	proteinase 3	8.726	0.000713
PTN	pleiotrophin	-1.615	4.31E-16
PTPRU	protein tyrosine phosphatase receptor type U	4.707	5.61E-64
PTPRZ1	protein tyrosine phosphatase receptor type Z1	-2.066	3.21E-17
PTX3	pentraxin 3	1.716	1.38E-17
RAB11FIP1	RAB11 family interacting protein 1	3.243	0.000129

RAC3	Rac family small GTPase 3	-1.66	0.000057
RADIL	Rap associating with DIL domain	4.819	0.0000861
RAPGEF3	Rap guanine nucleotide exchange factor 3	1.897	0.000535
RCAN1	regulator of calcineurin 1	1.76	5.64E-27
RGS3	regulator of G protein signaling 3	1.604	1.92E-19
RHOU	ras homolog family member U	1.91	6.94E-20
RIPOR2	RHO family interacting cell polarization regulator 2	-4.657	1.62E-118
ROR1	receptor tyrosine kinase like orphan receptor 1	2.115	6.3E-17
RRAS2	RAS related 2	1.552	5.77E-11
RUVBL1	RuvB like AAA ATPase 1	-1.766	7.89E-21
S100A2	S100 calcium binding protein A2	3.807	4.46E-68
S100A4	S100 calcium binding protein A4	3.119	4.4E-26
S100B	S100 calcium binding protein B	-3.54	6.36E-78
SAA1	serum amyloid A1	9.709	6.01E-11
SCNN1A	sodium channel epithelial 1 alpha subunit	4.96	1.39E-27
SCPEP1	serine carboxypeptidase 1	1.675	2.76E-26
SDC1	syndecan 1	1.705	1.49E-18
SELL	selectin L	2.228	0.000827
SEMA3B	semaphorin 3B	-5.284	1.92E-45
SEMA3C	semaphorin 3C	1.751	7.87E-30
SEMA3F	semaphorin 3F	15.338	2.24E-62
SERPINA1	serpin family A member 1	2.641	2.26E-18
SERPINA5	serpin family A member 5	3.492	2.07E-15
SERPINE1	serpin family E member 1	1.891	7.97E-34
SERPINE2	serpin family E member 2	1.944	2.14E-43
SERPINH1	serpin family H member 1	-2.076	7.29E-23
SFRP1	secreted frizzled related protein 1	1.72	1.57E-15
SH3PXD2A	SH3 and PX domains 2A	2.046	6.73E-42
SLC8A1	solute carrier family 8 member A1	4.034	3.02E-87
SLIT2	slit guidance ligand 2	-1.801	1.23E-38
SLIT3	slit guidance ligand 3	-1.789	2.3E-15
SLPI	secretory leukocyte peptidase inhibitor	151.869	2.22E-154
SMAD1	SMAD family member 1	-1.569	0.000000554
SOCS2	suppressor of cytokine signaling 2	-2.038	1.84E-31
SORBS3	sorbin and SH3 domain containing 3	-1.589	2.27E-16
SOX2	SRY-box transcription factor 2	-1.684	2.25E-16
SPINT1	serine peptidase inhibitor, Kunitz type 1	-1.966	0.000272

SPP1	secreted phosphoprotein 1	-2.283	4.95E-61
SPSB1	splA/ryanodine receptor domain and SOCS box containing 1	3.28	1.33E-51
SRSF1	serine and arginine rich splicing factor 1	1.82	6.91E-08
ST8SIA4	ST8 alpha-N-acetyl-neuraminide alpha-2,8-sialyltransferase 4	2.396	0.000125
STC1	stanniocalcin 1	1.966	1.43E-40
STMN3	stathmin 3	2.641	1.76E-25
SULF1	sulfatase 1	-6.024	7.78E-106
TACR1	tachykinin receptor 1	1.902	4.9E-14
TFAP2C	transcription factor AP-2 gamma	-1.655	7.95E-28
TFPI	tissue factor pathway inhibitor	1.613	3.24E-13
TGFB2	transforming growth factor beta 2	1.642	2.78E-14
TGFB3	transforming growth factor beta 3	2.487	3E-27
TGFBR3	transforming growth factor beta receptor 3	2.115	0.00228
TGM2	transglutaminase 2	-1.531	9.53E-13
TIAM1	T cell lymphoma invasion and metastasis 1	1.752	3.87E-16
TIMP3	TIMP metallopeptidase inhibitor 3	1.564	1.79E-11
TLR3	toll like receptor 3	2.388	0.000318
TLR4	toll like receptor 4	-1.777	9.19E-29
TMEFF2	transmembrane protein with EGF like and two follistatin like domains 2	1.873	8.99E-08
TNC	tenascin C	1.697	6.6E-25
TNFAIP3	TNF alpha induced protein 3	1.55	5.86E-10
TNFAIP6	TNF alpha induced protein 6	3.289	0.000000937
TNFRSF21	TNF receptor superfamily member 21	3.604	2.06E-118
TNFRSF11B	TNF receptor superfamily member 11b	222.216	5.42E-18
TNFSF4	TNF superfamily member 4	-11.65	3.61E-140
TNFSF10	TNF superfamily member 10	3.24	2.4E-41
TUBB2B	tubulin beta 2B class IIb	-2.6	4.38E-27
TXK	TXK tyrosine kinase	-3.695	6.4E-16
UCP2	uncoupling protein 2	1.622	2.21E-11
UNC5B	unc-5 netrin receptor B	2.1	0.000144
VCAM1	vascular cell adhesion molecule 1	-2.68	9.51E-08
VCAN	versican	-1.625	1.96E-19
VDR	vitamin D receptor	2.198	1.79E-21
VEGFA	vascular endothelial growth factor A	2.153	9.84E-61
VEGFC	vascular endothelial growth factor C	1.619	1.88E-17
VSNL1	visinin like 1	-1.793	3.62E-12
WNT5B	Wnt family member 5B	2.005	8.21E-23

XDH	xanthine dehydrogenase	4.296	3.26E-65
ZEB1	zinc finger E-box binding homeobox 1	1.534	1.12E-14
